# Genetic diversity, population structure, and combined detection of selection signatures in Iranian versus Afghan Baluchi sheep

**DOI:** 10.1371/journal.pone.0350262

**Published:** 2026-06-17

**Authors:** Sadegh Taheri, Mohammad Osman Karimi, Naghmeh Saedi, Saeed Zerehdaran, Mohammad Mahdi Shariati, Mohsen Gholizadeh, Ali Javadmanesh

**Affiliations:** 1 Department of Animal Science, Faculty of Agriculture, Ferdowsi University of Mashhad, Mashhad, Iran; 2 Department of Animal Science, Faculty of Agriculture, Herat University, Herat, Afghanistan; 3 Center for Quantitative Genetics and Genomics, Aarhus University, Tjele, Denmark; 4 Department of Animal Science, Sari Agriculture and Natural Resources University, Sari, Iran; West Bengal University of Animal and Fishery Sciences, INDIA

## Abstract

Selection to increase the frequency of useful mutations has left marks on animal genomes, genetic diversity, and population structure within populations. The study and investigation of these genomic regions can lead to the identification of genes related to economic traits or competence and adaptability. This study aimed to recognize genetic diversity, population structure, and selection signatures in Iranian (IB) and Afghan (AB) Baluchi sheep populations. In this study, 86 Iranian Baluchi and 15 Afghan Baluchi sheep were genotyped using Illumine Ovine SNP50K Beadchip arrays. Note that the sample size imbalance (IB n = 86 vs. AB n = 15) may reduce statistical power and potentially bias population structure and selection scan results. Additionally, use of the Ovine 50K array may introduce ascertainment bias; analyses were based on 38,193 shared SNPs, potentially missing population-specific variants. Generally, moderate genetic diversity was observed in both the Afghan Baluchi (AB) and Iranian Baluchi (IB) sheep populations, using various assessment methods. However, the IB population showed the lowest level of genetic diversity and the highest rate of linkage disequilibrium decay, despite having a better effective population size in recent generations. The ADMIXTURE analysis indicated that the optimal number of genetic clusters was K = 2, which was determined based on the lowest cross-entropy error of 0.603 observed during cross-validation. At K = 2 and NJ tree analysis, a clear genetic distinction between the AB and IB populations was evident. Additionally, the IB population demonstrated significant genetic uniformity when compared to the AB population in terms of genetic distance. Also, F_ST_ and XP-EHH were used to identify selection signatures. Some putative candidate genes for F_ST,_ including *HDAC9*, *CSMD3*, *DAB1*, *FGF12*, and *PCDH9* were associated with important economic traits such as body weight, hot carcass weight, muscle weight in carcass, reproductive seasonality, and carcass fat percentage, respectively. Also, XP-EHH putative candidate genes were *KCNIP4*, *FGF11*, *CNTROB*, and *ROBO2* in AB population, which were related to body weight, hot carcass weight, milk yield, and muscle weight in carcass. Moreover, XP-EHH putative candidate genes in IB population were *GRIK3*, *NCOA1*, and *FGD3,* that related to muscle weight in carcass, staple length, and milk fat percentage. Selection signals were identified using top 1% F_ST_ thresholds and XP-EHH without genome-wide multiple-testing correction; results require experimental validation. We observed very similar outcomes in terms of similar signatures related to economic traits in both F_ST_ and XP-EHH methods, indicating the robustness of analysis in this study. It can be concluded that selection has made a major distinction between Afghan and Iranian Baluchi sheep populations for reproduction, milk production, and growth traits. These could be due to the managed breeding programme in Iranian Baluchi sheep. Utilizing validated QTLs as described in this study could be applied to reveal the direction of breeding plans in livestock species.

## Introduction

One of the primary grazing animals domesticated was sheep [1], due to its manageable size and the ability to adapt to various climates and poor nutrition diets. Artificial selection created a wide variety of breeds with respect to coat colour, distinct morphology, or particular production traits, among others. Natural selection (such as immunocompetence) has also put selection pressure on sheep. The Baluchi population, a major sheep breed in Iran regarding population size, is well-adapted to harsh climate conditions in central and eastern Iran with low-quality pastures. This breed is native to eastern and central regions of Iran, southwest of Pakistan, and southern Afghanistan. The fleece in Afghan Baluchi (AB) and Iranian Baluchi (IB) populations are coarse and white with black pigmentations at distal body parts (S1 Fig). Baluchi sheep is a fat-tailed breed, and it is very well adapted to a wide range of harsh environments and poor pasture quality in eastern Iran, a subtropical arid climate. Iranian Baluchi body size varies between 35 and 40 kg in adult ewes, milk yield between 40 and 50 kg in 125 days of milking, and annual greasy fleece weight of 1.3–1.8 kg (https://breeds.okstate.edu/sheep/baluchi-sheep.html). This breed has been under selection for important economic traits such as growth, milk production, etc in Abbas Abad Animal Breeding Centre, Mashhad, Iran, for more than four decades [2]. Also, the breeding of sheep in Afghanistan dates back to about 9000 years ago, when these valuable livestock species were domesticated [3]. As it is clear, around 2/3 of the population in Afghanistan is engaged in agriculture, particularly in the field of livestock [4]. Despite the importance of sheep production in Afghanistan, the lack of phenotypic records and accurate pedigree is the main issues that restrict the application of modern animal breeding programs. Afghan Baluchi fleece weight is around 1.3–2 Kg and the average body weight is 34–36 Kg (categorized as a meat-wool type breed). The average milk yield during the four months of a lactation period is about 40 Kg. It produces good-quality wool, suitable for carpet [4]. However, there is no evidence of breeding programs for Baluchi population in Afghanistan. It might be interesting to compare the impact of programmed and traditional selection methods on genomes of both populations, since it is believed that Iranian and Afghan Baluchi sheep populations has a common ancestor [5]. Both natural and artificial selection may have differential markers in specific genomic regions in domesticated animals. Therefore, the elucidation of certain genomic regions that show selection signatures may help to understand the process of biological selection and recognize potential candidate genes of interest [6].

Although the cost of genome sequencing is steadily decreasing, genotyping all individuals in a large population is still too expensive, which impedes the use of this technology. The SNP chip, which is a powerful tool for use in genetic studies, is a less expensive genotyping technology. A commercial 50K SNP chip for sheep genotyping became available in 2009 (http://www.illumina.com), providing a versatile, proficient, and cost-effective tool to detect positive selection signatures in the sheep genome [6,7].

Genetic diversity is a crucial aspect of overall biodiversity, alongside ecosystems and species, and is vital for the evolution of populations and species in the face of environmental and climatic changes. It also enhances the adaptability of animals to their habitats [8,9]. To preserve genetic diversity, it is essential to manage inbreeding and common ancestry, as noted by Gebreselase et al. [10]. However, in recent decades, traditional local breeding practices have declined due to the widespread use of commercial breeds specialized for production and heavy reliance on a limited number of rams, along with modern selection techniques [11]. Despite numerous studies focusing on genetic diversity and selection in sheep, Gorgol et al. point out a lack of genomic-level data on the genetic background and diversity of local sheep populations [12]. The presence of many local populations exhibiting distinct phenotypic differences warrants further research into their genetic differentiation. According to Meermans et al., local sheep breeds play an integral role in cultural heritage, are essential to local economies, possess unique traits adapted to regional climates, and are critical for sustainable and resilient agricultural systems [13].

The fixation index (F_ST_) and cross-population extended haplotype heterozygosity test (XP-EHH) statistics are used to identify selection signatures within individual samples from a given population. XP-EHH was proposed to identify selection signals between populations [14]. The XP-EHH statistic was designed by comparing haplotypes from two populations in order to recognize ongoing or almost fixed selection signatures [14]. Sabeti et al. (2002) reported XP-EHH as an extension of the EHH [15]. F_ST_, initially defined to measure the genetic divergence across populations [16], is an alternative statistic to recognize selection signatures among populations [17].

To classify genome regions under selection, F_ST_ has been formerly applied to different sheep populations by several studies [18,19]. Several genomic regions related to body size, reproductive performance, morphological traits, and skeletal morphology have been identified in these studies, which have been targeted via both artificial and natural selection during domestication [20].

Recently, QTL databases have been well developed due to advances in livestock genomics research and high-throughput genomics technologies. They could be linked to the most recent version of reference genomes such as sheep, cow, pig, fish, etc. [21]. These fine-mapped QTLs could provide accurate coordinates of genes and genomic regions related to known traits in livestock species. Therefore, researchers could pinpoint selection signatures to desired traits in livestock species [22].

This study aimed to assess genetic diversity, population structure, and identify regions under selection in each population and different regions between the populations using the Illumina OvineSNP50K Genotyping BeadChip array, and putative candidate genes were associated with these regions in Iranian and Afghan Baluchi populations of sheep.

## Materials and methods

### Sampling and genotyping

All experimental protocols were approved by the Biomedical Ethics Committee at Ferdowsi University of Mashhad with approval number 2/58110. Genotypes were obtained from previously published studies where all procedures were conducted in strict accordance with relevant guidelines and regulations, including the ARRIVE guidelines (Animal Research: Reporting of In Vivo Experiments). Our re-analysis of these public datasets followed standard bioinformatics practices. In this study, the genotype data of 101 unrelated animals, including 86 Iranian Baluchi and 15 Afghan Baluchi sheep, were used to identify selection signatures. Iranian Baluchi samples were collected from Abbas Abad Breeding Centre, Mashhad, Iran, and Afghan Baluchi samples were collected from different areas of Herat province. The IB flock was specifically selected as the most reliable source of purebred IB genetics, given the lack of documented purity information for Baluchi populations scattered across eastern and southern Iran, where field animals are typically maintained as crossbreeds or grade flocks. While the IB sample (n = 86) adequately represents this managed breeding population, it may not fully capture the broader geographic diversity of Iranian Baluchi sheep. The limited AB sample size reflects substantial logistical challenges in Afghanistan, where no dedicated Baluchi breeding centers exist, making identifying additional purebred animals infeasible. Also, due to extensive crossbreeding among Afghan sheep populations, finding pure Afghan Baluchi was very difficult; therefore, our sample size was limited. Iranian Baluchi genotype data were obtained from a study by Gholizadeh et al. (2015) (Supporting information IB) [23], and Afghan Baluchi data were obtained from Karimi et al. (2016) (Supporting information AB) [24]. Blood samples were taken in EDTA-containing tubes and kept in −20 °C until further use. DNA was extracted using GenElute™ Blood Genomic DNA Kit. Genotyping was performed by Ovine SNP50K Beadchip array (Illumina Oar_v4.0 assembly) in both groups, with all SNP coordinates mapped to this reference genome.

### Quality control and population structure

PLINK V1.9 software was used to control the quality of genotype data of IB and AB populations. Animals with genotyping call rate lower than 99% and SNPs with genotyping call rate below 99% and minor allele frequency lower than 5% were removed. SNPs with a large deviation from Hardy-Weinberg equilibrium (P < 10^6^) were removed as well [25]. Also, to control for inbreeding, examined pairs with a very high PI_HAT value (above 0.5) using the IBD test in PLINK version 1.9. In the Iranian Baluchi population, found only one pair that exhibited first-degree consanguinity. Therefore, one individual from this pair was excluded and the impact of this exclusion through a sensitivity analysis was evaluated. The principal component analysis was performed to screen population structure between and within breeds. Principal component analysis (PCA) was performed on SNPs using PLINK V1.9 to identify genetic relationships among breeds [26]. The graphical representation of PCA was depicted using the ggfortify v0.4.19 R package, with 95% confidence ellipses plotted around each population. After conducting principal component analysis, the population structure was determined using the ADMIXTURE v1.3.0 program [27]. Evaluated ancestral populations for K values ranging from 1 to 10, and the optimal number of ancestral populations was determined using the cross-validation criterion. ADMIXTURE v1.3.0 was run with 10-fold cross-validation and the default random seed. Cross-validation was enabled by simply adding the --cv flag to the ADMIXTURE command line. A good value of K will exhibit a low cross-validation error compared to other K values. Additionally, genetic distances were calculated by PLINK V1.9, with --distance 1-IBS flat-missing square to generate a pairwise identity-by-state (IBS) distance matrix. A heatmap was generated using the pheatmap v1.0.13 package in the R v4.5.0 software with Euclidean distance metric and Ward.D2 clustering method [28]. The Neighbor-Joining (NJ) tree was constructed from the IBS distance matrix using R, visualized, and enhanced with the iTOL website [29].

### Genetic diversity parameters

To assess genetic diversity within and between two sheep populations, calculated several metrics: nucleotide diversity (π), inbreeding coefficient (F_IS_), observed heterozygosity (H_O_), expected heterozygosity (H_E_), minor allele frequency (MAF), and average pairwise genetic distance (D). The π was determined using VCFtools v0.1.17 with a 100-kb sliding window and a step size of 50 kb across the genome. For the calculations of MAF, H_O_, H_E_, and F_IS_, PLINK v1.9 utilised [26]. Additionally, the average proportion of alleles shared between two individuals within the same breed (DST) using PLINK v1.9, from which the genetic distance between individuals in a sheep population was calculated as D = 1 – DST [30].

### Effective population size and linkage disequilibrium decay

The effective population size (Ne) was estimated using SNeP v1.1 [31] through the analysis of linkage disequilibrium (LD) patterns between SNPs. To exclude low-frequency alleles, a MAF threshold of 0.05 was applied [32]. Ne trajectories were obtained using the default LD-based settings of SNeP (mindist = 50 kb, maxdist = 4 Mb, alpha = 1, recombination rate 1 × 10 ⁻ ⁸ per bp, sample-size–corrected r² and unphased genotypes), following Barbato et al. (2015) [31], and 95% confidence intervals were derived from a block-bootstrap over LD bins (weighted by the number of SNP pairs per bin). Linkage disequilibrium (r²) among SNPs was estimated separately within each sheep population using PLINK v1.9 with parameters --ld-window-r^2^: 0, ld-window: 100, and ld-window-kb: 1000 [26]. Pairwise r² values were grouped into consecutive 10‑kb non-overlapping physical distance bins from 0 to 1 Mb (0–10 kb, 10–20 kb, …, 990–1000 kb). For each distance bin, we calculated the mean r², its standard error, and the corresponding 95% confidence interval across all SNP pairs; the mean r² values were plotted against the midpoint of each distance bin, with shaded 95% confidence ribbons, to visualize LD decay patterns in each population [33].

### Detection of selection signatures

In this study, F_ST_ and XP-EHH statistical methods were used to identify selection signatures in IB and AB samples and to examine the demographic differences. Two populations were first merged based on common SNPs. F_ST_ identified genomic regions selected differentially in IB and AB. The F_ST_ statistic was calculated using PLINK v1.9 software, based on the unbiased estimator suggested by Weir and Cockerham (1984). Considering population differences and sampling error, this method has an advantage [34]. After calculating F_ST_ value, the average numerical values of the five adjacent SNPs were used as the Win5F_ST_ value to increase the chance of identifying selection signatures instead of the numerical value of each SNP [35]. The Win5F_ST_ approach indicates overlap windows by 5 adjacent SNPs rather than a fixed BP window, with one SNP moving forward in the sequence. R v.4.5.0 software was used to calculate numerical values for a high percentage of each chromosome, and regions with high values for all adjacent SNPs were considered as selection signatures [36]. XP-EHH is based on linkage disequilibrium and haplotype length. In this method, selection signatures were identified by alleles with high EHH [15]. Furthermore, XP-EHH compares EHH integrals between two populations with the same number of SNPs, taking into account linkage disequilibrium, haplotype length, as well as the frequency and distance between SNPs. The rehh v3.2.2 R package was used to calculate XP-EHH [37]. Before XP-EHH analysis, genomic VCF files for each population were filtered for high-quality SNPs (MAF > 0.01) and confirmed to contain phased haplotypes [14]. No additional imputation or phasing was performed, as the input VCFs were derived from pre-phased sequencing data. For each chromosome, haplotypes were converted to haplohh objects using data2haplohh () from rehh v3.2.2 with: min_maf = 0.01, allele_coding = “01”, polarize_vcf = FALSE, remove_multiple_markers = TRUE [37]. XP-EHH is based on linkage disequilibrium and haplotype length, identifying selection signatures by comparing EHH integrals between populations [15]. EHH scans were performed using scan_hh() (limhaplo = 5, limehh = 0.05, limehhs = 0.05, phased = TRUE) and XP-EHH scores calculated per-chromosome with ies2xpehh(standardize = TRUE) which performs Z-score normalization within each chromosome (mean = 0, SD = 1) [37]. Scans were conducted per chromosome and combined genome-wide. The top 1% outliers in F_ST_ and XP-EHH values (|XP-EHH| ≥ 3) were selected following standard empirical thresholds without multiple testing correction due to marker LD and the exploratory nature of selection scans [38–40]. Finally, after identifying selection signatures using the F_ST_ and XP-EHH methods, related putative candidate genes were extracted with PLINK V1.9, utilizing a gene list generated by the Illumina Company [41].

### Gene ontology and QTL report

DAVID (version 6.8) was used to detect important metabolic KEGG pathways and gene ontology analysis. DAVID is a database for visualisation, annotation, and integrated discovery [42]. After applying Bonferroni correction for multiple testing, pathways with p-values less than 0.05 were considered statistically significant. The AnimalQTL database (www.animalgenome.org/cgi-bin/QTLdb/OA/index) was used to identify positions of previously reported sheep QTLs [21]. Then, the identified genes from important F_ST_ and XP-EHH regions were matched with those QTLs to find any traits under selection in Baluchi sheep from Iran and Afghanistan.

All analyses were performed on a Linux (Ubuntu 20.04) high-performance computing cluster using R v4.5.0, plink v1.9, and VCFtools v0.1.17. Full command flags and random seed details are provided in the S1 File.

## Results

### Quality control of data and population structure

**Fig 1:**
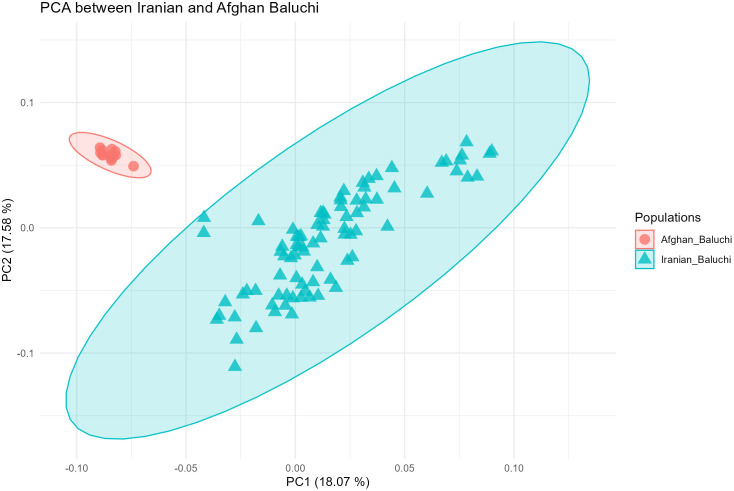
Population structures analysis. Results of Principal component analysis of Iranian (blue, n = 86) and Afghan Baluchi (red, n = 15) sheep based on SNPs. Eigenvector x (x-axis is PC1) versus Eigenvector y (y-axis is PC2). PC1 and PC2 account for 18.07% and 17.58% of the total variance, respectively. 95% confidence ellipses shown per population. SNPs, single-nucleotide polymorphisms.

After quality control, 44791 SNPs and 42305 SNPs remained in AB and IB, respectively. Quality control levels are shown in S1 Table. Additionally, we utilized shared SNPs between the two populations to estimate genetic diversity, population structure, FST, and XP-EHH (38193 SNPs), thereby obtaining more accurate results. In general, IB and AB populations most likely have originated from a common founder population. However, the PCA result indicated that the IB and AB populations were distinct (Fig 1). The results of the principal component analysis of the IB and AB populations indicate that PC1 and PC2 account for 18.07% and 17.58% of the total variance, respectively.

**Fig 2:**
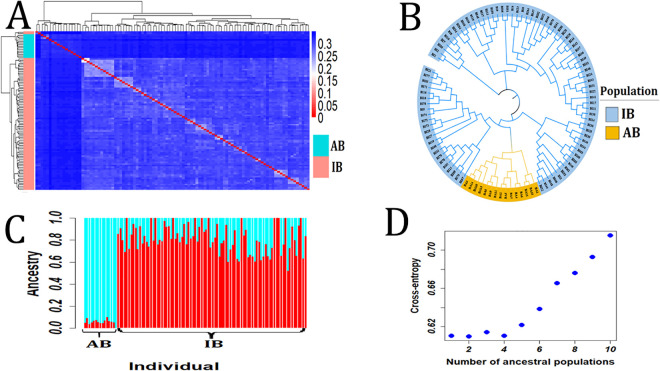
Genetic relationships and population structure among AB (Afghan Baluchi) and IB (Iranian Baluchi) populations. (A) Heatmap of pairwise 1-IBS genetic distances between the AB and IB populations. Euclidean distance; Ward.D2 hierarchical clustering. red (low similarity) to blue (high similarity). (B) Neighbour-joining tree based on 1-IBS genetic distances of IB and AB sheep. (C) ADMIXTURE analysis results for K = 2 displaying ancestry proportions for individuals in the AB (individual 1-15) and IB populations (individual 16-101). (D) Cross-entropy error values from the ADMIXTURE analysis suggest that K = 2 is the best number of genetic clusters‌‌.

A heatmap was generated to visualize pairwise genetic distances among 101 individuals from the AB and IB sheep populations. The IB individuals formed a tight genetic cluster characterized by predominantly low pairwise distances (pale blue), indicating notable genetic uniformity within this population. In contrast, the AB population displayed substantially higher genetic distances (deep blue), highlighting the higher genetic distinctness between individuals in this population. The diagonal red band of low distances corresponds to self-comparisons, while the off-diagonal patterns reveal the hierarchical genetic structure differentiating the two populations (Fig 2A). The NJ tree analysis revealed that although both populations had a common ancestor in the relatively distant past, in recent times the genome of AB population had become different from that of IB population (Fig 2B). Also, ADMIXTURE analysis was conducted to investigate the population structure (Fig 2C), evaluating K values from 1 to 10. The optimal number of genetic clusters was determined to be K = 2, based on the lowest cross-entropy error of 0.603 observed during cross-validation (Fig 2D). At K = 2, a clear genetic distinction was evident between the AB and IB populations. Higher K values, such as K = 3 and K = 4, revealed further sub-structuring within the populations, with unique ancestry components identified in several individuals, as shown in S2 Fig.

### Genetic diversity parameters

Genetic diversity parameters, including MAF, nucleotide diversity (π), H_O_, H_E_, D, and F_IS_, were calculated for AB and IB populations (Table 1). The MAF values were nearly identical across both populations, with AB having a mean of 0.291 and IB slightly lower at 0.283. Nucleotide diversity showed minimal variation, ranging from 0.000037 in IB to 0.000039 in AB. H_O_ and H_E_ values were similar between the two populations, with AB showing both metrics at 0.38, while IB had H_O_ at 0.38 and H_E_ at 0.37. The IB population had the lowest H_O_ and H_E_. The F_IS_ values were low in both populations, with AB at −0.0177 and IB at −0.0194, indicating lower deviation from the Hardy-Weinberg equilibrium. The D values were also slightly lower in the IB population (0.296) than in the AB population (0.315).

**Table 1:** Different parameters of genetic diversity in AB and IB populations.

Parameter	IB	AB
π*	3.782	3.988
*F*_*IS*_	−0.019	−0.018
*H*_*O*_	0.38	0.384
*H*_*E*_	0.372	0.381
*MAF*	0.283	0.291
*D*	0.296	0.315

*π*: nucleotide diversity, *F*_*IS*_: inbreeding coefficient, *H*_*O*_: observed heterozygosity, *H*_*E*_: expected heterozygosity, *MAF*: minor allele frequency, *D*: average pairwise genetic distance, *Values expressed as 10^−5^.

### Effective population size and linkage disequilibrium decay

**Fig 3:**
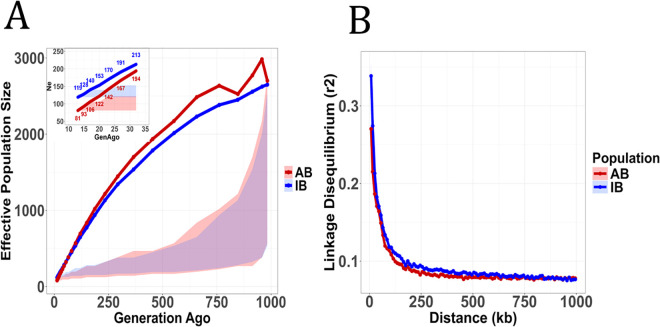
Effective population size (Ne) and linkage disequilibrium (LD) decay for the AB and IB populations. (A) Changes in effective population size (Ne) over the last 1000 generations based on genome‑wide LD (r²) binned by physical distance. Shaded areas represent 95% confidence intervals obtained by block bootstrap over LD bins. (B) Decline in LD, shown as r², with increasing distances between SNPs. Shaded areas represent 95% confidence intervals of mean r² based on the standard error within each bin.

The effective population size (Ne) over the past 1000 generations was estimated using LD-based methods, revealing a steady decline in Ne across both populations, indicative of diminishing genetic diversity over time. Between the two populations, IB showed higher Ne values in recent generations compared to AB. Approximately 400 years ago, Ne was estimated at around 1,700 for AB and 1,537 for IB, with AB initially having the larger Ne. However, this pattern shifted in more recent generations, with IB maintaining the higher Ne, suggesting differing historical population dynamics between the two populations. Shaded 95% confidence intervals around the Ne trajectories, obtained by block bootstrap over LD bins, illustrate the uncertainty associated with these recent Ne estimates (Fig 3A). Linkage disequilibrium (LD) was evaluated for AB and IB populations by calculating pairwise r² values over SNP distances up to 1 Mb. Both populations exhibited a decreasing trend in average LD (r²) with increasing physical distance between SNPs. Shaded 95% confidence ribbons around the mean r² curves illustrate the uncertainty of these estimates across distance bins. Notably, AB displayed the most rapid LD decay, consistently showing lower r² values across all distances, indicative of reduced genetic linkage within this population (Fig 3B); the number of pairwise SNP comparisons contributing to each distance bin is provided in S2 Table.

### Detection of selection signatures

**Fig 4:**
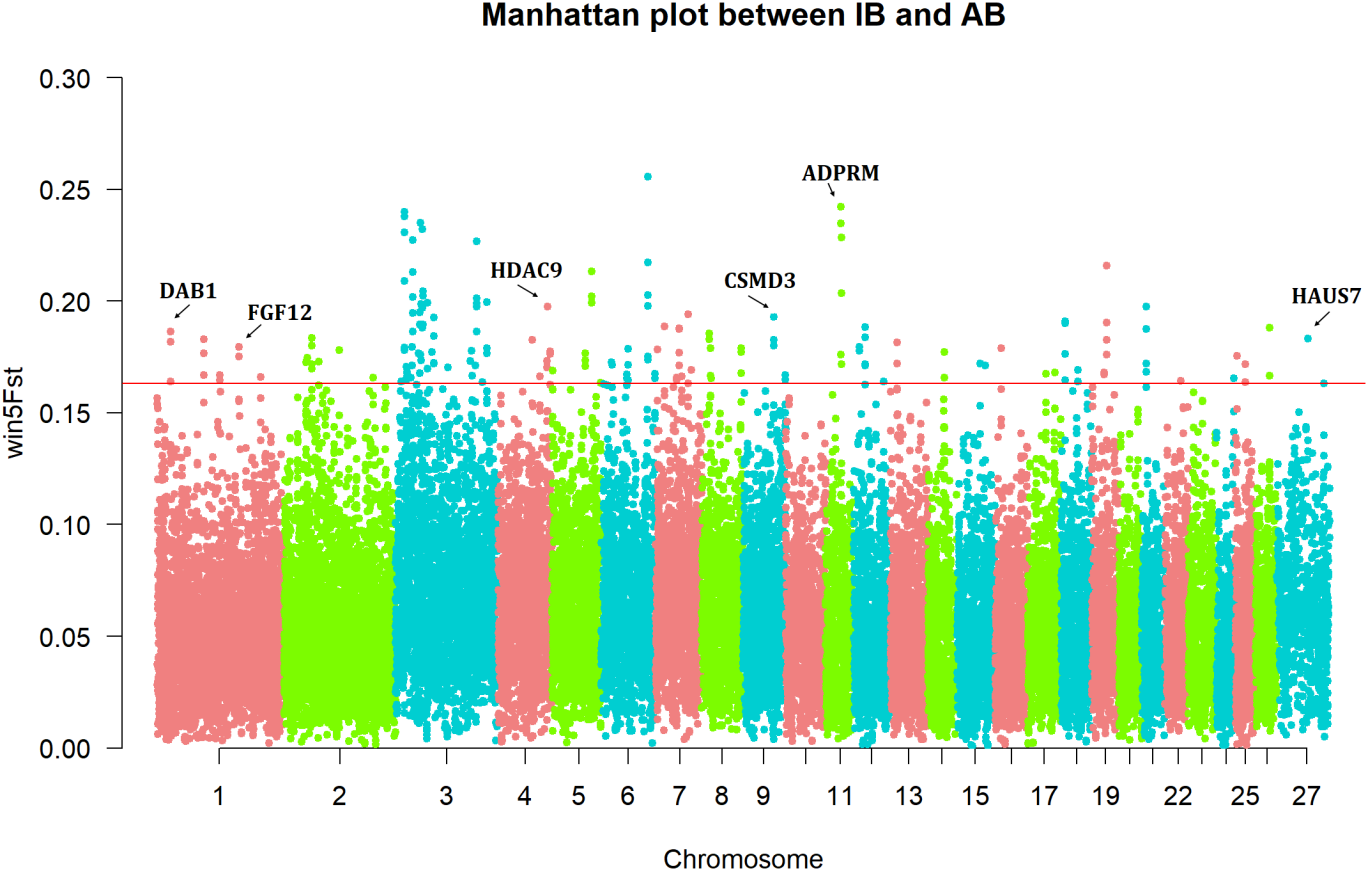
Distribution of win5F_ST_ values in the genome between Iranian and Afghan Baluchi sheep. X-axis represents the chromosome and Y-axis represents the distribution of Win5F_ST_. Chromosomes are separated based on color. In this graph, regions with win5F_ST_ values above the line (0.18 < Win5F_ST_) are introduced as significant regions, which are spread over 21 different chromosomes.

In this study, the results of F_ST_ showed that several regions under selection were located on different chromosomes. A Manhattan plot of F_ST_ statistics is shown in Fig 4. In this plot, regions with high win5F_ST_ values represent the distinction between IB and AB. The complete information is presented in S3 Table.

**Fig 5:**
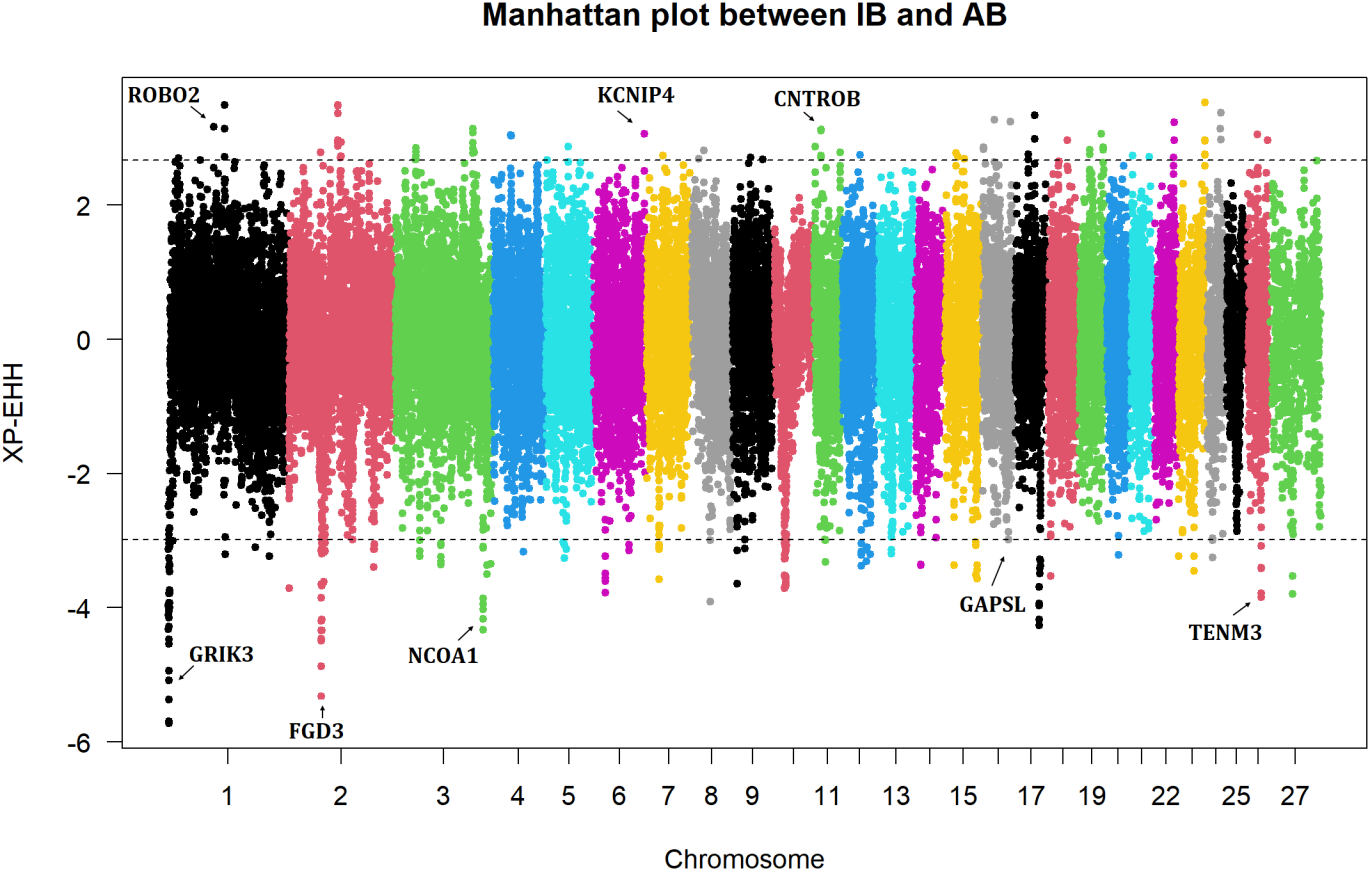
The distribution of XP-EHH values in the genome between Iranian and Afghan Baluchi sheep showed negative values related to IB and positive values related to AB. Chromosomes are separated based on color. X-axis represents chromosome, and Y-axis represents XP-EHH values. Significant regions in the IB population are markers whose XP-EHH distribution is less than −3, and significant regions in the AB population are markers whose distribution is higher than 3.

XP-EHH was performed to evaluate the genomic pattern of positive selection in the Baluchi populations. In this study, XP-EHH was used as a complement to F_ST_, with more reliable results in case of identification of similar genomic regions. The 38193 SNPs utilised in the current study covered 2869.91 Mb of the sheep genome, with an average distance of 77.13 kb between neighbouring SNPs. The Manhattan plot was drawn to identify selection signatures in IB and AB (Fig 5). According to this plot, 87 significant XP-EHH signals were detected in IB sheep, whereas only 27 were found in AB sheep.

### Gene ontology and KEGG pathway analysis

The Gene Ontology analysis showed that significant genes in terms of F_ST_ were enriched in female pregnancy and regulation of glutamate receptor activity. KEGG pathway analysis and Gene Ontology of XP-EHH in IB population putative candidate genes were enriched in pathways, including intracellular signal transduction and regulation of microtubule cytoskeleton organization. In AB population, putative candidate genes were enriched in pathways including cancer and MAPK signalling pathway, and neuromuscular junction development (Table 2).

**Table 2:** KEGG pathway and Gene Ontology analysis of F_ST_ and XP-EHH putative candidate genes were related to Iranian and Afghan Baluchi sheep.

Category	Term	Bonferroni-corrected P-Value	Genes
F_ST_			
BP^1^	regulation of N-methyl-D-aspartate selective glutamate receptor activity	0.014	RASGRF1, CRH
BP	female pregnancy	0.028	LOC443348, CRH
XP-EHH IB sheep			
BP	regulation of microtubule cytoskeleton organization	0.029	TRAF3IP1, BICD2
BP	intracellular signal transduction	0.038	SYK, TRAF3IP1, RGS6
CC^2^	ciliary base	0.025	PRKAR2B, TRAF3IP1
XP-EHH AB sheep			
BP	axon guidance	0.001	ROBO2, RELN, SEMA3C, CHL1, DVL1, CNTN4
BP	nitrogen compound metabolic process	0.030	MYO1E, ARID5B
BP	neuromuscular junction development	0.050	COL4A5, CACNA2D2
KEGG	Dilated cardiomyopathy	0.008	CACNA2D3, CACNA2D2, ADCY2
KEGG	Adrenergic signaling in cardiomyocytes	0.023	CACNA2D3, CACNA2D2, ADCY2
KEGG	Oxytocin signaling pathway	0.025	CACNA2D3, CACNA2D2, ADCY2
KEGG	Pathways in cancer	0.026	DVL1, COL4A5, ADCY2, FGF11
KEGG	MAPK signaling pathway	0.050	CACNA2D3, CACNA2D2, FGF11

1: Biological Process, 2: Cellular Component.

### Finding QTLs

Significant genes of F_ST_ were associated with some important traits in sheep, including body weight and hot carcass weight. Moreover, XP-EHH putative candidate genes in IB population were related to muscle weight in carcass, staple length, and milk fat percentage. Also, XP-EHH putative candidate genes in AB population were related to body weight, hot carcass weight, and milk yield traits, which are important in sheep (Table 3). The complete list of significant genes related to these traits has been presented in S4–S6 Tables (S4 Table).

**Table 3:** Significant genes of F_ST_ and XP-EHH were associated with some QTL in sheep.

Chr	Gene	QTL
F_ST_		
1	DAB1	FATP/LMYP/MUSWT
1	FGF12	ASREP/BONE_WT/FATP/REPS
4	HDAC9	BW/HFEC/CVFD_PRI
9	CSMD3	HCWT/LMA/MUSWT
11	PCDH9	BONEP/FATP/FATWT/FECGEN/HO/LMYP/TESTWT
25	CTNNA3	CVFD_PRI/MFDIAM/SL/TESTWT
XP-EHH IB sheep		
1	GRIK3	LMYP/MUSWT
2	FGD3, SYK	FA-C20:4/FA-C22:5/FA-C20:5/FA-C18:3/MFPER
3	NCOA1	HFEC/SL/TFEC_1
8	MTHFD1L	FECGEN/INTFAT/LATRICH_2
26	KCNU1	MUSWT/Stature/UDDATT/WORMCT
26	TENM3	MUSWT
XP-EHH AB sheep		
1	ROBO2	BONE_WT/BONEP/FATP/LMYP/FA-C22:5/FA-C20:5/PUFA/MUSWT
6	KCNIP4	BW/FATP/FATWT/FECGEN/HCWT/LMYP/MFDIAM/MUSWT
7	MYO1E	HFEC/LMA/PP/CVFD_PRI/SL
8	FILIP1	INTFAT/LATRICH_2
11	CNTROB, FGF11	BW/HCWT/INTFAT/MPUFA/PY/MYPERS/MY/LATRICH_2
16	ADCY2	DRESSING/LMYP/SCFT
19	CACNA2D2, CHL1, CNTN4	DRESSING
25	ARID5B, USP54	MFDIAM/CVFD_PRI/SL/TESTWT/UYC

AMDG; Age at maximum daily gain, ADG; Average daily gain, BFLUMB3; Backfat at third lumbar, BW; Body weight, BDENS; bone density, BONE_WT; Bone weight in carcass, BONEP; Carcass bone percentage, FATP; Carcass fat percentage, DRESSING; Dressing percentage, FATWT; fat weight in carcass, FECGEN; Faecal egg count, FCURV; Fibre curvature, FLYD; Fleece yield, HFEC; Haemonchus contortus FEC, HO; Horns, HCWT; Hot carcass weight, IGA; Immunoglobulin A level, IGG; Immunoglobulin G level, IOA; Inherited Ovine Arthrogryposis, INTFAT; internal fat amount, JAWL; Jaw length, LMYP; Lean meat yield percentage, LMA; Longissimus muscle area, MFDIAM; Mean fibre diameter, FA-C20:4; Meat arachidonic acid content, FA-C18:1; Meat cis-vaccenic acid content, FA-C22:5; Meat docosapentaenoic acid content, FA-C20:5; Meat eicosapentaenoic acid content, FA-C20:1; Meat gadoleic acid content, FA-C18:2; Meat linoleic acid content, FA-C18:3; Meat linolenic acid content, FA-C14:0; Meat myristic acid content, FA-C18:1; Meat oleic acid content, FA-C16:0; Meat palmitic acid content, FA-C16:1; Meat palmitoleic acid content, PUFA; Meat polyunsaturated fatty acid content, FA-C14:0; Meat stearic acid content, MCARPL; Metacarpal length, MFPER; Milk fat percentage, MFY_180D; Milk fat yield, MLACT; milk lactose yield, MPUFA; milk polyunsaturated fatty acid content, PP; Milk protein percentage, PY; Milk protein yield, MYPERS; Milk yield persistency, MY; Milk Yield, MDLUMB3; Muscle depth at third lumbar, MUSWT; muscle weight in carcass, NFEC; Nematodirus FEC, CVFD_PRI; Primary fibre diameter coefficient of variance, RLEGS; Rear leg set, ASREP; Reproductive seasonality, SAOS; Salmonella abortusovis susceptibility, SCS; Somatic Cell Score, SL; Staple length, Stature; Stature, SCFA; Subcutaneous fat area, SCFT; Subcutaneous fat thickness, TESTWT; Testes weight, TOTBONE; Total bone, LATRICH_2; Trichostrongylus adult and larva count, TFEC_1; Trichostrongylus colubriformis FEC, UDDATT; udder attachment, UYC; Useful yield content, WORMCT; Worm count.

## Discussion

The study of genetic diversity between and within animal populations offers valuable insights into the structure and relationships of populations. This knowledge is crucial for the conservation of these populations, enabling them to withstand future environmental challenges and respond effectively to long-term selection, whether natural or artificial, for traits that are economically and culturally significant [10]. Indigenous breeds hold significant cultural value in every country, but their lower productivity often leads to their replacement by commercial breeds. To prevent losses in productivity and to enhance the economics of farming, urgent measures are needed to ensure the survival and protection of these indigenous breeds. Consequently, the genetic structure of indigenous sheep may be impacted by demographic events, such as gene flow between different breeds and subsequent genetic mixing among them [43]. Additionally, identifying genes associated with important economic traits is vital for developing effective breeding programs for both valuable animal species and genetic resources. The main objective of this study was to characterize the genetic diversity, population structure, and detect signals of selection in the indigenous AB and IB sheep populations using 50K beadchip genotyping data.

In this study, the Iranian Baluchi sheep population was sampled using 86 heads from a high-purity flock at a breeding station, a flock considered the most reliable source for pure genetic representation of the breed due to the lack of documented information on the purity of scattered populations in eastern and southern Iran. In contrast, only 15 Afghan Baluchi sheep were sampled from traditional and scattered flocks in various areas of Herat Province, reflecting the serious challenge of identifying and collecting more high-purity animals in this country. This imbalance in sample size, especially for the Afghan population, can lead to increased variance in allele frequency (AB) estimates, reduced statistical power in ADMIXTURE ancestry proportions, F_ST_ pairwise differentiation, XP-EHH selection scans, and a higher risk of false negatives in detecting population differentiation and selection signals. Therefore, the findings related to AB are more exploratory and should be interpreted with caution. To mitigate these limitations, we applied empirical genome-wide thresholds (top 1% outliers) rather than stringent p-value cutoffs and cross-validated results across complementary methods (F_ST_ + XP-EHH + LD decay). Despite these limitations, the combination of using a purebred management herd in Iran and selected field samples from Afghanistan provides a valuable picture of genetic differences and similarities between these two management and geographical contexts and can serve as a basis for designing larger studies in the future.

Analysis of population structure and genetic differentiation, including PCA, Admixture, and NJ tree, revealed a relatively high level of genetic differentiation between the AB and IB sheep populations, despite their common ancestry. This significant genetic divergence may be attributed to differing selection goals, adaptations to their respective environments, and distinct breeding practices for these two populations. A study conducted in 2020 by Eydivand et al. examined 14 native sheep breeds from the Middle East and South Asia. The findings revealed that while Iranian and Afghan Baluchi sheep are more similar to each other than to other breeds and descended from a common lineage, they are classified into two distinct populations [5]. Also, in a study conducted by Barani et al. (2023) that compared three Iranian sheep breeds, the results indicated that Baluchi sheep are genetically distinct from the other breeds. This distinction is largely due to the breeding practices at the Breeding Center and the isolation of this population [44]. A study conducted on three Afghan sheep breeds revealed that the Baluchi breed is genetically close to the other breeds but is classified in its own separate group. This distinction can be attributed to the traditional breeding practices associated with the Baluchi breed and the traditional interbreeding within this breed population [24].

Genetic diversity can be examined using various parameters in studies. In this study, genetic diversity between the two populations, AB and IB, revealed that various parameters, such as H_O_, H_E_, MAF, π, D, F_IS,_ and LD, indicate that the genetic diversity in the IB population is quite similar to, but slightly lower than, that of the AB population. But in terms of effective population size (Ne), the IB population has fared better in recent generations compared to the AB population. The observed pattern of lower genetic diversity but higher recent Ne in IB compared to AB can be explained by three interacting factors. First, the IB samples from Abbas Abad Breeding Centre represent a managed flock with systematic mating strategies designed to maintain genetic diversity and avoid inbreeding, artificially inflating recent Ne estimates despite potentially lower standing variation compared to more diverse field populations. Second, LD-based Ne is highly sensitive to sample size, with smaller samples producing downward-biased recent Ne estimates due to increased variance in allele frequency sampling and stochastic LD inflation [45,46]. Third, AB samples from traditional Herat flocks likely experienced more severe historical bottlenecks and higher recent relatedness, reducing effective Ne, while the station-managed IB flock benefits from a larger census size and controlled mating. The study by Barani estimated the levels of H_O_, H_E_, and MAF in Iranian Baluchi sheep to be 0.37, 0.38, and 0.28, which aligns with our results [44]. In another study, the average H_O_ statistic was estimated to be between 0.34 and 0.39 for Iranian sheep, including Afshari, Moghani, Qezel, Zel, and Lori-Bakhtiari, and between 0.37 and 0.38 for Afghan sheep, including Baluchi, Gedik, and Arab breeds [5]. In a study conducted by Shi on two Tibetan sheep breeds, the results indicated the following values: for the OuLa breed, H_O_ was 0.226, H_E_ was 0.277, MAF was 0.223, and π was 0.0027. For the PanOu breed, the values were 0.221 for H_O_, 0.267 for H_E_, 0.228 for MAF, and 0.0026 for π [8]. In a study of Chinese breed sheep conducted by Cheng, H_E_ ranged from 0.226–0.316, while H_O_ varied from 0.238–0.240 [47]. A study by Demissie et al. (2025) on various sheep breeds revealed that the average values of genetic diversity parameters, including H_O_, H_E_, F_IS_, and MAF, were 0.352, 0.344, −0.023, and 0.261, respectively [48]. In a study conducted on nine goat breeds, the results showed that the H_O_ was 0.374 ± 0.021, H_E_ was 0.0369 ± 0.023, and D ranged from 0.263 to 0.332 [49]. In a study examining genetic diversity among three goat breeds, the average values for the parameters MAF, H_O_, H_E_, π, and F_IS_ were found to be 0.32, 0.304, 0.306, 0.28, and 0.009, respectively [30]. The results of the linkage disequilibrium (LD) study indicated that the LD rate decreases as the distance between markers increases, which aligns with findings from previous studies conducted on different sheep breeds [10,30,50]. Additionally, the average LD rate was higher in the IB population compared to the AB population. A higher LD rate is associated with lower genetic diversity [33]. The results regarding effective population size (NE) indicate a significant decrease in this parameter in new generations compared to earlier ones [46,51]. The primary factors contributing to this decline include selection pressure, climate change, artificial selection, flawed mating programs, and the use of a limited number of superior rams. Specifically, in the Iranian Baluchi, the effective population size has dropped from 2,840 over 1000 previous generations to just 119 over the last 13 generations. Similarly, in the Afghan Baluchi sheep, the effective population size has decreased from 2,890 over 680 generations to only 81 in the past 13 generations. The NE results are consistent with findings from previous studies conducted on different racial groups [9,52,53].

The identified selection signatures represent candidate regions based on empirical top 1% (F_ST_) and |XP-EHH| ≥ 3 thresholds, which are heuristic cutoffs commonly used in livestock [54–56] genomic scans but likely include false positives, particularly given the limited AB sample size. Functional validation through targeted sequencing or association studies will be required to confirm these preliminary signals. The selection signature aims to identify areas of genome differentiation between populations based on SNP information. This method is more effective when working on related populations [18]. Therefore, we analysed two populations of Iranian and Afghan Baluchi sheep that have common ancestors [5]. In this study, selection signatures were studied using F_ST_ and XP-EHH statistics. The results showed that several regions of the genomes of these two populations were selected, whose genes were associated with important economic traits. The selection signature was more intense in Iranian Baluchi sheep because they were from an animal breeding center. Therefore, it could be concluded that breeding programs caused significant differences in both studied populations at the genome level.

Regions under selection found by F_ST_ statistics showed that *HDAC9*, *CSMD3*, *DAB1*, *FGF12,* and *PCDH9* genes were enriched in body weight, hot carcass weight, muscle weight in carcass, reproductive seasonality, and carcass fat percentage traits, respectively. The *HDAC9* gene was involved in the muscle structure development pathway in the study of Cheng et al. (2020), which was performed on Chaka sheep using whole genome sequencing [47]. Moreover, this gene is related to muscle in Garut sheep breed from Indonesia, which is used in ram fights [57]. Additionally, *HDAC9* inhibited muscle differentiation transcriptional circuitry through its negative-feedback loop by suppressing MEF2 activity [58]. Several studies performed on livestock showed that *HDAC9* gene is associated with skeletal muscle development [59], carcass, and meat traits [60]. In a study of 14 indigenous sheep breeds from the Middle East and South Asia, the results showed that *HDAC9* gene is related to economic traits and milk traits [5]. In another study on South African Merino and Afrino sheep populations, *HDAC9* gene was associated with reproductive traits in the Merino population [61]. Also, *HDAC9* was reported as a selection signature in a study on worldwide sheep populations [57].

In identification of some traits in Assaf and Churra dairy sheep breeds found that the *CSMD3* was related to ILCY (individual laboratory cheese yield) trait in the Churra breed [62]. Also, *CSMD3*, which encodes a transmembrane protein [63], was linked to body size and stature in cattle similar to our finding in sheep [64]. In the current study, *FGF12* was identified as a putative candidate gene relating to reproductive traits. It has been reported in other studies related to the same reproductive traits in cattle and goat [65]. Functional annotation of differentially expressed mRNAs in hair follicle tissue showed that *FGF12* was enriched in MAPK, PI3K-Akt, and RAS signalling pathways, which had a certain influence on hair follicle growth and development [66] and reproductive traits [65]. Gene ontology enrichment analysis in cashmere goats revealed that *FGF12* gene was enriched in several biological pathways that were involved in hair follicle development [67]. Protocadherins are thought to be involved in different aspects of neuronal functions and development. *PCDH9* is involved in synaptic cell adhesion [68]. In a study by Mastrangelo et al. (2019), it was shown that the *PCDH9* gene was associated with the signal of fat deposition pathway in domestic sheep breeds from Africa and Eurasia [69]. In research on local adaptation of Mediterranean sheep and goats, selection signatures involving the *PCDH9* gene, was identified in both species and therefore, could play a significant adaptive role [70]. In a study by Bakhtiarizadeh, *PCDH9* in Zel sheep breed was found to be associated with fat deposition. The *PCDH9* with nine SNPs was located within a sheep QTL region for carcass fat percentage. These findings indicated the role of this gene as an important putative candidate for development of fat-tail in sheep [71]. Our results showed that *CTNNA3* gene is associated with staple length. This gene was significantly related to body weight, height, length, and chest circumference, which can be used as an important marker in improving growth traits in sheep breeding [72]. Multiple studies pointed out that *CTNNA3* gene is important for the formation of a stable complex with the other catenins and cadherins, playing a role in solid cell–cell adhesion [73]. In biological pathways involving adherens junctions and CAMs, *CTNNA3* gene was closely related to cell adhesion mechanisms [74]. In a study by Chen et al. (2021), results showed that *CTNNA3* gene was related to reproduction and production traits [75].

Our QTL results based on positive XP-EHH, which is related to the Afghan Baluchi sheep, showed that *ROBO2* gene was related to lean meat yield percentage, muscle weight in carcass, and carcass fat percentage and other growth-related traits. Our results were highly consistent with the results of Montiel et al. (2020) study performed on Pelibuey Sheep by Genome-Wide Association Study; Also, *ROBO2* gene was related to litter size [76]. During the development of the central nervous system (CNS), this gene is crucial for axon guidance across the midline [77]. In this study, the relationship between this gene and axon guidance was identified, too. Moreover, *ROBO2* gene is crucial during the early stages of follicle development in sheep, as well as during ovary development as a factor determining follicle maturation [78], resulting in an altered expression pattern that may be affected by additional factors in the ovary and steroid hormones in other reproductive tissues [79]. In a study by Mohammadi et al. *ROBO2* was found to be associated with milk fat and protein yield [80]. Also, according to previous studies, *ROBO2* was associated with fat metabolism, particularly in the fatty acid profile [81]. According to our results, *KCNIP4* gene was associated with body weight, muscle weight in carcass, hot carcass weight, and fat weight in carcass traits in AB population. *KCNIP4* was related to economic traits in genomic scans for selective sweeps in sheep breeds from South Asia and Middle East. This gene plays a critical role in heart health, as well as skeletal muscle development, body weight, and immune response [5]. A genome-wide association study of Baluchi sheep showed that *KCNIP4* is also directly involved in muscle growth and fat storage in addition to indirectly modulating temperature [82]. Furthermore, *KCNIP4* was identified as a gene that was related to body weight and hot carcass weight traits in a genome-wide association study in Zandi sheep, which is in agreement with the results of the current study [83]. *WWOX* gene was also reported as a putative candidate gene associated with bone weight in carcass and total bone traits in AB population in this study. According to Mohammadi et al. (2020), the *WWOX* gene was associated with the regulation of postnatal growth, skeletal muscle differentiation, and bone growth in sheep [83]. The *FGF11* gene was also reported as a putative candidate gene that was associated with body weight, hot carcass weight, and milk yield traits in this study. This gene was related to body size in Chinese Merino [84], and it was reported as a selection signature in Swiss sheep breeds [85]. Moreover, in some sheep genetic resistance to gastrointestinal helminth infection results show that *FGF11* gene being related to organ development [86]. Another identified gene is the *CHL1* gene, which is related to axon guidance. A study conducted on Tibetan sheep; results showed that *CHL1* gene is related to gas transport. Also, *CHL1* is associated with homeostatic adaptation during hypoxia, in comparison to wild-type individuals; *CHL1* augmented ventilatory responses were also recorded in Tibetan breed of sheep [87] and Tibetans [88] at high altitudes.

Results of QTL based on negative XP-EHH relating to the Iranian Baluchi sheep showed that *FGD3* gene is related to the Milk fat percentage trait. Moreover, *FGD3* gene, related to metabolism and growth, was found under selection in the Welsh sheep breed [89]. In this study, *NCOA1* is another significant gene relating to the staple length trait. To our knowledge, *NCOA1* was related to litter size in Hu and Small-tailed Han sheep and Icelandic sheep [90]. The other significant gene, *GRIK3* gene, was identified in the IB population, which is associated with lean meat yield percentage and muscle weight in carcass traits in this study. This gene encodes a glutamate inotropic receptor which plays a role in multiple biological pathways and can be found in diverse animal species, including cattle, pigs, chickens, and horses. Research has shown that *GRIK3* plays a role in nervous system processes, membrane potential regulation, synaptic transmissions, and also has an essential role in the glutamate receptor signalling pathway. Moreover, *GRIK3* was related to reproduction and fertility traits in a genome-wide association study in South African sheep breed [91].

It should be noted that the genes highlighted here are putative candidates, and that our interpretations are largely based on positional overlap with previously reported QTL and association signals. Because many livestock QTL are broad and sometimes pleiotropic, QTL overlap alone cannot prove causality, and some of the reported functional links may not be directly relevant to the specific environmental and management context of Baluchi sheep.

## Conclusion

This study analysed the population structure, genetic diversity, and selection signatures between Iranian and Afghan Baluchi sheep populations. Although the Baluchi sheep population of Iran and Afghanistan shared a common ancestor, the population structure results indicated that the two populations are genetically distinct from one another. This difference may be attributed to varying selection goals, geographical factors, and environmental adaptations, and/or genetic drift due to demographic history. Overall, moderate genetic diversity was observed in both the Afghan Baluchi (AB) and Iranian Baluchi (IB) sheep populations. However, the IB population exhibited the lowest level of genetic diversity and the highest rate of linkage disequilibrium decay, while showing a better condition in terms of effective population size. Putative candidate genes identified in putative selection regions are associated with reproduction, milk production, and growth traits, consistent with previous studies. However, some signals may reflect genetic drift or demographic history rather than adaptive selection, particularly given the small Afghan sample size (n = 15). These gene-trait associations should be interpreted cautiously and framed as hypotheses requiring functional validation. According to the results of this research, it can be stated that the traditional selections (phenotypic) and modern breeding programs for economically important traits such as growth, reproduction, immune system, etc., might be in the same direction. These results should be interpreted as reflecting differences between a managed breeding population and a traditional field population rather than national level divergence. Further investigation with a larger sample size is required to compare the performance of these populations to assess the impact of identified selection signatures on economic traits. Utilizing validated QTLs as described in this study could be applied to reveal the direction of breeding plans in other livestock species such as cattle, goats, horses, chickens, and sheep.

## Supporting information

S1 Fig The Baluchi sheep, right: Iranian, left: Afghan. (PDF)

S1 Table Description of quality control steps in Iranian and Afghan Baluchi sheep. (PDF)

S1 File Full command flags and random seed details are provided. (PDF)

S2 Fig ADMIXTURE analysis results for K = 3 and K = 4, for individuals in the AB and IB populations. (PDF)

S2 Table Number of pairwise SNP comparisons contributing to each distance bin. (PDF)

S3 Table Significant selection signatures via WIN5F_ST_ Method. (PDF)

S4 Table Significant genes of F_ST_ were associated with some QTL in sheep. (PDF)

S5 Table Significant genes of positive XP-EHH were associated with some QTL in sheep‌‌. (PDF)

S6 Table Significant genes of negative XP-EHH were associated with some QTL in sheep. (PDF)

S2 File AB. (CSV)

S3 File IB. (CSV)
