# Supplementary figures and images for: Genetic diversity, population structure, and combined detection of selection signatures in Iranian versus Afghan Baluchi sheep
Source: PLoS One. 2026 Jun 17;21(6):e0350262. doi: 10.1371/journal.pone.0350262 (PMC13274857; doi:10.1371/journal.pone.0350262)

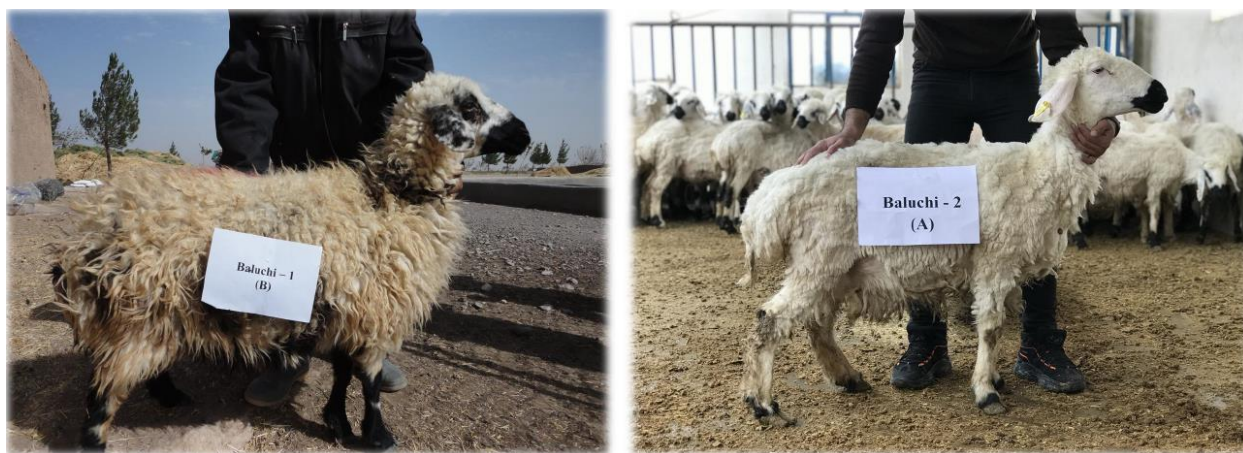

**S1 Fig.** The Baluchi sheep, right: Iranian, left: Afghan.

Supplement: S1 Fig — (PDF) [file pone.0350262.s001.pdf]
