# Supplementary material for: Genetic diversity, population structure, and combined detection of selection signatures in Iranian versus Afghan Baluchi sheep
Source: PLoS One. 2026 Jun 17;21(6):e0350262. doi: 10.1371/journal.pone.0350262 (PMC13274857; doi:10.1371/journal.pone.0350262)
Supplement: S1 Table — (PDF) [file pone.0350262.s002.pdf]

S1 Table. Description of quality control steps in Iranian and Afghan Baluchi sheep.

Quality control	Iranian Baluchi	Afghan Baluchi	Merge two breeds
Total number of animals	86	15	101
Total number of SNPs	51103	46840	48903
Removed animals with GCR< 99%	0	0	0
Removed SNP_S with GCR[1] < 99%	2809	0	10710
Removed SNPs with MAF[2] < 5%	5982	2049	0
Excluded SNPs with HWD[3] < 10^{-6}	7	0	0
IBD (PI_HAT>0.5)	1	0	1
Remained SNPs	42305	44791	38193

1. GCR: Genotype call rate, 2. MAF: Minor allele frequency, 3. HWD: Hardy-Weinberg deviation
