# Supplementary material for: Genetic diversity, population structure, and combined detection of selection signatures in Iranian versus Afghan Baluchi sheep
Source: PLoS One. 2026 Jun 17;21(6):e0350262. doi: 10.1371/journal.pone.0350262 (PMC13274857; doi:10.1371/journal.pone.0350262)
Supplement: S1 File — (PDF) [file pone.0350262.s003.pdf]

S1 File. Full command flags and random seed details are provided.

```
###############QC####################3

setwd("")

system("plink --file BI --maf 0.05 --geno 0.01 --mind 0.01 --hwe 1e-6 --nonfounders --sheep --recode --out BI.QC")

system("plink --file BI.QC --genome --min 0.05 --max 0.5 --sheep --out BI.QC")

system("plink --file B --maf 0.05 --geno 0.01 --mind 0.01 --hwe 1e-6 --nonfounders --sheep --recode --out BA.QC")

system("plink --file BA.QC --genome --min 0.05 --max 0.5 --sheep --out BA.QC")

system("plink --file BI.QC --merge B.QC.ped B.QC.map --maf 0.05 --geno 0.01 --mind 0.01 --hwe 1e-6 --nonfounders --sheep --recode --out BIB.QC")

system("plink --file BIB.QC --sheep --genome --out geno")

system("plink --file BIB.QC --read-genome geno.genome --cluster --sheep --mds-plot 2 --out geno-PCA")

system("plink --file BIB.QC --sheep --distance-matrix --out dataForPCA")

##############Load data for PCA###########3

dist_populations<-read.table("dataForPCA.mdist",header=F)

### Extract breed names

fam <- data.frame(Populations=read.table("dataForPCA.mdist.id")[,1])

### Extract individual names

famInd <- data.frame(IID=read.table("dataForPCA.mdist.id")[,2])
```

```
## Perform PCA using the cmdscale function
# Time intensive step - takes a few minutes with the 4.5K animals
mds_populations <- cmdscale(dist_populations,eig=T,5)

## Extract the eigen vectors
eigenvec_populations <- cbind(fam,famInd,mds_populations$points)

## Proportion of variation captured by each eigen vector
eigen_percent <- round(((mds_populations$eig)/sum(mds_populations$eig))*100,2)



# Visualize PCA in tidyverse
# Load tidyverse
if (!require("tidyverse")) {
  install.packages("tidyverse", dependencies = TRUE)
  library(tidyverse)
}

library(ggfortify )
p<-ggplot(data = eigenvec_populations) +
  geom_point(aes(x = `1`, y = `2`,
                 color = Populations,
                 shape = Populations),
             show.legend = TRUE, alpha = 0.8, size = 4) +
  stat_ellipse(aes(x = `1`, y = `2`,
                   color = Populations,
                   fill  = Populations),
```

```
            geom  = "polygon",
            alpha = 0.2,
            level = 0.999,
            type  = "norm") +
  labs(title = "PCA between Iranian and Afghan Baluchi",
       x = paste0("PC1 (", 18.07, " %)"),
       y = paste0("PC2 (", 17.58, " %)")) +
  theme_minimal()

ggsave("PCA_baluchi.tif",
       plot   = p,
       width  = 9,
       height = 6,
       units  = "in",
       dpi    = 250,
       device = "tiff",
       compression = "lzw")

###########Admixture########
system("plink --file BIB.QC --recode12 --out BIB")
system("plink --file BIB.QC --make-bed --out BIB")

for K in 1 2 3 4 5 6 7 8 9 10; \
do admixture --cv BIB.bed $K | tee log${K}.out; done
grep -h CV log*.out

setwd("")
```

```
project = snmf("genotypes.geno",

K = 1:10,

entropy = TRUE,

repetitions = 10,

project = "new")

project <-read.table("k_means.txt",header=T)

plot(project, col = "blue", pch = 19, cex = 1.2,xlab="Number of ancestral
populations",ylab="Cross-entropy")

savePlot("k","png")


tbl=read.table("BIBA.2.Q")

barplot(t(as.matrix(tbl)), col=rainbow(4),names=1:101,las=2,

xlab="Individual #", ylab="Ancestry", border=NA)


par(mfrow = c(2, 1))

tbl=read.table("BIBA.3.Q")

barplot(t(as.matrix(tbl)), col=rainbow(4),names=1:101,las=3,

xlab="Individual #", ylab="Ancestry", border=NA)


tbl=read.table("BIBA.4.Q")

barplot(t(as.matrix(tbl)), col=rainbow(4),names=1:101,las=3,

xlab="Individual #", ylab="Ancestry", border=NA)


###############heatmap and NJ tree#########


system("plink --bfile BIB.QC --distance 1-ibs flat-missing square --sheep --out dist")
```

```
setwd("")
p<-read.csv("dist.csv",head=T,row.names=1)

library(pheatmap)
population<-c(rep("AB",15),rep("IB",86))
annotation<-data.frame(population=factor(population))
rownames(annotation)<-rownames(p)
pheatmap(p,annotation_row=annotation,
clustering_distance_rows="euclidean",
clustering_distance_cols="euclidean",
clustering_method="ward.D2",
color=colorRampPalette(c("red","white","blue"))(50),
fontsize=12,fontsize_row=8,fontsize_col=8,legend=TRUE,
show_rownames=FALSE,
show_colnames=FALSE,
border_color=NA,annotation_legend=T)

library(ape)
dist_mat<-as.dist("dist.mdist")
dist_mat <- read.csv("dist.csv",row.names=1)
dist_mat2<-as.dist(dist_mat)
phylo_tree <- nj(dist_mat2)
write.tree(phy=phylo_tree, file="tree.newick")

###############genetic diversity ##################

VCFtools --vcf BI.QC.vcf --window-pi 10000 --out BI
```

```
VCFtools --vcf BA.QC.vcf --window-pi 10000 --out BA

VCFtools --vcf BI.QC.vcf --TajimaD 100000 --out BI
VCFtools --vcf BA.QC.vcf --TajimaD 100000 --out BA

system("plink --file BI.QC --hardy --sheep --out BI")
system("plink --file BA.QC --hardy --sheep --out BA")

system("plink --file BI.QC --het --sheep --out BI")
system("plink --file BA.QC --het --sheep --out BA")

system("plink --file BI.QC --freq --sheep --out BI")
system("plink --file BA.QC --freq --sheep --out BA")

#############LD and Ne###############

system("plink --file BI.QC --sheep --r2 gz --ld-window-r2 0 --ld-window-kb 1000 --ld-window
100 --sheep --out LD_BI")
system("plink --file BA.QC --sheep --r2 gz --ld-window-r2 0 --ld-window-kb 1000 --ld-window
100 --sheep --out LD_BA")

setwd("")
library(data.table)
library(dplyr)
library(ggplot2)

make_ld_bins <- function(file, pop, max_kb = 1000, bin_size = 10){
  ld <- fread(file) %>%
```

```
    mutate(dist_kb = (BP_B - BP_A) / 1000) %>%
    filter(dist_kb > 0, dist_kb <= max_kb) %>%
    mutate(bin = cut(dist_kb,
                     breaks = seq(0, max_kb, by = bin_size),
                     include.lowest = TRUE, right = FALSE)) %>%
    group_by(bin) %>%
    summarise(
      mid_kb   = mean(dist_kb),
      mean_r2  = mean(R2, na.rm = TRUE),
      sd_r2    = sd(R2, na.rm = TRUE),
      n_pairs  = n(),
      se_r2    = sd_r2 / sqrt(n_pairs),
      ci_low   = mean_r2 - 1.96 * se_r2,
      ci_high  = mean_r2 + 1.96 * se_r2,
      .groups = "drop"
    ) %>%
    mutate(population = pop)
  ld
}

ld1 <- make_ld_bins("LD_BA.ld.gz", "AB")
ld2 <- make_ld_bins("LD_BI.ld.gz", "IB")

ld_all <- bind_rows(ld1, ld2)

p <- ggplot(ld_all,
            aes(x = mid_kb, y = mean_r2,
```

```
               color = population, fill = population)) +
  geom_ribbon(aes(ymin = ci_low, ymax = ci_high),
              alpha = 0.25, colour = NA) +
  geom_line(size = 1.6) +
  geom_point(size = 2.5) +
  scale_color_manual(values = c("IB" = "blue", "AB" = "red3")) +
  scale_fill_manual(values  = c("IB" = "royalblue1", "AB" = "red1")) +
  labs(x = "Distance (kb)",
       y = "Linkage Disequilibrium (r2)",
       color = "Population",
       fill  = "Population",
       title = "LD decay") +
  theme_bw(base_size = 16) +
  theme(
    axis.title  = element_text(face = "bold", size = 16),
    axis.text   = element_text(face = "bold", size = 12),
    legend.title = element_text(face = "bold", size = 14),
    legend.text  = element_text(face = "bold", size = 12),
    legend.key.width = unit(1.2, "cm")
  )

ggsave("LD_decay_CI.tif",
       plot = p,
       width = 9, height = 7,
       units = "in", dpi = 300,
       device = "tiff", compression = "lzw")
write.csv(ld_all,
```

```
        file = "LD_bins_with_pairs.csv",
        row.names = FALSE)


system("SNeP -ped BI.QC.ped -map BI.QC.map -out IB.QC -chr 1-27
 -mindist 50000 -maxdist 4000000 -maxsnp 100000 -binwidth 50000
 -numbins 30 -expB 4 -recrate 1e-08 -recratemod 0 -threads 1 -minr2 0 -maxr2 1
 -alpha 1 -maf 0.05 -samplesize 0 -itemsThreshold 500 -seed 0 -ldfile 0 -phased 0")

system("SNeP -ped BA.QC.ped -map BA.QC.map -out AB.QC -chr 1-27
 -mindist 50000 -maxdist 4000000 -maxsnp 100000 -binwidth 50000
 -numbins 30 -expB 4 -recrate 1e-08 -recratemod 0 -threads 1 -minr2 0 -maxr2 1
 -alpha 1 -maf 0.05 -samplesize 0 -itemsThreshold 500 -seed 0 -ldfile 0 -phased 0")

setwd("")
library(dplyr)
library(ggplot2)
library(grid)

IB <- read.table("IB.QC.NeAll", header = TRUE)
AB <- read.table("AB.QC.NeAll", header = TRUE)

IB$pop <- "IB"
AB$pop <- "AB"

set.seed(123)
B <- 1000
```

```
boot_ci <- function(dat, B = 1000) {
  dat <- dat %>% arrange(GenAgo)
  n   <- nrow(dat)
  boot_mat <- matrix(NA, nrow = n, ncol = B)

  for (b in 1:B) {
    idx <- sample(1:n,
                  size = n,
                  replace = TRUE,
                  prob = dat$items)
    boot_sample <- dat[idx, ] %>% arrange(GenAgo)
    boot_mat[, b] <- boot_sample$Ne
  }

  ci_low  <- apply(boot_mat, 1, quantile, 0.025, na.rm = TRUE)
  ci_high <- apply(boot_mat, 1, quantile, 0.975, na.rm = TRUE)

  dat %>%
    mutate(ci_low = ci_low,
           ci_high = ci_high)
}

IB_ci <- boot_ci(IB, B)
AB_ci <- boot_ci(AB, B)

plot_df <- rbind(IB_ci, AB_ci)
```

```
xlim_zoom <- c(10, 35)

ylim_zoom <- c(50, 250)


main_plot <- ggplot(plot_df, aes(x = GenAgo, y = Ne,

                                 color = pop, fill = pop)) +

  geom_ribbon(aes(ymin = ci_low, ymax = ci_high),

              alpha = 0.25, colour = NA) +

  geom_line(size = 1.6) +

  geom_point(size = 2.8) +

  scale_x_continuous(limits = c(min(plot_df$GenAgo), max(plot_df$GenAgo))) +

  labs(x = "Generation Ago",

       y = "Effective Population Size",

       color = "", fill = "") +

  scale_color_manual(values = c("IB" = "blue", "AB" = "red3")) +

  scale_fill_manual(values  = c("IB" = "royalblue1", "AB" = "red1")) +

  theme_bw(base_size = 16) +

  theme(

    axis.title  = element_text(face = "bold", size = 16),

    axis.text   = element_text(face = "bold", size = 12),

    legend.title = element_text(face = "bold", size = 14),

    legend.text  = element_text(face = "bold", size = 12),

    legend.key.width = unit(1.2, "cm")

  ) +

  annotate("rect",

           xmin = xlim_zoom[1], xmax = xlim_zoom[2],

           ymin = ylim_zoom[1], ymax = ylim_zoom[2],
```

```
          colour = "black", fill = NA,
          linetype = "dashed", size = 0.8)

zoom_df <- subset(plot_df,
                  GenAgo >= xlim_zoom[1] & GenAgo <= xlim_zoom[2] &
                  Ne     >= ylim_zoom[1] & Ne     <= ylim_zoom[2])

zoom_plot <- ggplot(zoom_df, aes(x = GenAgo, y = Ne,
                                 color = pop, fill = pop)) +
  geom_ribbon(aes(ymin = ci_low, ymax = ci_high),
              alpha = 0.25, colour = NA) +
  geom_line(size = 1.0) +
  geom_point(size = 2.5) +
  geom_text(data = subset(zoom_df, pop == "IB"),
            aes(label = round(Ne)),
            vjust = -1.7,
            size = 3.5, fontface = "bold", color = "blue") +
  geom_text(data = subset(zoom_df, pop == "AB"),
            aes(label = round(Ne)),
            vjust = 2.3,
            size = 3.5, fontface = "bold", color = "red3") +
  scale_x_continuous(limits = xlim_zoom) +
  scale_y_continuous(limits = ylim_zoom) +
  scale_color_manual(values = c("IB" = "blue", "AB" = "red3")) +
  scale_fill_manual(values  = c("IB" = "royalblue1", "AB" = "red1")) +
  labs(x = "GenAgo", y = "Ne") +
  theme_bw(base_size = 10) +
```

```
  theme(
    legend.position = "none",
    axis.title = element_text(face = "bold", size = 10),
    axis.text  = element_text(face = "bold", size = 8),
    plot.margin = margin(2, 2, 2, 2)
  )

combined_plot <- main_plot +
  annotation_custom(
    grob = ggplotGrob(zoom_plot),
    xmin = 0, xmax = 350,
    ymin = 1650, ymax = 3100
  )

combined_plot

ggsave("Ne.tif",
plot=combined_plot,
width=10,
height=7,
units="in",
dpi=300,
device="tiff",
compression="lzw")

###############FST##################
system("plink --file BIB.QC --fst --sheep --out Fst")
```

```
FST<-read.table(file="Fst",skip=1)
winfiveFST<-c()
winfiveTheta<-c()
POS1<-c()
POS2<-c()
POS3<-c()
CHR<-c()
SNP<-c()
for(j in 1:27)for(i in 3:(nrow(FST)-2)){
if(FST[i,1]==j)(winfiveFST[i]=sum(FST[(i-2):(i+2),5])/5)
if(FST[i,1]==j)(winfiveTheta[i]=sum(FST[(i-2):(i+2),6])/5)
if(FST[i,1]==j)(POS1[i]<-FST[(i-2),3])
if(FST[i,1]==j)(POS2[i]<-FST[(i+2),3])
if(FST[i,1]==j)(POS3[i]<-FST[(i),3])
if(FST[i,1]==j)(CHR[i]<-FST[(i-2),1])
if(FST[i,1]==j)(SNP[i]<-as.vector(FST[(i),2]))
}
win5f<-cbind(CHR,SNP,POS3,winfiveFST,winfiveTheta)
winFST<-cbind(CHR,SNP,POS1,POS2,winfiveFST,winfiveTheta)
win5F<-win5f[complete.cases(win5f),]
colnames(win5F)<-c("CHR","SNP","POS","win5Fst","win5Theta")
write.table(win5F,"win5Fst", sep = "\t", quote = FALSE, row.names = FALSE,col.names =
TRUE)

library(qqman)
win5F<-read.table(file="win5Fst",header=TRUE)
```

```
manhattan(win5F,chr="CHR",bp="POS",p="win5Fst",snp="SNP",col=c("red","blue","green","black"),genomewideline=quantile(as.numeric(win5F[,4]),0.99),logp=FALSE,xlab="Chromosome",ylab="win5Fst")

savePlot(file="manhattanFst", type="pdf")


######################XP-EHH##################

library(rehh)

system("plink --file BIB.QC --remove BI.QC.fam --recode vcf --sheep --out AB")

system("plink --file BIB.QC --remove B.QC.fam --recode vcf --sheep --out IB")

hh.IRI.chr<-data2haplohh(hap_file="AB.vcf",min_maf=0.01,allele_coding="01",polarize_vcf=FALSE,chr.name=1)

hh.ROO.chr<-data2haplohh(hap_file="IB.vcf",min_maf=0.01,allele_coding="01",polarize_vcf=FALSE,chr.name=1)

scan.pop1.chr<-scan_hh(hh.IRI.chr,limhaplo=5,limehh=0.05,limehhs=0.05,threads=1,discard_integration_at_border=FALSE,phased=TRUE,polarized=TRUE)

scan.pop2.chr<-scan_hh(hh.ROO.chr,limhaplo=5,limehh=0.05,limehhs=0.05,threads=1,discard_integration_at_border=FALSE,phased=TRUE,polarized=TRUE)

xp_ehh.chr1<-ies2xpehh(scan.pop1.chr,scan.pop2.chr,popname1=NA,popname2=NA,standardize=TRUE,p.adjust.method="none")


hh.IRI.chr<-data2haplohh(hap_file="AB.vcf",min_maf=0.01,allele_coding="01",polarize_vcf=FALSE,chr.name=2)

hh.ROO.chr<-data2haplohh(hap_file="IB.vcf",min_maf=0.01,allele_coding="01",polarize_vcf=FALSE,chr.name=2)
```

```
scan.pop1.chr<-
scan_hh(hh.IRI.chr,limhaplo=5,limehh=0.05,limehhs=0.05,threads=1,discard_integration_at_border=FALSE,phased=TRUE,polarized=TRUE)

scan.pop2.chr<-
scan_hh(hh.ROO.chr,limhaplo=5,limehh=0.05,limehhs=0.05,threads=1,discard_integration_at_border=FALSE,phased=TRUE,polarized=TRUE)

xp_ehh.chr2<-
ies2xpehh(scan.pop1.chr,scan.pop2.chr,popname1=NA,popname2=NA,standardize=TRUE,p.adjust.method="none")


hh.IRI.chr<-
data2haplohh(hap_file="AB.vcf",min_maf=0.01,allele_coding="01",polarize_vcf=FALSE,chr.name=3)

hh.ROO.chr<-
data2haplohh(hap_file="IB.vcf",min_maf=0.01,allele_coding="01",polarize_vcf=FALSE,chr.name=3)

scan.pop1.chr<-
scan_hh(hh.IRI.chr,limhaplo=5,limehh=0.05,limehhs=0.05,threads=1,discard_integration_at_border=FALSE,phased=TRUE,polarized=TRUE)

scan.pop2.chr<-
scan_hh(hh.ROO.chr,limhaplo=5,limehh=0.05,limehhs=0.05,threads=1,discard_integration_at_border=FALSE,phased=TRUE,polarized=TRUE)

xp_ehh.chr3<-
ies2xpehh(scan.pop1.chr,scan.pop2.chr,popname1=NA,popname2=NA,standardize=TRUE,p.adjust.method="none")


hh.IRI.chr<-
data2haplohh(hap_file="AB.vcf",min_maf=0.01,allele_coding="01",polarize_vcf=FALSE,chr.name=4)

hh.ROO.chr<-
data2haplohh(hap_file="IB.vcf",min_maf=0.01,allele_coding="01",polarize_vcf=FALSE,chr.name=4)
```

```
scan.pop1.chr<-
scan_hh(hh.IRI.chr,limhaplo=5,limehh=0.05,limehhs=0.05,threads=1,discard_integration_at_bor
der=FALSE,phased=TRUE,polarized=TRUE)

scan.pop2.chr<-
scan_hh(hh.ROO.chr,limhaplo=5,limehh=0.05,limehhs=0.05,threads=1,discard_integration_at_b
order=FALSE,phased=TRUE,polarized=TRUE)

xp_ehh.chr4<-
ies2xpehh(scan.pop1.chr,scan.pop2.chr,popname1=NA,popname2=NA,standardize=TRUE,p.adj
ust.method="none")


hh.IRI.chr<-
data2haplohh(hap_file="AB.vcf",min_maf=0.01,allele_coding="01",polarize_vcf=FALSE,chr.na
me=5)

hh.ROO.chr<-
data2haplohh(hap_file="IB.vcf",min_maf=0.01,allele_coding="01",polarize_vcf=FALSE,chr.na
me=5)

scan.pop1.chr<-
scan_hh(hh.IRI.chr,limhaplo=5,limehh=0.05,limehhs=0.05,threads=1,discard_integration_at_bor
der=FALSE,phased=TRUE,polarized=TRUE)

scan.pop2.chr<-
scan_hh(hh.ROO.chr,limhaplo=5,limehh=0.05,limehhs=0.05,threads=1,discard_integration_at_b
order=FALSE,phased=TRUE,polarized=TRUE)

xp_ehh.chr5<-
ies2xpehh(scan.pop1.chr,scan.pop2.chr,popname1=NA,popname2=NA,standardize=TRUE,p.adj
ust.method="none")


hh.IRI.chr<-
data2haplohh(hap_file="AB.vcf",min_maf=0.01,allele_coding="01",polarize_vcf=FALSE,chr.na
me=6)

hh.ROO.chr<-
data2haplohh(hap_file="IB.vcf",min_maf=0.01,allele_coding="01",polarize_vcf=FALSE,chr.na
me=6)
```

```
scan.pop1.chr<-
scan_hh(hh.IRI.chr,limhaplo=5,limehh=0.05,limehhs=0.05,threads=1,discard_integration_at_border=FALSE,phased=TRUE,polarized=TRUE)

scan.pop2.chr<-
scan_hh(hh.ROO.chr,limhaplo=5,limehh=0.05,limehhs=0.05,threads=1,discard_integration_at_border=FALSE,phased=TRUE,polarized=TRUE)

xp_ehh.chr6<-
ies2xpehh(scan.pop1.chr,scan.pop2.chr,popname1=NA,popname2=NA,standardize=TRUE,p.adjust.method="none")


hh.IRI.chr<-
data2haplohh(hap_file="AB.vcf",min_maf=0.01,allele_coding="01",polarize_vcf=FALSE,chr.name=7)

hh.ROO.chr<-
data2haplohh(hap_file="IB.vcf",min_maf=0.01,allele_coding="01",polarize_vcf=FALSE,chr.name=7)

scan.pop1.chr<-
scan_hh(hh.IRI.chr,limhaplo=5,limehh=0.05,limehhs=0.05,threads=1,discard_integration_at_border=FALSE,phased=TRUE,polarized=TRUE)

scan.pop2.chr<-
scan_hh(hh.ROO.chr,limhaplo=5,limehh=0.05,limehhs=0.05,threads=1,discard_integration_at_border=FALSE,phased=TRUE,polarized=TRUE)

xp_ehh.chr7<-
ies2xpehh(scan.pop1.chr,scan.pop2.chr,popname1=NA,popname2=NA,standardize=TRUE,p.adjust.method="none")


hh.IRI.chr<-
data2haplohh(hap_file="AB.vcf",min_maf=0.01,allele_coding="01",polarize_vcf=FALSE,chr.name=8)

hh.ROO.chr<-
data2haplohh(hap_file="IB.vcf",min_maf=0.01,allele_coding="01",polarize_vcf=FALSE,chr.name=8)
```

```
scan.pop1.chr<-
scan_hh(hh.IRI.chr,limhaplo=5,limehh=0.05,limehhs=0.05,threads=1,discard_integration_at_bor
der=FALSE,phased=TRUE,polarized=TRUE)

scan.pop2.chr<-
scan_hh(hh.ROO.chr,limhaplo=5,limehh=0.05,limehhs=0.05,threads=1,discard_integration_at_b
order=FALSE,phased=TRUE,polarized=TRUE)

xp_ehh.chr8<-
ies2xpehh(scan.pop1.chr,scan.pop2.chr,popname1=NA,popname2=NA,standardize=TRUE,p.adj
ust.method="none")


hh.IRI.chr<-
data2haplohh(hap_file="AB.vcf",min_maf=0.01,allele_coding="01",polarize_vcf=FALSE,chr.na
me=9)

hh.ROO.chr<-
data2haplohh(hap_file="IB.vcf",min_maf=0.01,allele_coding="01",polarize_vcf=FALSE,chr.na
me=9)

scan.pop1.chr<-
scan_hh(hh.IRI.chr,limhaplo=5,limehh=0.05,limehhs=0.05,threads=1,discard_integration_at_bor
der=FALSE,phased=TRUE,polarized=TRUE)

scan.pop2.chr<-
scan_hh(hh.ROO.chr,limhaplo=5,limehh=0.05,limehhs=0.05,threads=1,discard_integration_at_b
order=FALSE,phased=TRUE,polarized=TRUE)

xp_ehh.chr9<-
ies2xpehh(scan.pop1.chr,scan.pop2.chr,popname1=NA,popname2=NA,standardize=TRUE,p.adj
ust.method="none")


hh.IRI.chr<-
data2haplohh(hap_file="AB.vcf",min_maf=0.01,allele_coding="01",polarize_vcf=FALSE,chr.na
me=10)

hh.ROO.chr<-
data2haplohh(hap_file="IB.vcf",min_maf=0.01,allele_coding="01",polarize_vcf=FALSE,chr.na
me=10)
```

scan.pop1.chr<-
scan_hh(hh.IRI.chr,limhaplo=5,limehh=0.05,limehhs=0.05,threads=1,discard_integration_at_bor
der=FALSE,phased=TRUE,polarized=TRUE)

scan.pop2.chr<-
scan_hh(hh.ROO.chr,limhaplo=5,limehh=0.05,limehhs=0.05,threads=1,discard_integration_at_b
order=FALSE,phased=TRUE,polarized=TRUE)

xp_ehh.chr10<-
ies2xpehh(scan.pop1.chr,scan.pop2.chr,popname1=NA,popname2=NA,standardize=TRUE,p.adj
ust.method="none")

hh.IRI.chr<-
data2haplohh(hap_file="AB.vcf",min_maf=0.01,allele_coding="01",polarize_vcf=FALSE,chr.na
me=11)

hh.ROO.chr<-
data2haplohh(hap_file="IB.vcf",min_maf=0.01,allele_coding="01",polarize_vcf=FALSE,chr.na
me=11)

scan.pop1.chr<-
scan_hh(hh.IRI.chr,limhaplo=5,limehh=0.05,limehhs=0.05,threads=1,discard_integration_at_bor
der=FALSE,phased=TRUE,polarized=TRUE)

scan.pop2.chr<-
scan_hh(hh.ROO.chr,limhaplo=5,limehh=0.05,limehhs=0.05,threads=1,discard_integration_at_b
order=FALSE,phased=TRUE,polarized=TRUE)

xp_ehh.chr11<-
ies2xpehh(scan.pop1.chr,scan.pop2.chr,popname1=NA,popname2=NA,standardize=TRUE,p.adj
ust.method="none")

hh.IRI.chr<-
data2haplohh(hap_file="AB.vcf",min_maf=0.01,allele_coding="01",polarize_vcf=FALSE,chr.na
me=12)

hh.ROO.chr<-
data2haplohh(hap_file="IB.vcf",min_maf=0.01,allele_coding="01",polarize_vcf=FALSE,chr.na
me=12)

```
scan.pop1.chr<-
scan_hh(hh.IRI.chr,limhaplo=5,limehh=0.05,limehhs=0.05,threads=1,discard_integration_at_bor
der=FALSE,phased=TRUE,polarized=TRUE)

scan.pop2.chr<-
scan_hh(hh.ROO.chr,limhaplo=5,limehh=0.05,limehhs=0.05,threads=1,discard_integration_at_b
order=FALSE,phased=TRUE,polarized=TRUE)

xp_ehh.chr12<-
ies2xpehh(scan.pop1.chr,scan.pop2.chr,popname1=NA,popname2=NA,standardize=TRUE,p.adj
ust.method="none")


hh.IRI.chr<-
data2haplohh(hap_file="AB.vcf",min_maf=0.01,allele_coding="01",polarize_vcf=FALSE,chr.na
me=13)

hh.ROO.chr<-
data2haplohh(hap_file="IB.vcf",min_maf=0.01,allele_coding="01",polarize_vcf=FALSE,chr.na
me=13)

scan.pop1.chr<-
scan_hh(hh.IRI.chr,limhaplo=5,limehh=0.05,limehhs=0.05,threads=1,discard_integration_at_bor
der=FALSE,phased=TRUE,polarized=TRUE)

scan.pop2.chr<-
scan_hh(hh.ROO.chr,limhaplo=5,limehh=0.05,limehhs=0.05,threads=1,discard_integration_at_b
order=FALSE,phased=TRUE,polarized=TRUE)

xp_ehh.chr13<-
ies2xpehh(scan.pop1.chr,scan.pop2.chr,popname1=NA,popname2=NA,standardize=TRUE,p.adj
ust.method="none")


hh.IRI.chr<-
data2haplohh(hap_file="AB.vcf",min_maf=0.01,allele_coding="01",polarize_vcf=FALSE,chr.na
me=14)

hh.ROO.chr<-
data2haplohh(hap_file="IB.vcf",min_maf=0.01,allele_coding="01",polarize_vcf=FALSE,chr.na
me=14)
```

scan.pop1.chr<-
scan_hh(hh.IRI.chr,limhaplo=5,limehh=0.05,limehhs=0.05,threads=1,discard_integration_at_bor
der=FALSE,phased=TRUE,polarized=TRUE)

scan.pop2.chr<-
scan_hh(hh.ROO.chr,limhaplo=5,limehh=0.05,limehhs=0.05,threads=1,discard_integration_at_b
order=FALSE,phased=TRUE,polarized=TRUE)

xp_ehh.chr14<-
ies2xpehh(scan.pop1.chr,scan.pop2.chr,popname1=NA,popname2=NA,standardize=TRUE,p.adj
ust.method="none")

hh.IRI.chr<-
data2haplohh(hap_file="AB.vcf",min_maf=0.01,allele_coding="01",polarize_vcf=FALSE,chr.na
me=15)

hh.ROO.chr<-
data2haplohh(hap_file="IB.vcf",min_maf=0.01,allele_coding="01",polarize_vcf=FALSE,chr.na
me=15)

scan.pop1.chr<-
scan_hh(hh.IRI.chr,limhaplo=5,limehh=0.05,limehhs=0.05,threads=1,discard_integration_at_bor
der=FALSE,phased=TRUE,polarized=TRUE)

scan.pop2.chr<-
scan_hh(hh.ROO.chr,limhaplo=5,limehh=0.05,limehhs=0.05,threads=1,discard_integration_at_b
order=FALSE,phased=TRUE,polarized=TRUE)

xp_ehh.chr15<-
ies2xpehh(scan.pop1.chr,scan.pop2.chr,popname1=NA,popname2=NA,standardize=TRUE,p.adj
ust.method="none")

hh.IRI.chr<-
data2haplohh(hap_file="AB.vcf",min_maf=0.01,allele_coding="01",polarize_vcf=FALSE,chr.na
me=16)

hh.ROO.chr<-
data2haplohh(hap_file="IB.vcf",min_maf=0.01,allele_coding="01",polarize_vcf=FALSE,chr.na
me=16)

```
scan.pop1.chr<-
scan_hh(hh.IRI.chr,limhaplo=5,limehh=0.05,limehhs=0.05,threads=1,discard_integration_at_bor
der=FALSE,phased=TRUE,polarized=TRUE)

scan.pop2.chr<-
scan_hh(hh.ROO.chr,limhaplo=5,limehh=0.05,limehhs=0.05,threads=1,discard_integration_at_b
order=FALSE,phased=TRUE,polarized=TRUE)

xp_ehh.chr16<-
ies2xpehh(scan.pop1.chr,scan.pop2.chr,popname1=NA,popname2=NA,standardize=TRUE,p.adj
ust.method="none")


hh.IRI.chr<-
data2haplohh(hap_file="AB.vcf",min_maf=0.01,allele_coding="01",polarize_vcf=FALSE,chr.na
me=17)

hh.ROO.chr<-
data2haplohh(hap_file="IB.vcf",min_maf=0.01,allele_coding="01",polarize_vcf=FALSE,chr.na
me=17)

scan.pop1.chr<-
scan_hh(hh.IRI.chr,limhaplo=5,limehh=0.05,limehhs=0.05,threads=1,discard_integration_at_bor
der=FALSE,phased=TRUE,polarized=TRUE)

scan.pop2.chr<-
scan_hh(hh.ROO.chr,limhaplo=5,limehh=0.05,limehhs=0.05,threads=1,discard_integration_at_b
order=FALSE,phased=TRUE,polarized=TRUE)

xp_ehh.chr17<-
ies2xpehh(scan.pop1.chr,scan.pop2.chr,popname1=NA,popname2=NA,standardize=TRUE,p.adj
ust.method="none")


hh.IRI.chr<-
data2haplohh(hap_file="AB.vcf",min_maf=0.01,allele_coding="01",polarize_vcf=FALSE,chr.na
me=18)

hh.ROO.chr<-
data2haplohh(hap_file="IB.vcf",min_maf=0.01,allele_coding="01",polarize_vcf=FALSE,chr.na
me=18)
```

```
scan.pop1.chr<-
scan_hh(hh.IRI.chr,limhaplo=5,limehh=0.05,limehhs=0.05,threads=1,discard_integration_at_border=FALSE,phased=TRUE,polarized=TRUE)

scan.pop2.chr<-
scan_hh(hh.ROO.chr,limhaplo=5,limehh=0.05,limehhs=0.05,threads=1,discard_integration_at_border=FALSE,phased=TRUE,polarized=TRUE)

xp_ehh.chr18<-
ies2xpehh(scan.pop1.chr,scan.pop2.chr,popname1=NA,popname2=NA,standardize=TRUE,p.adjust.method="none")


hh.IRI.chr<-
data2haplohh(hap_file="AB.vcf",min_maf=0.01,allele_coding="01",polarize_vcf=FALSE,chr.name=19)

hh.ROO.chr<-
data2haplohh(hap_file="IB.vcf",min_maf=0.01,allele_coding="01",polarize_vcf=FALSE,chr.name=19)

scan.pop1.chr<-
scan_hh(hh.IRI.chr,limhaplo=5,limehh=0.05,limehhs=0.05,threads=1,discard_integration_at_border=FALSE,phased=TRUE,polarized=TRUE)

scan.pop2.chr<-
scan_hh(hh.ROO.chr,limhaplo=5,limehh=0.05,limehhs=0.05,threads=1,discard_integration_at_border=FALSE,phased=TRUE,polarized=TRUE)

xp_ehh.chr19<-
ies2xpehh(scan.pop1.chr,scan.pop2.chr,popname1=NA,popname2=NA,standardize=TRUE,p.adjust.method="none")


hh.IRI.chr<-
data2haplohh(hap_file="AB.vcf",min_maf=0.01,allele_coding="01",polarize_vcf=FALSE,chr.name=20)

hh.ROO.chr<-
data2haplohh(hap_file="IB.vcf",min_maf=0.01,allele_coding="01",polarize_vcf=FALSE,chr.name=20)
```

```
scan.pop1.chr<-
scan_hh(hh.IRI.chr,limhaplo=5,limehh=0.05,limehhs=0.05,threads=1,discard_integration_at_bor
der=FALSE,phased=TRUE,polarized=TRUE)

scan.pop2.chr<-
scan_hh(hh.ROO.chr,limhaplo=5,limehh=0.05,limehhs=0.05,threads=1,discard_integration_at_b
order=FALSE,phased=TRUE,polarized=TRUE)

xp_ehh.chr20<-
ies2xpehh(scan.pop1.chr,scan.pop2.chr,popname1=NA,popname2=NA,standardize=TRUE,p.adj
ust.method="none")


hh.IRI.chr<-
data2haplohh(hap_file="AB.vcf",min_maf=0.01,allele_coding="01",polarize_vcf=FALSE,chr.na
me=21)

hh.ROO.chr<-
data2haplohh(hap_file="IB.vcf",min_maf=0.01,allele_coding="01",polarize_vcf=FALSE,chr.na
me=21)

scan.pop1.chr<-
scan_hh(hh.IRI.chr,limhaplo=5,limehh=0.05,limehhs=0.05,threads=1,discard_integration_at_bor
der=FALSE,phased=TRUE,polarized=TRUE)

scan.pop2.chr<-
scan_hh(hh.ROO.chr,limhaplo=5,limehh=0.05,limehhs=0.05,threads=1,discard_integration_at_b
order=FALSE,phased=TRUE,polarized=TRUE)

xp_ehh.chr21<-
ies2xpehh(scan.pop1.chr,scan.pop2.chr,popname1=NA,popname2=NA,standardize=TRUE,p.adj
ust.method="none")


hh.IRI.chr<-
data2haplohh(hap_file="AB.vcf",min_maf=0.01,allele_coding="01",polarize_vcf=FALSE,chr.na
me=22)

hh.ROO.chr<-
data2haplohh(hap_file="IB.vcf",min_maf=0.01,allele_coding="01",polarize_vcf=FALSE,chr.na
me=22)
```

```
scan.pop1.chr<-
scan_hh(hh.IRI.chr,limhaplo=5,limehh=0.05,limehhs=0.05,threads=1,discard_integration_at_bor
der=FALSE,phased=TRUE,polarized=TRUE)

scan.pop2.chr<-
scan_hh(hh.ROO.chr,limhaplo=5,limehh=0.05,limehhs=0.05,threads=1,discard_integration_at_b
order=FALSE,phased=TRUE,polarized=TRUE)

xp_ehh.chr22<-
ies2xpehh(scan.pop1.chr,scan.pop2.chr,popname1=NA,popname2=NA,standardize=TRUE,p.adj
ust.method="none")


hh.IRI.chr<-
data2haplohh(hap_file="AB.vcf",min_maf=0.01,allele_coding="01",polarize_vcf=FALSE,chr.na
me=23)

hh.ROO.chr<-
data2haplohh(hap_file="IB.vcf",min_maf=0.01,allele_coding="01",polarize_vcf=FALSE,chr.na
me=23)

scan.pop1.chr<-
scan_hh(hh.IRI.chr,limhaplo=5,limehh=0.05,limehhs=0.05,threads=1,discard_integration_at_bor
der=FALSE,phased=TRUE,polarized=TRUE)

scan.pop2.chr<-
scan_hh(hh.ROO.chr,limhaplo=5,limehh=0.05,limehhs=0.05,threads=1,discard_integration_at_b
order=FALSE,phased=TRUE,polarized=TRUE)

xp_ehh.chr23<-
ies2xpehh(scan.pop1.chr,scan.pop2.chr,popname1=NA,popname2=NA,standardize=TRUE,p.adj
ust.method="none")


hh.IRI.chr<-
data2haplohh(hap_file="AB.vcf",min_maf=0.01,allele_coding="01",polarize_vcf=FALSE,chr.na
me=24)

hh.ROO.chr<-
data2haplohh(hap_file="IB.vcf",min_maf=0.01,allele_coding="01",polarize_vcf=FALSE,chr.na
me=24)
```

```
scan.pop1.chr<-
scan_hh(hh.IRI.chr,limhaplo=5,limehh=0.05,limehhs=0.05,threads=1,discard_integration_at_bor
der=FALSE,phased=TRUE,polarized=TRUE)

scan.pop2.chr<-
scan_hh(hh.ROO.chr,limhaplo=5,limehh=0.05,limehhs=0.05,threads=1,discard_integration_at_b
order=FALSE,phased=TRUE,polarized=TRUE)

xp_ehh.chr24<-
ies2xpehh(scan.pop1.chr,scan.pop2.chr,popname1=NA,popname2=NA,standardize=TRUE,p.adj
ust.method="none")


hh.IRI.chr<-
data2haplohh(hap_file="AB.vcf",min_maf=0.01,allele_coding="01",polarize_vcf=FALSE,chr.na
me=25)

hh.ROO.chr<-
data2haplohh(hap_file="IB.vcf",min_maf=0.01,allele_coding="01",polarize_vcf=FALSE,chr.na
me=25)

scan.pop1.chr<-
scan_hh(hh.IRI.chr,limhaplo=5,limehh=0.05,limehhs=0.05,threads=1,discard_integration_at_bor
der=FALSE,phased=TRUE,polarized=TRUE)

scan.pop2.chr<-
scan_hh(hh.ROO.chr,limhaplo=5,limehh=0.05,limehhs=0.05,threads=1,discard_integration_at_b
order=FALSE,phased=TRUE,polarized=TRUE)

xp_ehh.chr25<-
ies2xpehh(scan.pop1.chr,scan.pop2.chr,popname1=NA,popname2=NA,standardize=TRUE,p.adj
ust.method="none")


hh.IRI.chr<-
data2haplohh(hap_file="AB.vcf",min_maf=0.01,allele_coding="01",polarize_vcf=FALSE,chr.na
me=26)

hh.ROO.chr<-
data2haplohh(hap_file="IB.vcf",min_maf=0.01,allele_coding="01",polarize_vcf=FALSE,chr.na
me=26)
```

```
scan.pop1.chr<-
scan_hh(hh.IRI.chr,limhaplo=5,limehh=0.05,limehhs=0.05,threads=1,discard_integration_at_bor
der=FALSE,phased=TRUE,polarized=TRUE)

scan.pop2.chr<-
scan_hh(hh.ROO.chr,limhaplo=5,limehh=0.05,limehhs=0.05,threads=1,discard_integration_at_b
order=FALSE,phased=TRUE,polarized=TRUE)

xp_ehh.chr26<-
ies2xpehh(scan.pop1.chr,scan.pop2.chr,popname1=NA,popname2=NA,standardize=TRUE,p.adj
ust.method="none")


hh.IRI.chr<-
data2haplohh(hap_file="AB.vcf",min_maf=0.01,allele_coding="01",polarize_vcf=FALSE,chr.na
me=27)

hh.ROO.chr<-
data2haplohh(hap_file="IB.vcf",min_maf=0.01,allele_coding="01",polarize_vcf=FALSE,chr.na
me=27)

scan.pop1.chr<-
scan_hh(hh.IRI.chr,limhaplo=5,limehh=0.05,limehhs=0.05,threads=1,discard_integration_at_bor
der=FALSE,phased=TRUE,polarized=TRUE)

scan.pop2.chr<-
scan_hh(hh.ROO.chr,limhaplo=5,limehh=0.05,limehhs=0.05,threads=1,discard_integration_at_b
order=FALSE,phased=TRUE,polarized=TRUE)

xp_ehh.chr27<-
ies2xpehh(scan.pop1.chr,scan.pop2.chr,popname1=NA,popname2=NA,standardize=TRUE,p.adj
ust.method="none")


xp_ehh<-
as.matrix(rbind(xp_ehh.chr1,xp_ehh.chr2,xp_ehh.chr3,xp_ehh.chr4,xp_ehh.chr5,xp_ehh.chr6,xp
_ehh.chr7,xp_ehh.chr8,xp_ehh.chr9,xp_ehh.chr10,xp_ehh.chr11,xp_ehh.chr12,xp_ehh.chr13,xp
_ehh.chr14,xp_ehh.chr15,xp_ehh.chr16,xp_ehh.chr17,xp_ehh.chr18,xp_ehh.chr19,xp_ehh.chr20
,xp_ehh.chr21,xp_ehh.chr22,xp_ehh.chr23,xp_ehh.chr24,xp_ehh.chr25,xp_ehh.chr26,xp_ehh.ch
r27))

x<-row.names(xp_ehh)

xp_ehh<-cbind(x,xp_ehh)
```

```
xp<-cbind(xp_ehh[,2],xp_ehh[,-c(2,4)],1,1,1,1,1,xp_ehh[,4])

colnames(xp)<-c("CHR","SNP","BP","A1","TEST","NMISS","BETA","STAT","xp_ehh")

write.table(xp,"xp_ehh", sep = "\t", quote = FALSE, row.names = FALSE,col.names = TRUE)



library(rehh)

xp<-read.table(file="xp_ehh",header=TRUE)

manhattanplot(xp,threshold=c(quantile(as.numeric(xp_ehh[,9]),0.99),quantile(as.numeric(xp_eh
h[,9]),0.01)),cex=0.8,chr.name=c(1:27))

savePlot(file="manhattan-xpehhtf", type="pdf")


######## gene and QTL report ####################


Fst<-read.table(file="win5Fst",skip=1)

Fst[,5]<-abs(Fst[,5])

Fst.adj<-Fst[as.numeric(Fst[,5])>quantile(as.numeric(Fst[,5]),0.99),]

write.table(Fst.adj,"win5Fst.adj", sep = "\t", quote = FALSE, row.names = FALSE,col.names =
TRUE)

rm(list=ls())



f<-as.matrix(read.table(file="win5Fst.adj",skip=1))

f<-cbind(f[,1:3],1,1,1,1,1,f[,5])

colnames(f)<-c("CHR","SNP","BP","A1","TEST","NMISS","BETA","STAT","win5Fst")

write.table(f,"win.assoc", sep = "\t", quote = FALSE, row.names = FALSE,col.names = TRUE)

rm(list=ls())


shell("plink --gene-report win.assoc --gene-list gene.list --sheep --out geneFST")
```

```
shell("plink --gene-report win.assoc --gene-list QTL.list --sheep --out QTLFST")


xp<-read.table(file="xp_ehh",header=T)

lowxp<-quantile(as.numeric(xp[,9]),0.01)

highxp<-quantile(as.numeric(xp[,9]),0.99)

win5adjxp<-xp[as.numeric(xp[,9])>highxp | as.numeric(xp[,9])<lowxp,]

write.table(win5adjxp,"xpehh.adj", sep = "\t", quote = FALSE, row.names = FALSE,col.names = TRUE)


shell("plink --gene-report xpehh.adj --gene-list gene.list --sheep --out geneehh")

shell("plink --gene-report xpehh.adj --gene-list QTL.list --sheep --out QTLehh")


##############################
```
