# Supplementary material for: Genetic diversity, population structure, and combined detection of selection signatures in Iranian versus Afghan Baluchi sheep
Source: PLoS One. 2026 Jun 17;21(6):e0350262. doi: 10.1371/journal.pone.0350262 (PMC13274857; doi:10.1371/journal.pone.0350262)
Supplement: S2 Fig — (PDF) [file pone.0350262.s004.pdf]

S1 Fig. ADMIXTURE analysis results for K = 3 and K = 4, for individuals in the AB (1-15 Individual) and IB (16-101 Individual) populations

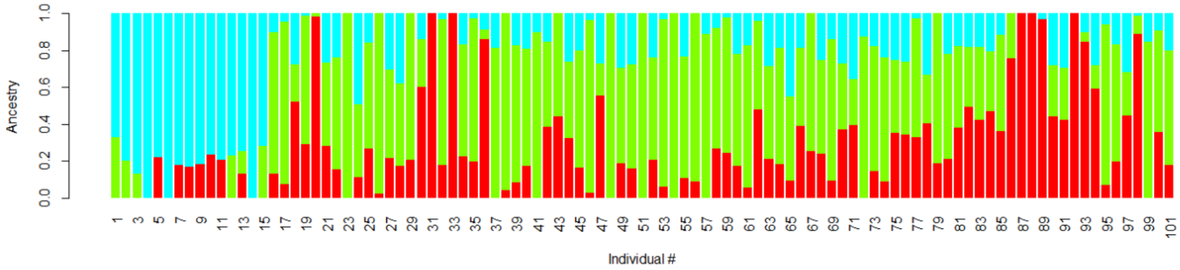

K=3

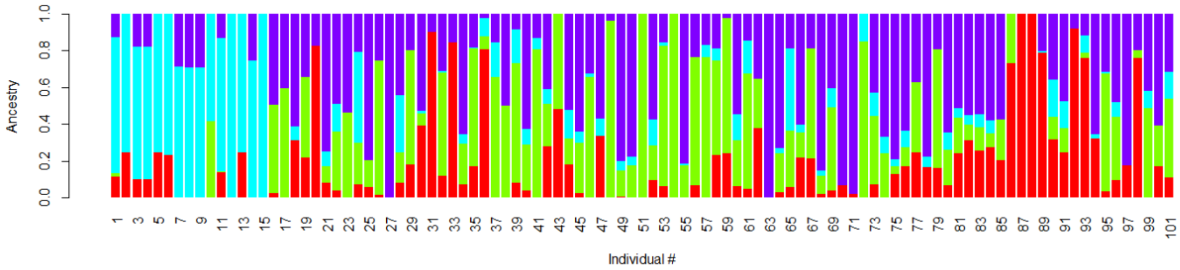

K=4
