# Supplementary material for: Genetic diversity, population structure, and combined detection of selection signatures in Iranian versus Afghan Baluchi sheep
Source: PLoS One. 2026 Jun 17;21(6):e0350262. doi: 10.1371/journal.pone.0350262 (PMC13274857; doi:10.1371/journal.pone.0350262)
Supplement: S2 Table — (PDF) [file pone.0350262.s005.pdf]

S2 Table. Number of pairwise SNP comparisons contributing to each distance bin

bin	mid_kb	mean_r2	sd_r2	n_pairs	se_r2	ci_low	ci_high	population
[0,10)	7.63612508	0.27042974	0.2980932	1615	0.00741764	0.25589116	0.28496831	AB
[10,20)	15.204405	0.21513992	0.24969646	3802	0.00404955	0.20720281	0.22307703	AB
[20,30)	25.3450231	0.18666263	0.22863082	5360	0.00312286	0.18054183	0.19278343	AB
[30,40)	35.0955286	0.17086032	0.21805576	7718	0.00248208	0.16599545	0.17572519	AB
[40,50)	45.0620126	0.1642435	0.20833918	7645	0.00238277	0.15957327	0.16891373	AB
[50,60)	55.0613636	0.14918928	0.19354585	8196	0.00213788	0.14499904	0.15337952	AB
[60,70)	64.7889419	0.13347642	0.17777546	8089	0.00197663	0.12960223	0.1373506	AB
[70,80)	75.0020405	0.11971184	0.1606972	7414	0.0018663	0.11605389	0.12336979	AB
[80,90)	84.9455739	0.11637831	0.15879289	7726	0.00180657	0.11283744	0.11991917	AB
[90,100)	95.0255651	0.11412297	0.15289805	7719	0.00174029	0.110712	0.11753394	AB
[100,110)	104.993792	0.10850701	0.14604089	7746	0.00165934	0.1052547	0.11175931	AB
[110,120)	114.959479	0.10325344	0.13797059	7925	0.00154984	0.10021575	0.10629113	AB
[120,130)	124.991115	0.09943996	0.13547028	7507	0.00156355	0.09637541	0.10250451	AB
[130,140)	135.01591	0.09737573	0.13067003	7663	0.00149271	0.09445001	0.10030145	AB
[140,150)	145.014334	0.09617763	0.12744189	7610	0.0014609	0.09331427	0.09904099	AB
[150,160)	154.994245	0.09558951	0.12785396	7690	0.00145798	0.09273187	0.09844715	AB
[160,170)	164.969697	0.09096123	0.12180007	7646	0.00139293	0.08823108	0.09369138	AB
[170,180)	174.984247	0.08958195	0.11754099	7575	0.00135051	0.08693495	0.09222895	AB
[180,190)	185.049756	0.09308623	0.12219751	7631	0.00139885	0.09034448	0.09582798	AB
[190,200)	195.003807	0.08927255	0.11983196	7522	0.00138168	0.08656447	0.09198064	AB
[200,210)	204.983477	0.08714427	0.11243518	7636	0.00128668	0.08462239	0.08966616	AB
[210,220)	214.988971	0.08794939	0.11411172	7456	0.00132153	0.08535919	0.09053959	AB
[220,230)	225.02709	0.08645217	0.11295339	7697	0.00128747	0.08392872	0.08897562	AB
[230,240)	234.966019	0.08598326	0.11248859	7590	0.00129118	0.08345254	0.08851398	AB
[240,250)	245.039304	0.08246565	0.10880401	7653	0.00124374	0.08002792	0.08490338	AB
[250,260)	255.037224	0.08612366	0.11028843	7455	0.00127734	0.08362007	0.08862724	AB
[260,270)	264.972692	0.08497735	0.11005935	7643	0.00125891	0.08250988	0.08744481	AB
[270,280)	274.993292	0.08312286	0.10740329	7530	0.00123771	0.08069694	0.08554877	AB
[280,290)	285.019675	0.08369652	0.10873733	7484	0.00125693	0.08123293	0.08616011	AB
[290,300)	295.028778	0.08222489	0.10894808	7646	0.00124596	0.07978281	0.08466696	AB
[300,310)	304.984827	0.08161487	0.10527964	7427	0.00122162	0.07922049	0.08400926	AB
[310,320)	314.930233	0.08286032	0.10614708	7535	0.00122283	0.08046357	0.08525707	AB
[320,330)	324.934677	0.08154861	0.10492833	7546	0.00120791	0.07918111	0.08391611	AB
[330,340)	335.026237	0.08021838	0.10445651	7535	0.00120336	0.0778598	0.08257696	AB
[340,350)	345.038994	0.08292705	0.10695019	7491	0.0012357	0.08050509	0.08534902	AB
[350,360)	355.005811	0.08256947	0.10947407	7561	0.00125899	0.08010186	0.08503709	AB
[360,370)	364.987269	0.08067653	0.1046846	7454	0.00121252	0.0783	0.08305306	AB
[370,380)	374.983057	0.08113614	0.10714466	7359	0.001249	0.07868811	0.08358418	AB

[380,390)	385.010683	0.07966589	0.10271192	7547	0.00118232	0.07734855	0.08198323	AB
[390,400)	394.98156	0.08067201	0.10461654	7476	0.00120995	0.07830052	0.0830435	AB
[400,410)	405.029947	0.07988081	0.10278787	7520	0.00118531	0.0775576	0.08220402	AB
[410,420)	414.993933	0.08064401	0.10429683	7584	0.00119763	0.07829666	0.08299137	AB
[420,430)	425.040274	0.07975273	0.10271873	7516	0.00118483	0.07743046	0.082075	AB
[430,440)	434.994077	0.07995064	0.10207573	7363	0.00118958	0.07761905	0.08228222	AB
[440,450)	444.980017	0.07992504	0.10233057	7448	0.00118573	0.07760101	0.08224907	AB
[450,460)	454.987215	0.07674778	0.09962944	7395	0.00115856	0.07447701	0.07901856	AB
[460,470)	464.98751	0.07922038	0.10128867	7460	0.00117271	0.07692186	0.0815189	AB
[470,480)	475.049745	0.07812769	0.10031684	7579	0.00115231	0.07586917	0.08038621	AB
[480,490)	484.997159	0.07997553	0.10295884	7596	0.00118133	0.07766012	0.08229094	AB
[490,500)	494.983315	0.07894417	0.09938425	7379	0.00115696	0.07667653	0.08121182	AB
[500,510)	504.966201	0.07933348	0.10196994	7310	0.00119265	0.07699588	0.08167108	AB
[510,520)	515.017347	0.07931571	0.10133746	7464	0.00117296	0.07701671	0.08161472	AB
[520,530)	525.060542	0.07908472	0.10248686	7458	0.00118674	0.0767587	0.08141074	AB
[530,540)	534.970757	0.07845169	0.09951108	7578	0.00114313	0.07621116	0.08069222	AB
[540,550)	545.036187	0.07994387	0.10181942	7324	0.00118975	0.07761196	0.08227578	AB
[550,560)	555.053638	0.07901646	0.10051252	7403	0.0011682	0.07672679	0.08130612	AB
[560,570)	565.015585	0.07883134	0.10092818	7612	0.00115681	0.07656399	0.08109869	AB
[570,580)	575.032892	0.07940286	0.1022073	7410	0.00118733	0.07707569	0.08173003	AB
[580,590)	585.019007	0.07899279	0.10102188	7382	0.00117579	0.07668825	0.08129734	AB
[590,600)	594.964448	0.07732526	0.09851131	7262	0.001156	0.0750595	0.07959102	AB
[600,610)	605.011823	0.07821299	0.09968937	7457	0.00115443	0.07595031	0.08047566	AB
[610,620)	615.009287	0.0812768	0.10364978	7493	0.0011974	0.07892989	0.08362371	AB
[620,630)	625.031942	0.0784208	0.10042968	7408	0.00116684	0.07613379	0.08070781	AB
[630,640)	635.014425	0.0770449	0.09792496	7553	0.00112677	0.07483644	0.07925336	AB
[640,650)	645.018358	0.07985911	0.10111197	7366	0.00117811	0.07755001	0.08216821	AB
[650,660)	654.975203	0.0797458	0.10029459	7306	0.00117338	0.07744598	0.08204562	AB
[660,670)	665.074616	0.07825286	0.09997837	7377	0.00116404	0.07597135	0.08053437	AB
[670,680)	675.029073	0.07779197	0.10123153	7455	0.00117244	0.07549398	0.08008996	AB
[680,690)	684.985236	0.07734797	0.09909874	7470	0.00114659	0.07510066	0.07959529	AB
[690,700)	695.041387	0.08034754	0.10326443	7416	0.00119913	0.07799724	0.08269783	AB
[700,710)	704.956879	0.07945862	0.10095884	7335	0.00117881	0.07714815	0.08176909	AB
[710,720)	715.017082	0.07739103	0.09863182	7569	0.0011337	0.07516898	0.07961308	AB
[720,730)	725.029641	0.07768247	0.09806	7303	0.00114747	0.07543343	0.07993151	AB
[730,740)	734.96896	0.07879526	0.10204447	7473	0.00118043	0.07648161	0.08110891	AB
[740,750)	744.953105	0.08034196	0.10262175	7405	0.00119255	0.07800456	0.08267936	AB
[750,760)	755.066426	0.07809757	0.10001126	7372	0.00116481	0.07581453	0.0803806	AB
[760,770)	764.983582	0.07813158	0.10097305	7342	0.00117842	0.07582189	0.08044127	AB
[770,780)	774.987033	0.07731885	0.09968621	7466	0.0011537	0.0750576	0.07958009	AB
[780,790)	785.047223	0.07793348	0.09961426	7415	0.00115682	0.07566611	0.08020085	AB
[790,800)	795.014613	0.07597493	0.0970548	7481	0.00112211	0.07377558	0.07817427	AB
[800,810)	805.00639	0.07810068	0.0995674	7402	0.00115729	0.07583239	0.08036897	AB

[810,820)	815.016776	0.07798566	0.09853918	7459	0.00114096	0.07574939	0.08022194	AB
[820,830)	824.974281	0.07842151	0.10216125	7259	0.00119908	0.07607132	0.08077171	AB
[830,840)	834.940376	0.07752219	0.09810224	7394	0.00114088	0.07528607	0.07975831	AB
[840,850)	845.046004	0.07832432	0.09990626	7310	0.00116851	0.07603404	0.08061461	AB
[850,860)	855.01115	0.07766841	0.09831257	7454	0.00113871	0.07543653	0.07990029	AB
[860,870)	864.998379	0.07758504	0.09851737	7399	0.00114532	0.07534021	0.07982986	AB
[870,880)	875.023268	0.07856114	0.10065605	7467	0.00116484	0.07627805	0.08084423	AB
[880,890)	884.967105	0.0788842	0.09993085	7263	0.00117258	0.07658595	0.08118246	AB
[890,900)	895.005059	0.0764007	0.09859718	7413	0.00114516	0.07415618	0.07864523	AB
[900,910)	905.037086	0.07941164	0.10020395	7356	0.00116833	0.07712172	0.08170156	AB
[910,920)	915.019646	0.07759853	0.10016893	7384	0.0011657	0.07531375	0.0798833	AB
[920,930)	925.031056	0.07842867	0.09845276	7417	0.00114318	0.07618804	0.0806693	AB
[930,940)	934.96623	0.0759367	0.09731432	7462	0.00112655	0.07372866	0.07814473	AB
[940,950)	945.06001	0.07840484	0.09841475	7333	0.00114926	0.07615228	0.08065739	AB
[950,960)	955.008632	0.07700323	0.0989452	7377	0.00115201	0.0747453	0.07926116	AB
[960,970)	965.006122	0.0789353	0.10071506	7470	0.00116529	0.07665133	0.08121927	AB
[970,980)	975.022556	0.0780662	0.10004054	7365	0.00116571	0.07578141	0.08035099	AB
[980,990)	984.966625	0.07857777	0.09954239	7263	0.00116802	0.07628845	0.08086708	AB
[990,1e+03]	995.017293	0.07796562	0.09945848	7320	0.00116248	0.07568716	0.08024409	AB
[0,10)	7.61986416	0.33856691	0.33179022	1384	0.00891858	0.3210865	0.35604732	IB
[10,20)	15.2678212	0.27429017	0.29042214	3271	0.00507796	0.26433736	0.28424297	IB
[20,30)	25.33647	0.21306408	0.25343291	4551	0.00375673	0.20570089	0.22042726	IB
[30,40)	35.0868981	0.18255217	0.2283537	6534	0.002825	0.17701517	0.18808917	IB
[40,50)	45.0539181	0.17053179	0.21343551	6382	0.0026717	0.16529525	0.17576833	IB
[50,60)	55.0452885	0.15904763	0.20200442	7034	0.00240857	0.15432683	0.16376843	IB
[60,70)	64.762345	0.1430408	0.18954017	6882	0.00228478	0.13856263	0.14751896	IB
[70,80)	74.9806902	0.13378556	0.17652759	6224	0.00223758	0.12939991	0.13817121	IB
[80,90)	84.9773748	0.12769197	0.1674073	6577	0.00206424	0.12364606	0.13173789	IB
[90,100)	95.009701	0.11835503	0.16134311	6549	0.00199371	0.11444735	0.12226271	IB
[100,110)	104.949072	0.11760427	0.15491644	6449	0.00192908	0.11382327	0.12138528	IB
[110,120)	114.947685	0.11201881	0.15347848	6685	0.00187714	0.10833962	0.11569801	IB
[120,130)	125.005739	0.10994652	0.14544905	6415	0.00181599	0.10638719	0.11350585	IB
[130,140)	135.054084	0.10767358	0.14389151	6452	0.00179138	0.10416248	0.11118469	IB
[140,150)	144.996321	0.10650088	0.14330213	6396	0.00179184	0.10298888	0.11001288	IB
[150,160)	155.019216	0.10321935	0.13744315	6564	0.00169644	0.09989433	0.10654438	IB
[160,170)	164.981273	0.09913466	0.13066801	6504	0.00162024	0.095959	0.10231033	IB
[170,180)	175.004426	0.09453012	0.12644648	6368	0.00158455	0.0914244	0.09763583	IB
[180,190)	185.028662	0.10007664	0.13182828	6463	0.0016398	0.09686262	0.10329065	IB
[190,200)	195.031655	0.09573562	0.12803354	6395	0.00160104	0.09259757	0.09887366	IB
[200,210)	204.974525	0.09287276	0.1239323	6469	0.00154087	0.08985266	0.09589287	IB
[210,220)	215.035051	0.09626581	0.12717449	6264	0.00160685	0.09311639	0.09941523	IB
[220,230)	224.997116	0.09336447	0.12409458	6587	0.00152901	0.09036762	0.09636132	IB
[230,240)	234.942327	0.09237698	0.1228562	6349	0.00154186	0.08935494	0.09539902	IB

[240,250)	244.998829	0.0943935	0.1241817	6461	0.00154493	0.09136545	0.09742156	IB
[250,260)	255.007993	0.09265603	0.12185973	6317	0.00153322	0.08965092	0.09566114	IB
[260,270)	265.013496	0.08990826	0.11642273	6486	0.0014456	0.08707488	0.09274165	IB
[270,280)	274.98722	0.09083605	0.12061987	6316	0.00151774	0.08786127	0.09381082	IB
[280,290)	285.014991	0.0888439	0.1176973	6349	0.00147711	0.08594875	0.09173904	IB
[290,300)	295.021466	0.08842102	0.11270605	6513	0.00139655	0.08568378	0.09115826	IB
[300,310)	304.947296	0.08798263	0.1174113	6237	0.0014867	0.08506871	0.09089656	IB
[310,320)	314.939898	0.08888899	0.11368226	6290	0.0014334	0.08607953	0.09169846	IB
[320,330)	324.93446	0.08939817	0.11643282	6398	0.00145564	0.08654512	0.09225122	IB
[330,340)	335.000132	0.08854379	0.11349296	6414	0.00141711	0.08576625	0.09132133	IB
[340,350)	344.999324	0.08934931	0.11854948	6315	0.00149181	0.08642536	0.09227325	IB
[350,360)	354.981727	0.08758615	0.11345714	6393	0.00141899	0.08480493	0.09036737	IB
[360,370)	364.964569	0.08702605	0.11096421	6298	0.00139824	0.0842855	0.0897666	IB
[370,380)	374.97864	0.08694064	0.11335264	6225	0.00143669	0.08412473	0.08975654	IB
[380,390)	384.995027	0.08524314	0.10975757	6368	0.00137541	0.08254733	0.08793895	IB
[390,400)	395.014272	0.08576667	0.11190541	6363	0.00140288	0.08301703	0.08851631	IB
[400,410)	405.055246	0.08738277	0.11097043	6407	0.00138637	0.08466548	0.09010006	IB
[410,420)	414.967104	0.08449053	0.10696607	6331	0.00134434	0.08185562	0.08712544	IB
[420,430)	425.055008	0.08391366	0.1083159	6456	0.00134806	0.08127146	0.08655587	IB
[430,440)	434.996696	0.08375339	0.10896646	6244	0.00137899	0.08105057	0.08645621	IB
[440,450)	445.002089	0.08450351	0.10844797	6268	0.0013698	0.08181871	0.08718832	IB
[450,460)	454.968697	0.08592365	0.11143449	6327	0.00140094	0.0831778	0.0886695	IB
[460,470)	465.01612	0.08528454	0.11017418	6137	0.00140638	0.08252804	0.08804104	IB
[470,480)	475.021582	0.08348296	0.10696002	6453	0.0013315	0.08087322	0.0860927	IB
[480,490)	484.976405	0.08429562	0.10955186	6464	0.0013626	0.08162492	0.08696632	IB
[490,500)	494.952878	0.08260045	0.10457131	6191	0.00132902	0.07999557	0.08520533	IB
[500,510)	504.969003	0.08535242	0.11007112	6260	0.00139119	0.08262569	0.08807915	IB
[510,520)	515.018332	0.08410827	0.1062195	6302	0.00133803	0.08148573	0.0867308	IB
[520,530)	525.026034	0.08172277	0.10902311	6310	0.00137247	0.07903272	0.08441282	IB
[530,540)	534.995277	0.08334404	0.10747562	6370	0.00134661	0.08070469	0.08598338	IB
[540,550)	545.031772	0.08358318	0.10855355	6275	0.00137037	0.08089726	0.0862691	IB
[550,560)	555.062757	0.08430273	0.10907634	6305	0.00137369	0.0816103	0.08699515	IB
[560,570)	564.969389	0.08214298	0.10432443	6356	0.00130856	0.0795782	0.08470776	IB
[570,580)	575.048173	0.08138127	0.10582796	6412	0.00132161	0.07879091	0.08397162	IB
[580,590)	584.995479	0.08323047	0.1057042	6293	0.00133249	0.08061879	0.08584214	IB
[590,600)	594.955856	0.08324857	0.10881858	6170	0.00138535	0.08053328	0.08596386	IB
[600,610)	604.997471	0.08267823	0.10809504	6370	0.00135437	0.08002368	0.08533279	IB
[610,620)	614.99544	0.08363726	0.10843995	6364	0.00135933	0.08097297	0.08630154	IB
[620,630)	625.044611	0.08213626	0.10643489	6324	0.00133841	0.07951298	0.08475954	IB
[630,640)	634.960751	0.07970737	0.10289072	6293	0.00129702	0.07716521	0.08224954	IB
[640,650)	645.020042	0.08202332	0.10571966	6304	0.00133152	0.07941354	0.0846331	IB
[650,660)	655.026561	0.08009982	0.10676599	6125	0.00136421	0.07742598	0.08277367	IB
[660,670)	665.035794	0.07982577	0.10331944	6340	0.00129759	0.0772825	0.08236905	IB

[670,680)	674.97157	0.0807137	0.10615054	6338	0.00133336	0.07810032	0.08332707	IB
[680,690)	684.998305	0.0808628	0.10446696	6325	0.00131356	0.07828823	0.08343737	IB
[690,700)	695.074434	0.07919984	0.10309077	6281	0.00130078	0.0766503	0.08174938	IB
[700,710)	704.978877	0.08242881	0.10656346	6153	0.00135852	0.07976612	0.08509151	IB
[710,720)	715.030849	0.08094076	0.10771246	6372	0.00134936	0.07829601	0.0835855	IB
[720,730)	725.001014	0.07984359	0.10555665	6189	0.00134176	0.07721374	0.08247344	IB
[730,740)	735.009691	0.08062557	0.10371331	6340	0.00130254	0.0780726	0.08317854	IB
[740,750)	744.964353	0.08163984	0.10442799	6172	0.00132924	0.07903452	0.08424515	IB
[750,760)	755.028216	0.07868375	0.10080412	6276	0.00127244	0.07618977	0.08117773	IB
[760,770)	764.993999	0.08028214	0.10540438	6213	0.00133724	0.07766115	0.08290312	IB
[770,780)	775.024326	0.07821783	0.10237597	6336	0.00128615	0.07569699	0.08073868	IB
[780,790)	785.040406	0.08052972	0.10398617	6328	0.0013072	0.07796761	0.08309184	IB
[790,800)	795.048359	0.07783766	0.10106016	6345	0.00126872	0.07535097	0.08032434	IB
[800,810)	805.033567	0.07934157	0.10330137	6269	0.00130469	0.07678438	0.08189876	IB
[810,820)	815.023125	0.07897544	0.1042574	6296	0.00131394	0.07640013	0.08155076	IB
[820,830)	825.022182	0.08084968	0.10442107	6191	0.00132711	0.07824854	0.08345082	IB
[830,840)	834.942124	0.0812091	0.10515087	6220	0.00133327	0.07859589	0.08382231	IB
[840,850)	845.047061	0.07697983	0.100682	6218	0.00127681	0.07447728	0.07948238	IB
[850,860)	854.973264	0.07744612	0.10012224	6335	0.00125793	0.07498058	0.07991167	IB
[860,870)	865.005295	0.07919144	0.10638862	6333	0.00133687	0.07657116	0.08181171	IB
[870,880)	875.047947	0.07763804	0.10209675	6242	0.00129226	0.07510521	0.08017087	IB
[880,890)	884.960799	0.0793277	0.10187666	6165	0.0012975	0.07678459	0.0818708	IB
[890,900)	894.994493	0.0774938	0.10192479	6321	0.001282	0.07498109	0.08000652	IB
[900,910)	905.017046	0.07832079	0.10531235	6219	0.00133542	0.07570336	0.08093822	IB
[910,920)	915.003906	0.0778634	0.10366652	6281	0.00130805	0.07529962	0.08042717	IB
[920,930)	925.039534	0.07752597	0.10155517	6157	0.00129425	0.07498924	0.08006269	IB
[930,940)	934.922054	0.07622764	0.09863865	6323	0.00124047	0.07379632	0.07865896	IB
[940,950)	945.038208	0.07799804	0.10393431	6251	0.00131457	0.07542148	0.0805746	IB
[950,960)	955.035504	0.07511456	0.0984292	6277	0.00124236	0.07267953	0.07754959	IB
[960,970)	965.021787	0.07904189	0.10170649	6305	0.00128087	0.07653138	0.0815524	IB
[970,980)	975.02675	0.07548583	0.09778107	6256	0.00123625	0.07306278	0.07790888	IB
[980,990)	984.987981	0.07626247	0.09784596	6159	0.00124677	0.07381879	0.07870614	IB
[990,1e+03]	995.018515	0.07648633	0.09856847	6251	0.0012467	0.07404279	0.07892987	IB
