# Supplementary material for: Genetic diversity, population structure, and combined detection of selection signatures in Iranian versus Afghan Baluchi sheep
Source: PLoS One. 2026 Jun 17;21(6):e0350262. doi: 10.1371/journal.pone.0350262 (PMC13274857; doi:10.1371/journal.pone.0350262)
Supplement: S3 Table — (PDF) [file pone.0350262.s006.pdf]

S3 Table. Significant selection signatures via Win5F_{ST} Method

CHR	Win5F_{ST}	Gene name	Pos1	Pos2	N-SNP
1	0.1768	CD84	1.1E+08	1.1E+08	1
1	0.1819	DAB1	31354718	32719832	2
1	0.178	FGF12	1.93E+08	1.94E+08	2
1	0.1663	PCOLCE2	2.44E+08	2.44E+08	1
1	0.1666	VANGL2	1.1E+08	1.1E+08	1
3	0.2349	LCLAT1	37167262	37358751	4
3	0.2349	LOC105613153	37189718	37198773	1
4	0.168	DFNA5	71022579	71142286	1
4	0.1796	DYNC1I1	12990529	13320436	2
4	0.1889	HDAC9	27257274	27354592	2
4	0.1644	OSBPL3	70824606	71020878	1
5	0.1733	SAFB	16231188	16292431	1
7	0.1721	FAM214A	54713621	54800027	1
8	0.1795	NKAIN2	13449754	14638114	1
9	0.1654	CRH	43349380	43585631	1
9	0.1649	CSMD3	63608188	65010296	1
11	0.1681	PCDH9	39644883	40823833	1
11	0.1677	ADPRM	29219273	29226481	1
11	0.1903	LOC105616352	35358660	35360399	1
11	0.1668	PIRT	29318817	29329234	1
12	0.1758	HMCN1	64798860	65338155	3
14	0.1716	GINS2	11135965	11202476	1
14	0.1864	IRF8	11330933	11348430	1
18	0.172	LOC105603115	24421003	24494825	1
18	0.164	RASGRF1	24244577	24346773	1
21	0.1648	LOC443348	38842888	38860901	1
21	0.1685	PAG6	38797291	38806782	1
22	0.1646	LOC105604206	2473889	2527424	1
25	0.1695	CTNNA3	22147066	24082789	1
25	0.186	ZNF365	18335412	18364098	2
27	0.1859	HAUS7	77914578	77939906	1
