# Supplementary material for: Genetic diversity, population structure, and combined detection of selection signatures in Iranian versus Afghan Baluchi sheep
Source: PLoS One. 2026 Jun 17;21(6):e0350262. doi: 10.1371/journal.pone.0350262 (PMC13274857; doi:10.1371/journal.pone.0350262)
Supplement: S4 Table — (PDF) [file pone.0350262.s007.pdf]

S4 Table. Significant genes of F_{ST} were associated with some QTL in sheep

CHR	GENE	Pos1	Pos2	QTL
1	CD84	109914308	109956058	BDENS/BFLUMB3/BONE_WT/BW/FA-C20:5/FA-C22:5/FATP/LMYP/MDLUMB3/MFPER/MUSWT/MY/PP/PUFA
1	DAB1	31354718	32719832	FATP/LMYP/MUSWT
1	FGF12	193316348	193556001	ASREP/BONE_WT/FA-C20:5/FA-C22:5/FATP/FLYD/LMYP/MDLUMB3/PUFA/SAOS
1	PCOLCE2	244192549	244276669	ASREP/FATP/FCURV/TFEC_1
1	VANGL2	109737700	109764829	BDENS/BFLUMB3/BONE_WT/BW/FA-C20:5/FA-C22:5/FATP/LMYP/MDLUMB3/MFPER/MUSWT/MY/PP/PUFA
3	LCLAT1	37167262	37358751	SL/TFEC_1
4	DFNA5	71022579	71142286	CVFD_PRI/BW/CVFD_PRI/HFEC
4	HDAC9	27257274	27354592	BW/CVFD_PRI/HFEC
4	OSBPL3	70824606	71020878	CVFD_PRI
5	SAFB	16231188	16292431	BW/FA-C16:1
7	FAM214A	54713621	54800027	CVFD_PRI/SL
8	NKAIN2	13449754	14638114	INTFAT/LATRICH_2
9	CRH	43349380	43585631	HCWT/LMA/MUSWT
9	CSMD3	63608188	65010296	HCWT/LMA/MUSWT
11	PCDH9	39644883	40823833	BONEP/FATP/FATWT/FECGEN/LMYP/TESTWT
11	ADPRM	29219273	29226481	BW/HCWT/INTFAT/JAWL/LATRICH_2/MPUFA/MY/MYPERS/PY
11	PIRT	29318817	29329234	BW/HCWT/INTFAT/JAWL/LATRICH_2/MPUFA/MY/MYPERS/PY
12	HMCN1	64798860	65338155	TFEC_1
14	GINS2	11135965	11202476	BONE_WT/DRESSING/FATWT/FECGEN/NFEC/TOTBONE
14	IRF8	11330933	11348430	BONE_WT/DRESSING/FATWT/FECGEN/NFEC/TOTBONE
18	RASGRF1	24244577	24346773	FA-C20:1/MY/SAOS/SL/TESTWT/WORMCT
21	PAG6	38797291	38806782	ADG/BW/FA-C14:0/FA-C16:0/FA-C18:1/FA-C18:2/FA-C18:3/FA-C20:1/FA-C20:4/FA-C22:5
25	CTNNA3	22147066	24082789	CVFD_PRI/MFDIAM/SL/TESTWT
25	ZNF365	18335412	18364098	CVFD_PRI/MFDIAM/SL/TESTWT/UYC
