# Supplementary material for: Genetic diversity, population structure, and combined detection of selection signatures in Iranian versus Afghan Baluchi sheep
Source: PLoS One. 2026 Jun 17;21(6):e0350262. doi: 10.1371/journal.pone.0350262 (PMC13274857; doi:10.1371/journal.pone.0350262)
Supplement: S5 Table — (PDF) [file pone.0350262.s008.pdf]

S5 Table. Significant genes of positive XP-EHH were associated with some QTL in sheep

CHR	GENE	Pos1	Pos2	N-SNP	QTL
1	FAF1	24699198	25184931	1	FATP/LMYP/MUSWT
1	LTN1	125347989	125405440	1	BDENS/BFLUMB3/BONE_WT/BW/FA-C20:5/FA-C22:5/FATP/FECGEN/LMYP/MDLUMB3/MUSWT/PUFA
1	ROBO2	142024870	143506267	1	BONE_WT/BONEP/FA-C20:5/FA-C22:5/FATP/LMYP/MUSWT/PUFA
1	TRABD2B	22043637	22261163	2	FATP/LMYP/MUSWT
3	AK8	3951920	4086483	1	HFEC/SL
3	KLHL29	31035923	31361641	1	HFEC/SL/TFEC_1
4	CCDC132	10482357	10619154	1	BW/CVFD_PRI/HFEC
4	RELN	44667117	45204871	1	CVFD_PRI
4	SEMA3C	39950466	40158909	1	BW/CVFD_PRI
6	KCNIP4	40155213	41461250	1	BW/FATP/FATWT/FECGEN/HCWT/LMYP/MFDIAM/MUSWT
7	DPH6	27830106	28024195	2	CVFD_PRI/LMA/SL
7	FAM81A	47816436	48397960	1	CVFD_PRI/HFEC/LMA/PP/SL
7	MYO1E	47971948	48188171	1	CVFD_PRI/HFEC/LMA/PP/SL
8	FILIP1	2032933	2306343	1	INTFAT/LATRICH_2
11	CEP112	61179107	61486465	3	MPUFA
11	CNTROB	27171693	27185700	1	BW/HCWT/INTFAT/LATRICH_2/MPUFA/MY/MYPERS/PY
11	DNAH2	26975834	27078157	1	BW/HCWT/INTFAT/LATRICH_2/MPUFA/MY/MYPERS/PY
11	FGF11	26746839	26753373	1	BW/HCWT/INTFAT/LATRICH_2/MPUFA/MY/MYPERS/PY
11	HELZ	62021111	62172105	1	MPUFA
11	KDM6B	27083889	27101827	1	BW/HCWT/INTFAT/LATRICH_2/MPUFA/MY/MYPERS/PY
12	AURKAIP1	49216627	49222860	1	FATP/LMYP
12	DVL1	49251681	49263814	1	FATP/LMYP
12	MROH9	36648075	36749324	4	BDENS/BDENS/FATP/LMYP/MY
16	ADCY2	65279515	65721177	4	DRESSING/LMYP/SCFT
19	CACNA2D2	49822781	49940140	1	DRESSING
19	CHL1	25920304	26271658	1	DRESSING
19	CNTN4	23123970	24164718	2	DRESSING
21	PPP6R3	45241445	45335197	1	FA-C14:0/FA-C16:0/FA-C18:1/FA-C18:2/FA-C18:3/FA-C20:1/FA-C20:4/FA-C22:5
21	SYT12	44302869	44321265	1	FA-C14:0/FA-C16:0/FA-C18:1/FA-C18:2/FA-C18:3/FA-C20:1/FA-C20:4/FA-C22:5
22	MMS19	17890082	17924091	1	SCS
