# Supplementary material for: Genetic diversity, population structure, and combined detection of selection signatures in Iranian versus Afghan Baluchi sheep
Source: PLoS One. 2026 Jun 17;21(6):e0350262. doi: 10.1371/journal.pone.0350262 (PMC13274857; doi:10.1371/journal.pone.0350262)
Supplement: S6 Table — (PDF) [file pone.0350262.s009.pdf]

S6 Table. Significant genes of negative XP-EHH were associated with some QTL in sheep

CHR	GENE	Pos1	Pos2	N-SNP	QTL
1	GRIK3	11406516	11651711	1	LMYP/MUSWT
1	HDAC4	2016417	2200867	2	MUSWT
1	HDLBP	433270	509476	1	MUSWT
1	LRRFIP1	3178226	3280872	2	MUSWT
1	NDUFA10	1485019	1522935	1	MUSWT
1	PPP1R7	528053	544659	1	MUSWT
1	THAP4	211316	249288	1	MUSWT
1	TRAF3IP1	2734273	2793481	1	MUSWT
2	BICD2	28472925	28503891	1	FA-C18:3/FA-C20:4/FA-C20:5/FA-C22:5/MFPER
2	ERCC6L2	30052033	30205680	1	FA-C18:3/FA-C20:4/FA-C20:5/FA-C22:5/MFPER
2	FGD3	28348926	28392280	1	FA-C18:3/FA-C20:4/FA-C20:5/FA-C22:5/MFPER
2	IPPK	28514163	28574528	1	FA-C18:3/FA-C20:4/FA-C20:5/FA-C22:5/MFPER
2	PHF2	27764305	27852487	2	FA-C18:3/FA-C20:4/FA-C20:5/FA-C22:5/MFPER
2	SYK	25652944	25758231	1	FA-C18:3/FA-C20:4/FA-C20:5/FA-C22:5/MFPER
2	WNK2	28059435	28213714	2	FA-C18:3/FA-C20:4/FA-C20:5/FA-C22:5/MFPER
3	NCOA1	31927887	32146995	1	HFEC/SL/TFEC_1
4	COG5	48317874	48588268	3	CVFD_PRI
4	ELMO1	59884413	60467506	1	CVFD_PRI
4	PRKAR2B	48166894	48277838	1	CVFD_PRI
5	DMXL1	32126147	32258823	1	BW/FA-C16:1/IOA/Stature
7	GALNT16	77966960	78076584	2	BDENS/CVFD_PRI/SL
7	RGS6	80373231	81056524	3	BDENS/CVFD_PRI/SL
7	SUPT16H	23005323	23041283	1	CVFD_PRI/LMA/SL
8	MTHFD1L	74602149	74780305	2	FECGEN/INTFAT/LATRICH_2
16	CAPSL	38140289	38177428	1	BW/DRESSING/LMYP/RLEGS/SCFA/SCFT
16	IL7R	38208728	38242386	1	BW/DRESSING/LMYP/RLEGS/SCFA/SCFT
26	KCNU1	30764780	30907477	2	MUSWT/Stature/UDDATT/WORMCT
26	TENM3	9763218	12531041	2	MUSWT
