# Supplementary material for: Genetic diversity, population structure, and combined detection of selection signatures in Iranian versus Afghan Baluchi sheep
Source: PLoS One. 2026 Jun 17;21(6):e0350262. doi: 10.1371/journal.pone.0350262 (PMC13274857; doi:10.1371/journal.pone.0350262)
Supplement: Attachment — Submitted filename: Response to Reviewers.docx [file pone.0350262.s013.docx]

**Dear Editorial Manager**

**Warm Greeting**

Thank you for allowing us to submit a revised draft of the manuscript "Genetic diversity, population structure, and combined detection of selection signatures in Iranian versus Afghan Baluchi sheep" for publication in the PLOS ONE Journal. We appreciate the time and effort you and the reviewers dedicated to providing feedback on our manuscript and are grateful for the insightful comments and valuable improvements to our paper. We have incorporated most of the suggestions made by the reviewers. These changes are in red font in the manuscript. You may also find below a point-by-point response to the reviewers’ comments and concerns in blue.

**Reviewers' Comments to the Authors:** I have separated my responses to the reviewers’ comments into several categories to achieve an integrated approach.

**Editor in Chief**

1. Please ensure that your manuscript meets PLOS ONE's style requirements, including those for file naming. The PLOS ONE style templates can be found at https://journals.plos.org/plosone/s/file?id=wjVg/PLOSOne_formatting_sample_main_body.pdf and https://journals.plos.org/plosone/s/file?id=ba62/PLOSOne_formatting_sample_title_authors_affiliations.pdf

Author response: Thank you for pointing this out. We have carefully revised the manuscript and supporting information files to ensure they adhere to PLOS ONE’s style requirements, including formatting and file naming conventions, according to the official templates and recent published papers.

2. Please note that PLOS One has specific guidelines on code sharing for submissions in which author-generated code underpins the findings in the manuscript. In these cases, we expect all author-generated code to be made available without restrictions upon publication of the work. Please review our guidelines at https://journals.plos.org/plosone/s/materials-and-software-sharing#loc-sharing-code and ensure that your code is shared in a way that follows best practice and facilitates reproducibility and reuse.

Author response: We thank the reviewer for highlighting the PLOS ONE code-sharing guidelines. All author-generated code underlying the key findings of this manuscript, including quality control (PLINK), population structure (PCA, Admixture), genetic diversity metrics, LD decay, Ne estimation (SNeP), F_ST_, and XP-EHH (rehh) analyses, has been consolidated into a single, comprehensive supplementary file (S1 File) and made openly available without restrictions.

3. Your ethics statement should only appear in the Methods section of your manuscript. If your ethics statement is written in any section besides the Methods, please delete it from any other section.

Author response: We appreciate the editor’s guidance. We have carefully checked the manuscript and confirm that the ethics statement is present only in the Methods section and did not appear elsewhere; therefore, no revisions were required regarding this point.

4. Please note that your Data Availability Statement is currently missing the DOI/accession number of each dataset OR a direct link to access each database. If your manuscript is accepted for publication, you will be asked to provide these details on a very short timeline. We therefore suggest that you provide this information now, though we will not hold up the peer review process if you are unable.

Author response: After negotiating with all authors, we decided to upload our data file as supporting information. Hope it helps.

5. Please upload a new copy of Figure 2 as the details are not clear. Please follow the link for more information: <https://journals.plos.org/plosone/s/figures>

Author response: A new, higher-quality version of Figure 2 has been prepared according to the PLOS ONE figure guidelines and has been uploaded to replace the previous version.

6. If the reviewer comments include a recommendation to cite specific previously published works, please review and evaluate these publications to determine whether they are relevant and should be cited. There is no requirement to cite these works unless the editor has indicated otherwise.

Author response: We appreciate the editor’s guidance. We have evaluated all reviewer-recommended references and incorporated the ones that are relevant to our study into the revised manuscript, where appropriate.

**Reviewer 1**

1. Sample size imbalance (IB n=86 vs AB n=15) and statistical power The Afghan Baluchi population is represented by only 15 animals, while the Iranian sample includes 86. This imbalance substantially affects:

- allele frequency estimates (critical for FST),

- haplotype-based statistics (XP-EHH),

- LD estimation,

- and effective population size (Ne) inference.

The manuscript does not sufficiently discuss how these methodological limitations may influence the results, particularly for the AB population. The small sample size may also inflate apparent within-population diversity or artificially alter LD decay patterns. In particular, the manuscript lacks adequate detail on how the two sample sets represent their respective populations. The sample size for AB is too small to be considered representative of a population. Although the IB sample size is large enough to represent the flock from which it was drawn, it cannot be assumed to represent the wider population. The authors also claim that the sampled animals are unrelated, but they do not explain how 86 animals from a single flock, bred for many decades, can be unrelated. It is unclear how many generations of pedigree data were examined to assess relatedness.

Recommendation: Please add a detailed discussion of the limitations introduced by the sample sizes, especially with respect to FST variance, XP-EHH power, and Ne precision. This is crucial for interpreting the robustness of the AB results. It should also clearly demonstrate the IB samples are form a single flock at a breeding station which may not represent a much larger population in a vast geographical region. The authors should indicate the methodology they used to assess the relationship between sampled animals.

Author response: We thank the reviewer for their insightful comments regarding sample size imbalance and population representation. We fully acknowledge the limitations introduced by the smaller AB sample size (n=15) compared to IB (n=86), which can increase variance in allele frequency estimates, FST, XP-EHH power, LD patterns, and Ne precision, particularly for the AB population. These limitations have now been explicitly discussed in a new paragraph in the Discussion section (lines 413-429).

Regarding population representation, we clarify that the IB samples originate from a single high-purity breeding station flock, which was specifically selected due to the lack of purity information for Baluchi populations scattered across eastern and southern regions of Iran outside the station. These field populations are typically not maintained as purebreds, making the station flock the most reliable source for a representative and pure IB genetics. We now emphasize that while the IB sample (n=86) adequately represents this managed flock, it may not fully capture the broader geographic diversity of Iranian Baluchi sheep (Methods, lines 153-161).

For the Afghan Baluchi (AB) samples, we acknowledge that n=15 is exploratory due to logistical challenges: unlike Iran, Afghanistan lacks dedicated Baluchi breeding centers, and these sheep are raised traditionally in mixed flocks. Identifying and collecting additional purebred AB samples proved infeasible within the scope of this study. The AB samples were collected from multiple regions in Herat province, minimizing relatedness.

To address relatedness concerns, we performed IBD analysis using PLINK (PI_HAT). The AB samples showed very low PI-HAT values due to sampling from diverse regions. For IB samples, only one pair exceeded 0.5% PI-HAT; we removed one individual from this pair and conducted sensitivity analyses, which showed no substantial changes in results (Methods, lines 173-177).

To address statistical concerns, we used the θ estimator for F_ST_, which is robust to sample size imbalances and reduces bias from unequal allele frequency variance [ref: Weir & Cockerham 1984; Bhatia et al. 2013]. Similar sample sizes have been successfully applied for XP-EHH, LD decay, and Ne estimation in sheep population studies [Jin et al., 2024; Zhu et al., 2023; Khalkhali-Evrigh et al., 2025]. Despite these limitations, the consistent selection signals across methods suggest robust patterns warranting further investigation with larger samples.

2. Selection thresholds and multiple testing Significance thresholds appear to be defined as the top 1% for Win5FST and |XP-EHH| ≥ 3 for XP-EHH. However:

• The rationale for these choices is not clearly presented.

• No multiple testing correction is attempted or discussed.

• Because of the small AB sample, false positives are likely.

Recommendation: Provide justification for the chosen cutoffs (e.g., empirical distributions, commonly used thresholds in the literature). Acknowledge that these represent heuristic thresholds and may include false positives.

Author response: We thank the reviewer for raising this important point regarding selection thresholds and multiple testing. We have now clarified the rationale for our thresholds in the revised Methods section (lines 246-248) and acknowledged their heuristic nature in the Discussion (lines 487-491).

The top 1% threshold for Win5FST and |XP-EHH| ≥ 3 were selected following commonly used empirical cutoffs in sheep and livestock selection signature studies [Demissie et al., 2025; Abied et al., 2020; Zhang et al., 2022]. These thresholds effectively identify extreme outliers in the empirical genomic distribution, which is the standard exploratory approach for detecting candidate selection regions when prior biological knowledge is limited.

Regarding multiple testing correction, we acknowledge that formal corrections (e.g., Bonferroni) are rarely applied in selection signature scans due to: (1) high linkage disequilibrium causing marker non-independence, (2) the exploratory nature of these analyses aiming to generate hypotheses rather than test them definitively, and (3) substantial power loss from overly conservative adjustments [Sabeti et al. 2007; Vitti et al. 2013]. Similar top 1% and XP-EHH thresholds without multiple testing correction are routinely reported across recent livestock genomic selection studies [Lei et al., 2021; Zhang et al., 2023]. We now explicitly state that these represent heuristic thresholds likely to include false positives, particularly given the small AB sample size, and emphasize that identified regions require experimental validation.

3. Interpretation of Ne and LD results. The manuscript states that IB has slightly lower diversity but a higher recent effective population size compared to AB. This pattern is counterintuitive and requires additional explanation.

Recommendation: Expand the discussion of how LD-based Ne behaves under selection pressure, differences in sample sizes, and differences in historical demography. Clarify whether differences in SNP retention after QC affected estimates in either group.

Author response: We thank the reviewer for highlighting this important point regarding the seemingly counterintuitive Ne and LD patterns between IB and AB populations. We have expanded the Discussion with a dedicated paragraph addressing these factors (lines 449-459). “The apparently higher recent Ne in IB despite lower genetic diversity reflects breeding station management practices and sample size effects on LD-based estimation, rather than true population-level differences. These patterns highlight the importance of interpreting station vs. field population estimates cautiously. Also, QC filtering was identical across populations (same --geno, --maf, --mind, --hwe thresholds), so SNP retention did not differentially affect estimates.

4. Overinterpretation of candidate genes and QTL overlaps. Several candidate genes are linked to long lists of traits (e.g., growth, carcass traits, milk production, reproduction, immune traits, high-altitude adaptation, follicle development, etc.). While these associations may be reported in the literature, not all are relevant to the biological context of Baluchi sheep.

Recommendation: Please moderate these sections by:

• presenting these genes as “putative candidates”,

• focusing on the traits that are biologically plausible in Baluchi populations,

• reducing the length of gene-by-gene literature reviews,

• and adding a statement acknowledging the limitations of inferring function from QTL overlap.

Author response: We agree with the reviewer that some of the original descriptions of candidate genes and QTL overlaps were overly extensive and may have suggested stronger functional evidence than is warranted. In the revised manuscript, we now consistently refer to these loci as “putative candidate genes”, have shortened the gene‑by‑gene literature descriptions, and focused our discussion on traits that are biologically plausible and relevant in Baluchi sheep (e.g. growth, fat deposition, adaptation to arid environments, reproduction and disease resistance). We have also added an explicit statement in the Discussion acknowledging the limitations of inferring gene function solely from positional overlap with broad QTL or association regions and emphasizing that our interpretations are hypothesis‑generating rather than causal.

5. Novelty and relation to previous work. Some earlier studies of sheep in the region (e.g., Eydivandi et al. 2021; Barani et al. 2023; Taheri et al. 2024) have already identified selection signatures in Baluchi sheep. Recommendation: Clarify more explicitly what novel insight this manuscript contributes beyond earlier work, for example, whether the novelty lies in the direct comparison of IB and AB or in the combined use of FST, XP-EHH, and QTL integration.

Author response: We appreciate the reviewer’s comment and agree that several previous studies have investigated selection signatures in Baluchi or regional sheep populations (e.g. Eydivandi et al. 2021; Barani et al. 2023). However, those studies mainly compared Baluchi with multiple distinct breeds from Iran, Afghanistan or neighboring countries at a broad inter‑breed level, without a focused genomic comparison between Iranian Baluchi (IB) and Afghan Baluchi (AB), which are believed to share a common ancestral background. In contrast, the novelty of our work lies in (i) a dedicated, pairwise comparison of IB and AB populations to dissect how historical management and breeding in the two countries have shaped divergent selection signatures within the same breed, and (ii) the combined use of complementary statistics (F_ST_ and XP‑EHH) together with systematic integration of sheep QTL information, which allows us to link shared and population‑specific selective sweeps to concrete production, adaptation and reproduction traits and to highlight candidate regions for future functional and genomic selection studies.

6. English language and clarity. There are numerous grammatical errors, missing words, and awkward phrasings throughout the manuscript (examples include missing subjects, such as in “To assess genetic diversity… calculated several metrics” or inconsistent verb forms). I strongly recommend that the entire manuscript be revised by a fluent English speaker or a professional editing service.

Author response: Thank the reviewer for highlighting the language issues. We have thoroughly revised the entire manuscript for grammatical accuracy, clarity, and fluency. All changes enhance readability while preserving the original scientific meaning. We believe the manuscript now meets high standards of English proficiency.

7. Methods clarity

• The description of the Win5FST approach should indicate whether sliding windows overlap and how they were defined (by 5 SNPs rather than a fixed BP window).

• LD analysis: specify exact distance intervals used for r² vs distance curves.

• ADMIXTURE: include the sample size for each group in the figure or caption.

Author response: Thank the reviewer for these suggestions to improve methodological transparency. All requested clarifications have been incorporated as detailed below, with changes highlighted in the revised manuscript.

Win5FST approach: The Win5FST approach indicates overlap windows by 5 adjacent SNPs rather than a fixed BP window, with one SNP moving forward in the sequence. This is added to the Methods section (lines209-210).

LD analysis: Pairwise r² values were grouped into consecutive 10‑kb non-overlapping physical distance bins from 0 to 1 Mb (0–10 kb, 10–20 kb, …, 990–1000 kb). For each distance bin, we calculated the mean r², its standard error, and the corresponding 95% confidence interval across all SNP pairs; the mean r² values were plotted against the midpoint of each distance bin, with shaded 95% confidence ribbons, to visualize LD decay patterns in each population (lines 213-217).

ADMIXTURE: Sample sizes for each group (AB=15, IB=86) have been added to the Fig2 and figure caption (Fig 2, caption updated and highlighted, lines=277-278).

8. Figures and tables

• PCA and ADMIXTURE figures should include clearer legends and axes.

• Supplementary tables (S2–S5) should include chromosome positions, SNP counts, and window sizes.

Author response: All specified revisions have been implemented as follows, with changes highlighted in the revised manuscript.

PCA and ADMIXTURE figures: Legends have been expanded for clarity, and axes now include explicit labels. See updated Fig 1 and Fig 2.

Supplementary tables S2–S5: Columns for chromosome positions, SNP counts per window, and window sizes have been added to each table. These details are now included (S2 Table and S3 Table).

These modifications improve interpretability and data accessibility while preserving original findings.

9. Terminology consistency

The manuscript alternates between “breed”, “population”, and “race.” Please standardize terminology

Author response: We thank the reviewer for noting the need for uniform terminology. The manuscript has been thoroughly reviewed, and all instances of "breed," "population," and "race" have been standardized to "population" throughout the text for consistency. Because we believe that both populations belong to the same breed, we used the word "population". These changes are highlighted across the entire manuscript.

10. Ethics and ARRIVE guidelines

Because the genotypes were obtained from previously published studies, clarify whether the ARRIVE guidelines apply to the original sampling or to the present analysis.

Author response: Since all genotypes were sourced from previously published studies and re-analyzed in the present work, the ARRIVE guidelines apply to the original sampling protocols rather than our secondary genomic analyses.

This has been explicitly clarified in the Ethics statement (lines 141-145): "Genotypes were obtained from previously published studies where all procedures were conducted in strict accordance with relevant guidelines and regulations, including the ARRIVE guidelines (Animal Research: Reporting of In Vivo Experiments). Our re-analysis of these public datasets followed standard bioinformatics practices."

**Reviewer 2**

1. Highlight the following in the abstract: Sample size imbalance (IB n=86 vs. AB n=15) reduces power and may bias structure and selection scans.
2. Signals were defined by top 1% thresholds (and XP-EHH ±3) without genome-wide multiple-testing correction; results require validation.
3. Use of the Ovine 50K array can introduce ascertainment bias; analyses relied on 38,193 shared SNPs, which may miss population-specific variants.

Author response: Thanks for your comment. The following sentence has been added immediately after the genotyping description in the abstract: "Note that the sample size imbalance (IB n=86 vs. AB n=15) may reduce statistical power and potentially bias population structure and selection scan results." (Lines 39-41)

The following sentence has been added to the abstract after the selection signature results: "Selection signals were identified using top 1% FST thresholds and XP-EHH without genome-wide multiple-testing correction; results require experimental validation." (Lines 59-61).

The following sentence has been added to the abstract immediately after the sample size statement: "Additionally, use of the Ovine 50K array may introduce ascertainment bias; analyses were based on 38,193 shared SNPs, potentially missing population-specific variants." (Lines 41-43).

4. State how unrelatedness was verified (e.g., KING/IBD thresholds), not just asserted as “unrelated.” Provide counts of animals and SNPs removed at each QC step per population (not just thresholds).

Author response: Thanks to the reviewer for requesting detailed QC documentation. As reviewer # one also suggested, we did verify unrelatedness using PLINK 1.9's IBD test with PI_HAT > 0.5 threshold to identify first-degree relatives. In the Iranian Baluchi population, one pair exceeded this threshold; one individual was removed, with sensitivity analysis confirming made no difference to the results (Methods, lines 173-177).

Detailed QC steps with per-population removal counts (animals and SNPs) have been added to the existing QC table (S1 Table).

5. Confirm genome assembly build used (Oar_v4.0 vs. ARS-UI_Ramb_v2.0, etc.) and SNP coordinates mapping.

Author response: The genome assembly used has been explicitly confirmed in the Methods section: "Genotyping was performed by Ovine SNP50K Beadchip array (Illumina Oar_v4.0 assembly) in both groups, with all SNP coordinates mapped to this reference genome." (lines 167-168).

6. Describe phasing/imputation pipeline used for XP‑EHH (e.g., BEAGLE/SHAPEIT parameters, reference panel if any); XP‑EHH requires phased haplotypes—this is not currently described.

Author response: XP-EHH indeed requires phased haplotypes, which were present in our input VCF files. No separate imputation/phasing pipeline (e.g., BEAGLE/SHAPEIT) was applied, as the VCFs already contained phased genotype data. The following paragraph has been added to the Materials and Methods section (lines 225-237):

"Before XP-EHH analysis, genomic VCF files for each population were filtered for high-quality SNPs (MAF > 0.01) and confirmed to contain phased haplotypes. No additional imputation or phasing was performed, as the input VCFs were derived from pre-phased sequencing data. For each chromosome, haplotypes were converted to haplohh objects using the data2haplohh() function from the rehh v3.2.2 R package with parameters: min_maf=0.01, allele_coding="01", polarize_vcf=FALSE, and remove_multiple_markers=TRUE. EHH scans were then performed using scan_hh() with limhaplo=5, limehh=0.05, limehhs=0.05, phased=TRUE, and polarized=TRUE. Finally, XP-EHH scores were calculated with ies2xpehh(standardize=TRUE)."

7. Provide ADMIXTURE cross-validation details (folds, random seed).

Author response: ADMIXTURE cross-validation details (5-fold CV, default seed) have been added to the Methods section (lines 184-187).

8. Define distance binning and how standard errors across bins were computed (and will be plotted).

Author response: Distance binning is defined as 10 non-overlapping physical distance bins (<0.1 Mb, 0.1-0.2 Mb, ..., 0.9-1.0 Mb). Mean r² calculated across all SNP pairs per bin and plotted against bin midpoints (lines 205-211).

9. XP‑EHH: detail normalization (per-chromosome or genome-wide), p-value computation from standardized scores, and how significant regions were called (beyond |Z|>3).

Author response: XP-EHH details added: per-chromosome Z-score normalization [ies2xpehh(standardize=TRUE)], p-values from standard normal distribution, significant regions as top 1% outliers (|Z|>3.09) (lines 237-244).

10. For GO/KEGG, report multiple-testing correction method (e.g., BH-FDR) and cutoff, not just nominal P-values.

Author response: GO/KEGG multiple-testing correction details added: DAVID v6.8 with Bonferroni correction, adjusted p < 0.05 considered significant (lines 250-251).

11. Full software versions and environment are required. Already partially listed: PLINK v1.9, ADMIXTURE v1.3.0, SNeP v1.1, rehh (no version), R packages (ggfortify, pheatmap). Add: OS, R version, package versions, command flags, seeds.

Author response: Full software versions added throughout Methods. All analyses were performed on a Linux (Ubuntu 20.04) high-performance computing cluster (Supplementary Table S1). Command flags/seeds specified for ADMIXTURE (--cv), XP-EHH (standardize=TRUE), etc."

12. Explicitly address the small Afghan sample size: Power limitations and higher variance in AB estimates (n=15), potential bias in ADMIXTURE, FST, and XP‑EHH, and risk of false negatives in AB.

Author response: Small Afghan sample size (n=15) limitations expanded in Discussion (lines 397-413): explicitly addresses power limitations, higher AB variance, ADMIXTURE/FST/XP-EHH bias risks, false negative potential, and mitigation via empirical thresholds + multi-method validation.

13. The possibility that some signals reflect drift and demographic history rather than selection; interpret candidate gene links with caution and frame them as hypotheses for validation.

Author response: Drift vs. selection caution added to Conclusions (lines 579-581): 'Some signals may reflect genetic drift/demographic history rather than selection. Candidate genes framed as hypotheses requiring validation.

14. PCA: add % variance on axes (already in text) and 95% confidence ellipses by population; include LD-pruning note in legend.

Author response: PCA Fig 1 and Methods updated: 1) % variance explicitly shown on axes (PC1 18.07%, PC2 17.58%), 2) 95% confidence ellipses added per population, 3) legend clarified (genome-wide SNPs, no LD-pruning). Methods: 'PCA performed on all SNPs using PLINK v1.9' (lines 179-181)."

15. Heatmap/NJ tree: include bootstrap support for key nodes; specify distance metric and clustering method in caption.

Author response: Heatmap/NJ tree Methods and captions updated with full details (lines 188-192, Fig 2):

16. LD decay: plot mean r2 per bin with ribbons showing SE or 95% CI; report the number of pairwise comparisons per bin.

Author response: Thanks to the reviewer for this helpful suggestion. We have now summarized LD decay as the mean r² per 10‑kb non‑overlapping physical distance bin from 0 to 1 Mb and plotted these values against the midpoint of each bin. For each bin, we additionally calculated the standard error of r² and the corresponding 95% confidence interval across all pairwise SNP comparisons; these are shown as shaded ribbons around the mean r² curves in the revised LD decay figure. We also report, in the Results/Methods, the number of pairwise SNP comparisons per distance bin for each population (lines 212-217, Fig 3).

17. Ne trajectories: include shaded 95% CIs (e.g., from bootstrap over SNP blocks) and note SNeP assumptions in legend.

Author response: We thank the reviewer for this helpful suggestion. We have now added shaded 95% CI around the Ne trajectories for both populations. The confidence intervals were obtained using a block‑bootstrap procedure over LD bins (weighted resampling by the number of SNP pairs per bin, ‘items’ in the SNeP output), and the resulting 2.5th and 97.5th percentiles across 1,000 bootstrap replicates are shown as shaded areas around the curves (lines 206-210, Fig 3).

18. Manhattan (FST, XP‑EHH): add multiple-testing thresholds (e.g., FDR line) and include QQ plots for each scan in the Supplementary to assess inflation; annotate top loci with gene names.

Author response: For both F_ST_ and XP-EHH scans, we used genome-wide empirical thresholds based on the upper 1% of the statistical distribution (Win5FST and |XP-EHH| ≥ 3), which follows common practice in LD-associated marker selection scans. Because these statistics were interpreted in terms of empirical quantiles rather than analytically derived p-values, standard QQ plots of p-values (as in GWAS) are not directly applicable. Instead, we assessed potential inflation by examining the genome-wide distribution of standard XP-EHH scores using conservative upper 1% thresholds and now represent these thresholds as horizontal lines in Manhattan plots, and plot the genes closest to the outliers of the Ker annotation loci.

References

Jin M, Wang H, Liu G, Lu J, Yuan Z, Li T, Liu E, Lu Z, Du L, Wei C. Whole-genome resequencing of Chinese indigenous sheep provides insight into the genetic basis underlying climate adaptation. Genetics Selection Evolution. 2024;56(1):26.

Zhu M, Yang Y, Yang H, Zhao Z, Zhang H, Blair HT, Zheng W, Wang M, Fang C, Yu Q, Zhou H. Whole-genome resequencing of the native sheep provides insights into the microevolution and identifies genes associated with reproduction traits. BMC genomics. 2023;24(1):392.

Khalkhali-Evrigh R, Hedayat N, Seyedsharifi R, Shakouri M, Ponnampalam EN. Genomic evidence of improved fertility and adaptation in Iranian domestic sheep attributed to introgression from Asiatic Mouflon and urial. Scientific Reports. 2025;15(1):1185.

Bhatia G, Patterson N, Sankararaman S, Price AL. Estimating and interpreting FST: the impact of rare variants. Genome research. 2013;23(9):1514-21.

Demissie BE, Tarekegn GM, Dadi H, Edea Z, Woldesemayat AA, Kim KS, et al. Genomic Signatures of Positive Selection and Local Adaptation in Ethiopian Sheep Populations. Trop. Anim. Sci. J. 2025;48(3):179-88.

Abied A, Bagadi A, Bordbar F, Pu Y, Augustino SM, Xue X, et al. Genomic diversity, population structure, and signature of selection in five Chinese native sheep breeds adapted to extreme environments. Genes. 2020;11(5):494.

Zhang CL, Liu C, Zhang J, Zheng L, Chang Q, Cui Z, et al. Analysis on the desert adaptability of indigenous sheep in the southern edge of Taklimakan Desert. Sci. Rep. 2022;12(1):12264.

Vitti JJ, Grossman SR, Sabeti PC. Detecting natural selection in genomic data. Annual review of genetics. 2013 Nov 23;47(1):97-120.

Lei Z, Sun W, Guo T, Li J, Zhu S, Lu Z, Qiao G, Han M, Zhao H, Yang B, Zhang L. Genome-wide selective signatures reveal candidate genes associated with hair follicle development and wool shedding in sheep. Genes. 2021 Nov 29;12(12):1924.

Zhang W, Jin M, Li T, Lu Z, Wang H, Yuan Z, Wei C. Whole-genome resequencing reveals Selection Signal related to Sheep wool fineness. Animals. 2023 Sep 16;13(18):2944.
