# Supplementary material for: Genetic diversity, population structure, and combined detection of selection signatures in Iranian versus Afghan Baluchi sheep
Source: PLoS One. 2026 Jun 17;21(6):e0350262. doi: 10.1371/journal.pone.0350262 (PMC13274857; doi:10.1371/journal.pone.0350262)
Supplement: Attachment — Submitted filename: Response_to_Reviewers_auresp_2.docx [file pone.0350262.s014.docx]

**Dear Editorial Manager**

**Warm Greeting**

Thank you for following up this revision and we appreciate the time and effort you and the reviewers spent to provide comments on this manuscript. All of comments were very positive and we have applied all of the suggestions made by the reviewers. These changes are highlighted in yellow in the manuscript.

**Journal Requirements:**

Please ensure that your manuscript meets PLOS ONE's style requirements, including those for file naming. The PLOS ONE style templates can be found at https://journals.plos.org/plosone/s/file?id=wjVg/PLOSOne_formatting_sample_main_body.pdf and https://journals.plos.org/plosone/s/file?id=ba62/PLOSOne_formatting_sample_title_authors_affiliations.pdf

Author response: Thank you. All of citations are double checked now.

**Review Comments to the Author**

Reviewer #2: This manuscript presents a well‑executed and meaningful investigation into genetic diversity, population structure, and selection signatures in Iranian and Afghan Baluchi sheep, using robust genomic tools and clear analytical methods. The identification of candidate genes linked to economically important traits is especially valuable and demonstrates both scientific rigor and practical relevance, highlighting the strength and coherence of the study’s design. Overall, the work is well‑structured, clearly written, and provides insights that make it suitable for publication. The current draft has addressed the necessary editorial refinements, and I recommend acceptance for publication.

Author response: Thanks a lot.

Reviewer #3: This is an interesting study which investigates the genetic diversity and population structure and notable genetic signatures in two populations of native breed sheep- the Baluchi breed from Iran and Afghanistan.

Having not reviewed the original, none of the comments were mine, but appear to have been addressed. In the main, most of the comments below are just around polishing of the manuscript. I would also suggest a final proof read before re-submission.

Line 65-66-this doesn’t makes sense, please reword

Line 78- worth defining IB and AB in the main text (appreciate that they are defined in the abstract)

Line 90-93- this is a bit of a repeat of what you have above, maybe worth considering consolidating some of this with the bits above

Line 177-178- this is not a complete sentence

Line 200-201- this is not a complete sentence

Table 3 has a large legend with all the abbreviations in, whereas table 2 does not. Can the same be added for table 2 please

Line 566- ‘with the results of the current study’ (add in ‘the’)

Line 608- capital letter needed for Putative

Author response: We appreciate the reviewer’s comments. We have corrected all suggestions. Regarding Tables 2 and 3, I have checked and font size in these two tables are exactly the same. Therefore I can not change one based on another.

Reviewer #4: The authors have adequately addressed the main points raised in the initial review.

Limitations related to sample size imbalance, statistical power, and LD based Ne estimates are now clearly acknowledged and appropriately discussed.

The interpretation of selection signatures and candidate genes has been substantially moderated and framed as exploratory.

In the Conclusions, it would be appropriate to explicitly state: “These results should be interpreted as reflecting differences between a managed breeding population and a traditional field population rather than national level divergence.”

Overall, the manuscript is technically sound and suitable for publication after minor editorial polishing.

Author response: We thank the reviewer for raising this important point regarding conclusion. This statement has been added now.

**Best regards,**

**Ali Javadmanesh, PhD**

**Associate Professor**

**Department of Animal Science,**

**Faculty of Agriculture,**

**Ferdowsi University of Mashhad**
