# Supplementary material for: Genetic diversity, population structure, and combined detection of selection signatures in Iranian versus Afghan Baluchi sheep
Source: PLoS One. 2026 Jun 17;21(6):e0350262. doi: 10.1371/journal.pone.0350262 (PMC13274857; doi:10.1371/journal.pone.0350262)
Supplement: Peer review history

# Peer review: Decision Letter 0

**DOI:** 10.1371/journal.pone.0350262.r001

**Authors:** Muniyandi Nagarajan

8 Dec 2025

PONE-D-25-52267

Genetic diversity, population structure, and combined detection of selection signatures in Iranian versus Afghan Baluchi sheep

PLOS ONE

Dear Dr. Javadmanesh,

Thank you for submitting your manuscript to PLOS ONE. After careful consideration, we feel that it has merit but does not fully meet PLOS ONE’s publication criteria as it currently stands. Therefore, we invite you to submit a revised version of the manuscript that addresses the points raised during the review process.

Please submit your revised manuscript by Jan 22 2026 11:59PM. If you will need more time than this to complete your revisions, please reply to this message or contact the journal office at plosone@plos.org. When you're ready to submit your revision, log on to https://www.editorialmanager.com/pone/ and select the 'Submissions Needing Revision' folder to locate your manuscript file.

Please include the following items when submitting your revised manuscript:

- A rebuttal letter that responds to each point raised by the academic editor and reviewer(s). You should upload this letter as a separate file labeled 'Response to Reviewers'.
- A marked-up copy of your manuscript that highlights changes made to the original version. You should upload this as a separate file labeled 'Revised Manuscript with Track Changes'.
- An unmarked version of your revised paper without tracked changes. You should upload this as a separate file labeled 'Manuscript'.

If you would like to make changes to your financial disclosure, please include your updated statement in your cover letter. Guidelines for resubmitting your figure files are available below the reviewer comments at the end of this letter.

If applicable, we recommend that you deposit your laboratory protocols in protocols.io to enhance the reproducibility of your results. Protocols.io assigns your protocol its own identifier (DOI) so that it can be cited independently in the future. For instructions see: https://journals.plos.org/plosone/s/submission-guidelines#loc-laboratory-protocols. Additionally, PLOS ONE offers an option for publishing peer-reviewed Lab Protocol articles, which describe protocols hosted on protocols.io. Read more information on sharing protocols at https://plos.org/protocols?utm_medium=editorial-email&utm_source=authorletters&utm_campaign=protocols.

We look forward to receiving your revised manuscript.

Kind regards,

Muniyandi Nagarajan

Academic Editor

PLOS ONE

Journal Requirements:

When submitting your revision, we need you to address these additional requirements.

1. Please ensure that your manuscript meets PLOS ONE's style requirements, including those for file naming. The PLOS ONE style templates can be found at

https://journals.plos.org/plosone/s/file?id=wjVg/PLOSOne_formatting_sample_main_body.pdf and

https://journals.plos.org/plosone/s/file?id=ba62/PLOSOne_formatting_sample_title_authors_affiliations.pdf

2. Please note that PLOS One has specific guidelines on code sharing for submissions in which author-generated code underpins the findings in the manuscript. In these cases, we expect all author-generated code to be made available without restrictions upon publication of the work. Please review our guidelines at https://journals.plos.org/plosone/s/materials-and-software-sharing#loc-sharing-code and ensure that your code is shared in a way that follows best practice and facilitates reproducibility and reuse.

3. Your ethics statement should only appear in the Methods section of your manuscript. If your ethics statement is written in any section besides the Methods, please delete it from any other section.

4. Please note that your Data Availability Statement is currently missing the DOI/accession number of each dataset OR a direct link to access each database. If your manuscript is accepted for publication, you will be asked to provide these details on a very short timeline. We therefore suggest that you provide this information now, though we will not hold up the peer review process if you are unable.

5. Please upload a new copy of Figure 2 as the detail is not clear. Please follow the link for more information:  https://journals.plos.org/plosone/s/figures

6. If the reviewer comments include a recommendation to cite specific previously published works, please review and evaluate these publications to determine whether they are relevant and should be cited. There is no requirement to cite these works unless the editor has indicated otherwise.

[Note: HTML markup is below. Please do not edit.]

Reviewers' comments:

Reviewer's Responses to Questions

**Comments to the Author**

1. Is the manuscript technically sound, and do the data support the conclusions?

The manuscript must describe a technically sound piece of scientific research with data that supports the conclusions. Experiments must have been conducted rigorously, with appropriate controls, replication, and sample sizes. The conclusions must be drawn appropriately based on the data presented.

Reviewer #1: Partly

Reviewer #2: Yes

**********

2. Has the statistical analysis been performed appropriately and rigorously?

Reviewer #1: Yes

Reviewer #2: Yes

**********

3. Have the authors made all data underlying the findings in their manuscript fully available?

The PLOS Data policy requires authors to make all data underlying the findings described in their manuscript fully available without restriction, with rare exception (please refer to the Data Availability Statement in the manuscript PDF file). The data should be provided as part of the manuscript or its supporting information, or deposited to a public repository. For example, in addition to summary statistics, the data points behind means, medians and variance measures should be available. If there are restrictions on publicly sharing data—e.g. participant privacy or use of data from a third party—those must be specified.

Reviewer #1: No

Reviewer #2: Yes

**********

4. Is the manuscript presented in an intelligible fashion and written in standard English?

PLOS ONE does not copyedit accepted manuscripts, so the language in submitted articles must be clear, correct, and unambiguous. Any typographical or grammatical errors should be corrected at revision, so please note any specific errors here.

Reviewer #1: No

Reviewer #2: Yes

**********

5. Review Comments to the Author

Please use the space provided to explain your answers to the questions above. You may also include additional comments for the author, including concerns about dual publication, research ethics, or publication ethics. (Please upload your review as an attachment if it exceeds 20,000 characters)

Reviewer #1: This manuscript investigates genetic diversity, population structure, and genomic regions under selection in Iranian (IB) and Afghan (AB) Baluchi sheep using Illumina OvineSNP50K data. The study employs standard and appropriate methods (PCA, ADMIXTURE, LD-based Ne estimation, FST, XP-EHH, GO/KEGG analysis), and the topic is relevant for livestock genomics and conservation of indigenous breeds. The manuscript contains interesting biological insights, and the comparison of Iranian and Afghan Baluchi sheep (populations that share a common ancestry but differ substantially in breeding history) is valuable.

However, I have several concerns that must be addressed before the manuscript is suitable for publication. The most important limitations relate to the very small and unbalanced sample size for the Afghan Baluchi population, source of the Iranian Baluchi used in this study not sufficiently representing the actual population, potential overinterpretation of selection signals, and insufficient discussion of statistical power and false positives. In addition, several methodological descriptions require clarification, and the English language requires careful revision for clarity and readability.

Below are my comments on each section:

1. Sample size imbalance (IB n=86 vs AB n=15) and statistical power

The Afghan Baluchi population is represented by only 15 animals, while the Iranian sample includes 86. This imbalance substantially affects:

- allele frequency estimates (critical for FST),

- haplotype-based statistics (XP-EHH),

- LD estimation,

- and effective population size (Ne) inference.

The manuscript does not sufficiently discuss how these methodological limitations may influence the results, particularly for the AB population. The small sample size may also inflate apparent within-population diversity or artificially alter LD decay patterns.

In particular, the manuscript lacks adequate detail on how the two sample sets represent their respective populations. The sample size for AB is too small to be considered representative of a population. Although the IB sample size is large enough to represent the flock from which it was drawn, it cannot be assumed to represent the wider population. The authors also claim that the sampled animals are unrelated, but they do not explain how 86 animals from a single flock, bred for many decades, can be unrelated. It is unclear how many generations of pedigree data were examined to assess relatedness.

Recommendation: Please add a detailed discussion of the limitations introduced by the sample sizes, especially with respect to FST variance, XP-EHH power, and Ne precision. This is crucial for interpreting the robustness of the AB results. It should also clearly demonstrate the IB samples are form a single flock at a breeding station which may not represent a much larger population in a vast geographical region. The authors should indicate the methodology they used to assess the relationship between sampled animals.

2. Selection thresholds and multiple testing

Significance thresholds appear to be defined as the top 1% for Win5FST and |XP-EHH| ≥ 3 for XP-EHH. However:

• The rationale for these choices is not clearly presented.

• No multiple testing correction is attempted or discussed.

• Because of the small AB sample, false positives are likely.

Recommendation: Provide justification for the chosen cutoffs (e.g., empirical distributions, commonly used thresholds in the literature). Acknowledge that these represent heuristic thresholds and may include false positives.

3. Interpretation of Ne and LD results

The manuscript states that IB has slightly lower diversity but higher recent effective population size compared to AB. This pattern is counterintuitive and requires additional explanation.

Recommendation: Expand the discussion of how LD-based Ne behaves under selection pressure, differences in sample sizes, and differences in historical demography. Clarify whether differences in SNP retention after QC affected estimates in either group.

4. Overinterpretation of candidate genes and QTL overlaps

Several candidate genes are linked to long lists of traits (e.g., growth, carcass traits, milk production, reproduction, immune traits, high-altitude adaptation, follicle development, etc.). While these associations may be reported in the literature, not all are relevant to the biological context of Baluchi sheep.

Recommendation:

Please moderate these sections by:

• presenting these genes as “putative candidates”,

• focusing on the traits that are biologically plausible in Baluchi populations,

• reducing the length of gene-by-gene literature reviews,

• and adding a statement acknowledging the limitations of inferring function from QTL overlap.

5. Novelty and relation to previous work

Some earlier studies of sheep in the region (e.g., Eydivandi et al. 2021; Barani et al. 2023; Taheri et al. 2024) have already identified selection signatures in Baluchi sheep.

Recommendation: Clarify more explicitly what novel insight this manuscript contributes beyond earlier work , for example, whether the novelty lies in the direct comparison of IB and AB or the combined use of FST, XP-EHH, and QTL integration.

6. English language and clarity

There are numerous grammatical errors, missing words, and awkward phrasings throughout the manuscript (examples include missing subjects such as in “To assess genetic diversity… calculated several metrics” or inconsistent verb forms). I strongly recommend that the entire manuscript be revised by a fluent English speaker or professional editing service.

7. Methods clarity

• The description of the Win5FST approach should indicate whether sliding windows overlap and how they were defined (by 5 SNPs rather than a fixed BP window).

• LD analysis: specify exact distance intervals used for r² vs distance curves.

• ADMIXTURE: include the sample size for each group in the figure or caption.

8. Figures and tables

• PCA and ADMIXTURE figures should include clearer legends and axes.

• Supplementary tables (S2–S5) should include chromosome positions, SNP counts, and window sizes.

9. Terminology consistency

The manuscript alternates between “breed”, “population”, and “race.” Please standardize terminology.

10. Ethics and ARRIVE guidelines

Because the genotypes were obtained from previously published studies, clarify whether ARRIVE guidelines apply to the original sampling or to the present analysis.

Reviewer #2: This manuscript investigates the genetic diversity, population structure, and selection signatures in Iranian (n=86) and Afghan (n=15) Baluchi sheep using the Illumina Ovine SNP50K Beadchip array. The authors employ ADMIXTURE, FST, and XP-EHH analyses to assess genetic differentiation and identify genomic regions under selection, with a focus on economically important traits. The study finds moderate genetic diversity in both populations, clear genetic separation between Iranian and Afghan sheep, and candidate genes linked to traits such as body weight and milk yield. The work aims to inform breeding and conservation strategies for Baluchi sheep.

Minor comments:

Abstract:

highlight the following in the abstract:

1.Sample size imbalance (IB n=86 vs. AB n=15) reduces power and may bias structure and selection scans.

2.Signals were defined by top 1% thresholds (and XP-EHH ±3) without genome-wide multiple-testing correction; results require validation.

3.Use of the Ovine 50K array can introduce ascertainment bias; analyses relied on 38,193 shared SNPs, which may miss population-specific variants.

Methods:

1.State how unrelatedness was verified (e.g., KING/IBD thresholds), not just asserted as “unrelated.” Provide counts of animals and SNPs removed at each QC step per population (not just thresholds).

2.Confirm genome assembly build used (Oar_v4.0 vs. ARS-UI_Ramb_v2.0, etc.) and SNP coordinates mapping.

3.Describe phasing/imputation pipeline used for XP‑EHH (e.g., BEAGLE/SHAPEIT parameters, reference panel if any); XP‑EHH requires phased haplotypes—this is not currently described.

4. Provide ADMIXTURE cross-validation details (folds, random seed).

5. Define distance binning and how standard errors across bins were computed (and will be plotted).

6. XP‑EHH: detail normalization (per-chromosome or genome-wide), p-value computation from standardized scores, and how significant regions were called (beyond |Z|>3).

7. For GO/KEGG, report multiple-testing correction method (e.g., BH-FDR) and cutoff, not just nominal P-values.

8. full Software versions and environment are required. Already partially listed: PLINK v1.9, ADMIXTURE v1.3.0, SNeP v1.1, rehh (no version), R packages (ggfortify, pheatmap). Add: OS, R version, package versions, command flags, seeds.

Discussion:

1.explicitly address the small Afghan sample size: Power limitations and higher variance in AB estimates (n=15), potential bias in ADMIXTURE, FST, and XP‑EHH, and risk of false negatives in AB.

2.The possibility that some signals reflect drift and demographic history rather than selection; interpret candidate gene links with caution and frame them as hypotheses for validation.

Figures:

1. PCA: add % variance on axes (already in text) and 95% confidence ellipses by population; include LD-pruning note in legend.

2. Heatmap/NJ tree: include bootstrap support for key nodes; specify distance metric and clustering method in caption.

3. LD decay: plot mean r2 per bin with ribbons showing SE or 95% CI; report number of pairwise comparisons per bin.

4. Ne trajectories: include shaded 95% CIs (e.g., from bootstrap over SNP blocks) and note SNeP assumptions in legend.

5. Manhattan (FST, XP‑EHH): add multiple-testing thresholds (e.g., FDR line) and include QQ plots for each scan in Supplementary to assess inflation; annotate top loci with gene names.

Please double check the quality of plots before publication.

**********

6. PLOS authors have the option to publish the peer review history of their article (what does this mean?). If published, this will include your full peer review and any attached files.

If you choose “no”, your identity will remain anonymous but your review may still be made public.

**Do you want your identity to be public for this peer review?**   For information about this choice, including consent withdrawal, please see our Privacy Policy.

Reviewer #1: No

Reviewer #2: **Yes:**   Dr Masoud Shirali

**********

[NOTE: If reviewer comments were submitted as an attachment file, they will be attached to this email and accessible via the submission site. Please log into your account, locate the manuscript record, and check for the action link "View Attachments". If this link does not appear, there are no attachment files.]

To ensure your figures meet our technical requirements, please review our figure guidelines: https://journals.plos.org/plosone/s/figures

You may also use PLOS’s free figure tool, NAAS, to help you prepare publication quality figures: https://journals.plos.org/plosone/s/figures#loc-tools-for-figure-preparation.

NAAS will assess whether your figures meet our technical requirements by comparing each figure against our figure specifications.

# Author comment: Author response to Decision Letter 1

**DOI:** 10.1371/journal.pone.0350262.r002

24 Dec 2025

Dear Editorial Manager

Warm Greeting

Thank you for allowing us to submit a revised draft of the manuscript "Genetic diversity, population structure, and combined detection of selection signatures in Iranian versus Afghan Baluchi sheep" for publication in the PLOS ONE Journal. We appreciate the time and effort you and the reviewers dedicated to providing feedback on our manuscript and are grateful for the insightful comments and valuable improvements to our paper. We have incorporated most of the suggestions made by the reviewers. These changes are in red font in the manuscript. You may also find below a point-by-point response to the reviewers’ comments and concerns in blue.

Reviewers' Comments to the Authors: I have separated my responses to the reviewers’ comments into several categories to achieve an integrated approach.

Editor in Chief

1. Please ensure that your manuscript meets PLOS ONE's style requirements, including those for file naming. The PLOS ONE style templates can be found at https://journals.plos.org/plosone/s/file?id=wjVg/PLOSOne_formatting_sample_main_body.pdf and https://journals.plos.org/plosone/s/file?id=ba62/PLOSOne_formatting_sample_title_authors_affiliations.pdf

Author response: Thank you for pointing this out. We have carefully revised the manuscript and supporting information files to ensure they adhere to PLOS ONE’s style requirements, including formatting and file naming conventions, according to the official templates and recent published papers.

2. Please note that PLOS One has specific guidelines on code sharing for submissions in which author-generated code underpins the findings in the manuscript. In these cases, we expect all author-generated code to be made available without restrictions upon publication of the work. Please review our guidelines at https://journals.plos.org/plosone/s/materials-and-software-sharing#loc-sharing-code and ensure that your code is shared in a way that follows best practice and facilitates reproducibility and reuse.

Author response: We thank the reviewer for highlighting the PLOS ONE code-sharing guidelines. All author-generated code underlying the key findings of this manuscript, including quality control (PLINK), population structure (PCA, Admixture), genetic diversity metrics, LD decay, Ne estimation (SNeP), FST, and XP-EHH (rehh) analyses, has been consolidated into a single, comprehensive supplementary file (S1 File) and made openly available without restrictions.

3. Your ethics statement should only appear in the Methods section of your manuscript. If your ethics statement is written in any section besides the Methods, please delete it from any other section.

Author response: We appreciate the editor’s guidance. We have carefully checked the manuscript and confirm that the ethics statement is present only in the Methods section and did not appear elsewhere; therefore, no revisions were required regarding this point.

4. Please note that your Data Availability Statement is currently missing the DOI/accession number of each dataset OR a direct link to access each database. If your manuscript is accepted for publication, you will be asked to provide these details on a very short timeline. We therefore suggest that you provide this information now, though we will not hold up the peer review process if you are unable.

Author response: After negotiating with all authors, we decided to upload our data file as supporting information. Hope it helps.

5. Please upload a new copy of Figure 2 as the details are not clear. Please follow the link for more information: https://journals.plos.org/plosone/s/figures

Author response: A new, higher-quality version of Figure 2 has been prepared according to the PLOS ONE figure guidelines and has been uploaded to replace the previous version.

6. If the reviewer comments include a recommendation to cite specific previously published works, please review and evaluate these publications to determine whether they are relevant and should be cited. There is no requirement to cite these works unless the editor has indicated otherwise.

Author response: We appreciate the editor’s guidance. We have evaluated all reviewer-recommended references and incorporated the ones that are relevant to our study into the revised manuscript, where appropriate.

Reviewer 1

1. Sample size imbalance (IB n=86 vs AB n=15) and statistical power The Afghan Baluchi population is represented by only 15 animals, while the Iranian sample includes 86. This imbalance substantially affects:

- allele frequency estimates (critical for FST),

- haplotype-based statistics (XP-EHH),

- LD estimation,

- and effective population size (Ne) inference.

The manuscript does not sufficiently discuss how these methodological limitations may influence the results, particularly for the AB population. The small sample size may also inflate apparent within-population diversity or artificially alter LD decay patterns. In particular, the manuscript lacks adequate detail on how the two sample sets represent their respective populations. The sample size for AB is too small to be considered representative of a population. Although the IB sample size is large enough to represent the flock from which it was drawn, it cannot be assumed to represent the wider population. The authors also claim that the sampled animals are unrelated, but they do not explain how 86 animals from a single flock, bred for many decades, can be unrelated. It is unclear how many generations of pedigree data were examined to assess relatedness.

Recommendation: Please add a detailed discussion of the limitations introduced by the sample sizes, especially with respect to FST variance, XP-EHH power, and Ne precision. This is crucial for interpreting the robustness of the AB results. It should also clearly demonstrate the IB samples are form a single flock at a breeding station which may not represent a much larger population in a vast geographical region. The authors should indicate the methodology they used to assess the relationship between sampled animals.

Author response: We thank the reviewer for their insightful comments regarding sample size imbalance and population representation. We fully acknowledge the limitations introduced by the smaller AB sample size (n=15) compared to IB (n=86), which can increase variance in allele frequency estimates, FST, XP-EHH power, LD patterns, and Ne precision, particularly for the AB population. These limitations have now been explicitly discussed in a new paragraph in the Discussion section (lines 413-429).

Regarding population representation, we clarify that the IB samples originate from a single high-purity breeding station flock, which was specifically selected due to the lack of purity information for Baluchi populations scattered across eastern and southern regions of Iran outside the station. These field populations are typically not maintained as purebreds, making the station flock the most reliable source for a representative and pure IB genetics. We now emphasize that while the IB sample (n=86) adequately represents this managed flock, it may not fully capture the broader geographic diversity of Iranian Baluchi sheep (Methods, lines 153-161).

For the Afghan Baluchi (AB) samples, we acknowledge that n=15 is exploratory due to logistical challenges: unlike Iran, Afghanistan lacks dedicated Baluchi breeding centers, and these sheep are raised traditionally in mixed flocks. Identifying and collecting additional purebred AB samples proved infeasible within the scope of this study. The AB samples were collected from multiple regions in Herat province, minimizing relatedness.

To address relatedness concerns, we performed IBD analysis using PLINK (PI_HAT). The AB samples showed very low PI-HAT values due to sampling from diverse regions. For IB samples, only one pair exceeded 0.5% PI-HAT; we removed one individual from this pair and conducted sensitivity analyses, which showed no substantial changes in results (Methods, lines 173-177).

To address statistical concerns, we used the θ estimator for FST, which is robust to sample size imbalances and reduces bias from unequal allele frequency variance [ref: Weir & Cockerham 1984; Bhatia et al. 2013]. Similar sample sizes have been successfully applied for XP-EHH, LD decay, and Ne estimation in sheep population studies [Jin et al., 2024; Zhu et al., 2023; Khalkhali-Evrigh et al., 2025]. Despite these limitations, the consistent selection signals across methods suggest robust patterns warranting further investigation with larger samples.

2. Selection thresholds and multiple testing Significance thresholds appear to be defined as the top 1% for Win5FST and |XP-EHH| ≥ 3 for XP-EHH. However:

• The rationale for these choices is not clearly presented.

• No multiple testing correction is attempted or discussed.

• Because of the small AB sample, false positives are likely.

Recommendation: Provide justification for the chosen cutoffs (e.g., empirical distributions, commonly used thresholds in the literature). Acknowledge that these represent heuristic thresholds and may include false positives.

Author response: We thank the reviewer for raising this important point regarding selection thresholds and multiple testing. We have now clarified the rationale for our thresholds in the revised Methods section (lines 246-248) and acknowledged their heuristic nature in the Discussion (lines 487-491).

The top 1% threshold for Win5FST and |XP-EHH| ≥ 3 were selected following commonly used empirical cutoffs in sheep and livestock selection signature studies [Demissie et al., 2025; Abied et al., 2020; Zhang et al., 2022]. These thresholds effectively identify extreme outliers in the empirical genomic distribution, which is the standard exploratory approach for detecting candidate selection regions when prior biological knowledge is limited.

Regarding multiple testing correction, we acknowledge that formal corrections (e.g., Bonferroni) are rarely applied in selection signature scans due to: (1) high linkage disequilibrium causing marker non-independence, (2) the exploratory nature of these analyses aiming to generate hypotheses rather than test them definitively, and (3) substantial power loss from overly conservative adjustments [Sabeti et al. 2007; Vitti et al. 2013]. Similar top 1% and XP-EHH thresholds without multiple testing correction are routinely reported across recent livestock genomic selection studies [Lei et al., 2021; Zhang et al., 2023]. We now explicitly state that these represent heuristic thresholds likely to include false positives, particularly given the small AB sample size, and emphasize that identified regions require experimental validation.

3. Interpretation of Ne and LD results. The manuscript states that IB has slightly lower diversity but a higher recent effective population size compared to AB. This pattern is counterintuitive and requires additional explanation.

Recommendation: Expand the discussion of how LD-based Ne behaves under selection pressure, differences in sample sizes, and differences in historical demography. Clarify whether differences in SNP retention after QC affected estimates in either group.

Author response: We thank the reviewer for highlighting this important point regarding the seemingly counterintuitive Ne and LD patterns between IB and AB populations. We have expanded the Discussion with a dedicated paragraph addressing these factors (lines 449-459). “The apparently higher recent Ne in IB despite lower genetic diversity reflects breeding station management practices and sample size effects on LD-based estimation, rather than true population-level differences. These patterns highlight the importance of interpreting station vs. field population estimates cautiously. Also, QC filtering was identical across populations (same --geno, --maf, --mind, --hwe thresholds), so SNP retention did not differentially affect estimates.

4. Overinterpretation of candidate genes and QTL overlaps. Several candidate genes are linked to long lists of traits (e.g., growth, carcass traits, milk production, reproduction, immune traits, high-altitude adaptation, follicle development, etc.). While these associations may be reported in the literature, not all are relevant to the biological context of Baluchi sheep.

Recommendation: Please moderate these sections by:

• presenting these genes as “putative candidates”,

• focusing on the traits that are biologically plausible in Baluchi populations,

• reducing the length of gene-by-gene literature reviews,

• and adding a statement acknowledging the limitations of inferring function from QTL overlap.

Author response: We agree with the reviewer that some of the original descriptions of candidate genes and QTL overlaps were overly extensive and may have suggested stronger functional evidence than is warranted. In the revised manuscript, we now consistently refer to these loci as “putative candidate genes”, have shortened the gene‑by‑gene literature descriptions, and focused our discussion on traits that are biologically plausible and relevant in Baluchi sheep (e.g. growth, fat deposition, adaptation to arid environments, reproduction and disease resistance). We have also added an explicit statement in the Discussion acknowledging the limitations of inferring gene function solely from positional overlap with broad QTL or association regions and emphasizing that our interpretations are hypothesis‑generating rather than causal.

5. Novelty and relation to previous work. Some earlier studies of sheep in the region (e.g., Eydivandi et al. 2021; Barani et al. 2023; Taheri et al. 2024) have already identified selection signatures in Baluchi sheep. Recommendation: Clarify more explicitly what novel insight this manuscript contributes beyond earlier work, for example, whether the novelty lies in the direct comparison of IB and AB or in the combined use of FST, XP-EHH, and QTL integration.

Author response: We appreciate the reviewer’s comment and agree that several previous studies have investigated selection signatures in Baluchi or regional sheep populations (e.g. Eydivandi et al. 2021; Barani et al. 2023). However, those studies mainly compared Baluchi with multiple distinct breeds from Iran, Afghanistan or neighboring countries at a broad inter‑breed level, without a focused genomic comparison between Iranian Baluchi (IB) and Afghan Baluchi (AB), which are believed to share a common ancestral background. In contrast, the novelty of our work lies in (i) a dedicated, pairwise comparison of IB and AB populations to dissect how historical management and breeding in the two countries have shaped divergent selection signatures within the same breed, and (ii) the combined use of complementary statistics (FST and XP‑EHH) together with systematic integration of sheep QTL information, which allows us to link shared and population‑specific selective sweeps to concrete production, adaptation and reproduction traits and to highlight candidate regions for future functional and genomic selection studies.

6. English language and clarity. There are numerous grammatical errors, missing words, and awkward phrasings throughout the manuscript (examples include missing subjects, such as in “To assess genetic diversity… calculated several metrics” or inconsistent verb forms). I strongly recommend that the entire manuscript be revised by a fluent English speaker or a professional editing service.

Author response: Thank the reviewer for highlighting the language issues. We have thoroughly revised the entire manuscript for grammatical accuracy, clarity, and fluency. All changes enhance readability while preserving the original scientific meaning. We believe the manuscript now meets high standards of English proficiency.

7. Methods clarity

• The description of the Win5FST approach should indicate whether sliding windows overlap and how they were defined (by 5 SNPs rather than a fixed BP window).

• LD analysis: specify exact distance intervals used for r² vs distance curves.

• ADMIXTURE: include the sample size for each group in the figure or caption.

Author response: Thank the reviewer for these suggestions to improve methodological transparency. All requested clarifications have been incorporated as detailed

Attachment Submitted filename: Response to Reviewers.docx

# Peer review: Decision Letter 1

**DOI:** 10.1371/journal.pone.0350262.r003

**Authors:** Shamik Polley

22 Apr 2026

PONE-D-25-52267R1

Genetic diversity, population structure, and combined detection of selection signatures in Iranian versus Afghan Baluchi sheep

PLOS One

Dear Dr. Javadmanesh,

Thank you for submitting your manuscript to PLOS ONE. After careful consideration, we feel that it has merit but does not fully meet PLOS ONE’s publication criteria as it currently stands. Therefore, we invite you to submit a revised version of the manuscript that addresses the points raised during the review process.

Please submit your revised manuscript by Jun 06 2026 11:59PM. If you will need more time than this to complete your revisions, please reply to this message or contact the journal office at plosone@plos.org. When you're ready to submit your revision, log on to https://www.editorialmanager.com/pone/ and select the 'Submissions Needing Revision' folder to locate your manuscript file.

Please include the following items when submitting your revised manuscript:

- A letter that responds to each point raised by the academic editor and reviewer(s). You should upload this letter as a separate file labeled 'Response to Reviewers'.
- A marked-up copy of your manuscript that highlights changes made to the original version. You should upload this as a separate file labeled 'Revised Manuscript with Track Changes'.
- An unmarked version of your revised paper without tracked changes. You should upload this as a separate file labeled 'Manuscript'.

If you would like to make changes to your financial disclosure, please include your updated statement in your cover letter. Guidelines for resubmitting your figure files are available below the reviewer comments at the end of this letter.

If applicable, we recommend that you deposit your laboratory protocols in protocols.io to enhance the reproducibility of your results. Protocols.io assigns your protocol its own identifier (DOI) so that it can be cited independently in the future. For instructions see: https://journals.plos.org/plosone/s/submission-guidelines#loc-laboratory-protocols. Additionally, PLOS ONE offers an option for publishing peer-reviewed Lab Protocol articles, which describe protocols hosted on protocols.io. Read more information on sharing protocols at https://plos.org/protocols?utm_medium=editorial-email&utm_source=authorletters&utm_campaign=protocols.

As the corresponding author, your ORCID iD is verified in the submission system and will appear in the published article. PLOS supports the use of ORCID, and we encourage all coauthors to register for an ORCID iD and use it as well. Please encourage your coauthors to verify their ORCID iD within the submission system before final acceptance, as unverified ORCID iDs will not appear in the published article. *Only*   the individual author can complete the verification step; PLOS staff *cannot*   verify ORCID iDs on behalf of authors.

We look forward to receiving your revised manuscript.

Kind regards,

Shamik Polley, M.V.Sc (Veterinary Biochemistry); Ph.D (Genetics)

Academic Editor

PLOS One

Journal Requirements:

1. If the reviewer comments include a recommendation to cite specific previously published works, please review and evaluate these publications to determine whether they are relevant and should be cited. There is no requirement to cite these works unless the editor has indicated otherwise.

2. Please review your reference list to ensure that it is complete and correct. If you have cited papers that have been retracted, please include the rationale for doing so in the manuscript text, or remove these references and replace them with relevant current references. Any changes to the reference list should be mentioned in the rebuttal letter that accompanies your revised manuscript. If you need to cite a retracted article, indicate the article’s retracted status in the References list and also include a citation and full reference for the retraction notice.

[Note: HTML markup is below. Please do not edit.]

Reviewer's Responses to Questions

**Comments to the Author**

1. If the authors have adequately addressed your comments raised in a previous round of review and you feel that this manuscript is now acceptable for publication, you may indicate that here to bypass the “Comments to the Author” section, enter your conflict of interest statement in the “Confidential to Editor” section, and submit your "Accept" recommendation.

Reviewer #2: All comments have been addressed

Reviewer #3: All comments have been addressed

Reviewer #4: All comments have been addressed

**********

2. Is the manuscript technically sound, and do the data support the conclusions?

The manuscript must describe a technically sound piece of scientific research with data that supports the conclusions. Experiments must have been conducted rigorously, with appropriate controls, replication, and sample sizes. The conclusions must be drawn appropriately based on the data presented.

Reviewer #2: Yes

Reviewer #3: Yes

Reviewer #4: Yes

**********

3. Has the statistical analysis been performed appropriately and rigorously?

Reviewer #2: Yes

Reviewer #3: Yes

Reviewer #4: Yes

**********

4. Have the authors made all data underlying the findings in their manuscript fully available?

The PLOS Data policy requires authors to make all data underlying the findings described in their manuscript fully available without restriction, with rare exception (please refer to the Data Availability Statement in the manuscript PDF file). The data should be provided as part of the manuscript or its supporting information, or deposited to a public repository. For example, in addition to summary statistics, the data points behind means, medians and variance measures should be available. If there are restrictions on publicly sharing data—e.g. participant privacy or use of data from a third party—those must be specified.

Reviewer #2: Yes

Reviewer #3: Yes

Reviewer #4: Yes

**********

5. Is the manuscript presented in an intelligible fashion and written in standard English?

PLOS ONE does not copyedit accepted manuscripts, so the language in submitted articles must be clear, correct, and unambiguous. Any typographical or grammatical errors should be corrected at revision, so please note any specific errors here.

Reviewer #2: Yes

Reviewer #3: Yes

Reviewer #4: Yes

**********

6. Review Comments to the Author

Please use the space provided to explain your answers to the questions above. You may also include additional comments for the author, including concerns about dual publication, research ethics, or publication ethics. (Please upload your review as an attachment if it exceeds 20,000 characters)

Reviewer #2: This manuscript presents a well‑executed and meaningful investigation into genetic diversity, population structure, and selection signatures in Iranian and Afghan Baluchi sheep, using robust genomic tools and clear analytical methods. The identification of candidate genes linked to economically important traits is especially valuable and demonstrates both scientific rigor and practical relevance, highlighting the strength and coherence of the study’s design. Overall, the work is well‑structured, clearly written, and provides insights that make it suitable for publication. The current draft has addressed the necessary editorial refinements, and I recommend acceptance for publication.

Reviewer #3: This is an interesting study which investigates the genetic diversity and population structure and notable genetic signatures in two populations of native breed sheep- the Baluchi breed from Iran and Afghanistan.

Having not reviewed the original, none of the comments were mine, but appear to have been addressed. In the main, most of the comments below are just around polishing of the manuscript. I would also suggest a final proof read before re-submission.

Line 65-66-this doesn’t makes sense, please reword

Line 78- worth defining IB and AB in the main text (appreciate that they are defined in the abstract)

Line 90-93- this is a bit of a repeat of what you have above, maybe worth considering consolidating some of this with the bits above

Line 177-178- this is not a complete sentence

Line 200-201- this is not a complete sentence

Table 3 has a large legend with all the abbreviations in, whereas table 2 does not. Can the same be added for table 2 please

Line 566- ‘with the results of the current study’ (add in ‘the’)

Line 608- capital letter needed for Putative

Reviewer #4: The authors have adequately addressed the main points raised in the initial review.

Limitations related to sample size imbalance, statistical power, and LD based Ne estimates are now clearly acknowledged and appropriately discussed.

The interpretation of selection signatures and candidate genes has been substantially moderated and framed as exploratory.

In the Conclusions, it would be appropriate to explicitly state: “These results should be interpreted as reflecting differences between a managed breeding population and a traditional field population rather than national level divergence.”

Overall, the manuscript is technically sound and suitable for publication after minor editorial polishing.

**********

7. PLOS authors have the option to publish the peer review history of their article (what does this mean?). If published, this will include your full peer review and any attached files.

If you choose “no”, your identity will remain anonymous but your review may still be made public.

**Do you want your identity to be public for this peer review?**   For information about this choice, including consent withdrawal, please see our Privacy Policy.

Reviewer #2: **Yes:**   Dr Masoud Shirali

Reviewer #3: No

Reviewer #4: No

**********

[NOTE: If reviewer comments were submitted as an attachment file, they will be attached to this email and accessible via the submission site. Please log into your account, locate the manuscript record, and check for the action link "View Attachments". If this link does not appear, there are no attachment files.]

To ensure your figures meet our technical requirements, please review our figure guidelines: https://journals.plos.org/plosone/s/figures

You may also use PLOS’s free figure tool, NAAS, to help you prepare publication quality figures: https://journals.plos.org/plosone/s/figures#loc-tools-for-figure-preparation.

NAAS will assess whether your figures meet our technical requirements by comparing each figure against our figure specifications.

# Author comment: Author response to Decision Letter 2

**DOI:** 10.1371/journal.pone.0350262.r004

28 Apr 2026

Dear Editorial Manager

Warm Greeting

Thank you for following up this revision and we appreciate the time and effort you and the reviewers spent to provide comments on this manuscript. All of comments were very positive and we have applied all of the suggestions made by the reviewers. These changes are highlighted in yellow in the manuscript.

Journal Requirements:

Please ensure that your manuscript meets PLOS ONE's style requirements, including those for file naming. The PLOS ONE style templates can be found at https://journals.plos.org/plosone/s/file?id=wjVg/PLOSOne_formatting_sample_main_body.pdf and https://journals.plos.org/plosone/s/file?id=ba62/PLOSOne_formatting_sample_title_authors_affiliations.pdf

Author response: Thank you. All of citations are double checked now.

Review Comments to the Author

Reviewer #2: This manuscript presents a well‑executed and meaningful investigation into genetic diversity, population structure, and selection signatures in Iranian and Afghan Baluchi sheep, using robust genomic tools and clear analytical methods. The identification of candidate genes linked to economically important traits is especially valuable and demonstrates both scientific rigor and practical relevance, highlighting the strength and coherence of the study’s design. Overall, the work is well‑structured, clearly written, and provides insights that make it suitable for publication. The current draft has addressed the necessary editorial refinements, and I recommend acceptance for publication.

Author response: Thanks a lot.

Reviewer #3: This is an interesting study which investigates the genetic diversity and population structure and notable genetic signatures in two populations of native breed sheep- the Baluchi breed from Iran and Afghanistan.

Having not reviewed the original, none of the comments were mine, but appear to have been addressed. In the main, most of the comments below are just around polishing of the manuscript. I would also suggest a final proof read before re-submission.

Line 65-66-this doesn’t makes sense, please reword

Line 78- worth defining IB and AB in the main text (appreciate that they are defined in the abstract)

Line 90-93- this is a bit of a repeat of what you have above, maybe worth considering consolidating some of this with the bits above

Line 177-178- this is not a complete sentence

Line 200-201- this is not a complete sentence

Table 3 has a large legend with all the abbreviations in, whereas table 2 does not. Can the same be added for table 2 please

Line 566- ‘with the results of the current study’ (add in ‘the’)

Line 608- capital letter needed for Putative

Author response: We appreciate the reviewer’s comments. We have corrected all suggestions. Regarding Tables 2 and 3, I have checked and font size in these two tables are exactly the same. Therefore I can not change one based on another.

Reviewer #4: The authors have adequately addressed the main points raised in the initial review.

Limitations related to sample size imbalance, statistical power, and LD based Ne estimates are now clearly acknowledged and appropriately discussed.

The interpretation of selection signatures and candidate genes has been substantially moderated and framed as exploratory.

In the Conclusions, it would be appropriate to explicitly state: “These results should be interpreted as reflecting differences between a managed breeding population and a traditional field population rather than national level divergence.”

Overall, the manuscript is technically sound and suitable for publication after minor editorial polishing.

Author response: We thank the reviewer for raising this important point regarding conclusion. This statement has been added now.

Best regards,

Ali Javadmanesh, PhD

Associate Professor

Department of Animal Science,

Faculty of Agriculture,

Ferdowsi University of Mashhad

Attachment Submitted filename: Response_to_Reviewers_auresp_2.docx

# Peer review: Decision Letter 2

**DOI:** 10.1371/journal.pone.0350262.r005

**Authors:** Shamik Polley

13 May 2026

Genetic diversity, population structure, and combined detection of selection signatures in Iranian versus Afghan Baluchi sheep

PONE-D-25-52267R2

Dear Dr. Javadmanesh,

We’re pleased to inform you that your manuscript has been judged scientifically suitable for publication and will be formally accepted for publication once it meets all outstanding technical requirements.

Within one week, you’ll receive an e-mail detailing the required amendments. When these have been addressed, you’ll receive a formal acceptance letter and your manuscript will be scheduled for publication.

An invoice will be generated when your article is formally accepted. Please note, if your institution has a publishing partnership with PLOS and your article meets the relevant criteria, all or part of your publication costs will be covered. Please make sure your user information is up-to-date by logging into Editorial Manager at Editorial Manager® and clicking the ‘Update My Information' link at the top of the page. For questions related to billing, please contact billing support.

If your institution or institutions have a press office, please notify them about your upcoming paper to help maximize its impact. If they’ll be preparing press materials, please inform our press team as soon as possible -- no later than 48 hours after receiving the formal acceptance. Your manuscript will remain under strict press embargo until 2 pm Eastern Time on the date of publication. For more information, please contact onepress@plos.org.

Kind regards,

Shamik Polley, M.V.Sc (Veterinary Biochemistry); Ph.D (Genetics)

Academic Editor

PLOS One

Additional Editor Comments (optional):

Reviewers' comments:

Reviewer's Responses to Questions

**Comments to the Author**

1. If the authors have adequately addressed your comments raised in a previous round of review and you feel that this manuscript is now acceptable for publication, you may indicate that here to bypass the “Comments to the Author” section, enter your conflict of interest statement in the “Confidential to Editor” section, and submit your "Accept" recommendation.

Reviewer #3: All comments have been addressed

Reviewer #4: All comments have been addressed

**********

2. Is the manuscript technically sound, and do the data support the conclusions?

The manuscript must describe a technically sound piece of scientific research with data that supports the conclusions. Experiments must have been conducted rigorously, with appropriate controls, replication, and sample sizes. The conclusions must be drawn appropriately based on the data presented.

Reviewer #3: Yes

Reviewer #4: Yes

**********

3. Has the statistical analysis been performed appropriately and rigorously?

Reviewer #3: Yes

Reviewer #4: Yes

**********

4. Have the authors made all data underlying the findings in their manuscript fully available?

The PLOS Data policy requires authors to make all data underlying the findings described in their manuscript fully available without restriction, with rare exception (please refer to the Data Availability Statement in the manuscript PDF file). The data should be provided as part of the manuscript or its supporting information, or deposited to a public repository. For example, in addition to summary statistics, the data points behind means, medians and variance measures should be available. If there are restrictions on publicly sharing data—e.g. participant privacy or use of data from a third party—those must be specified.

Reviewer #3: Yes

Reviewer #4: Yes

**********

5. Is the manuscript presented in an intelligible fashion and written in standard English?

PLOS ONE does not copyedit accepted manuscripts, so the language in submitted articles must be clear, correct, and unambiguous. Any typographical or grammatical errors should be corrected at revision, so please note any specific errors here.

Reviewer #3: Yes

Reviewer #4: Yes

**********

6. Review Comments to the Author

Please use the space provided to explain your answers to the questions above. You may also include additional comments for the author, including concerns about dual publication, research ethics, or publication ethics. (Please upload your review as an attachment if it exceeds 20,000 characters)

Reviewer #3: I wish to thank the authors for addressing all my comments. Manuscript reads well and I have no further comments

Reviewer #4: The document is suitable for publication for the following reasons:

-The manuscript is technically sound and methodologically robust. The study design is appropriate for addressing the stated research questions, and the methods are described in sufficient detail to allow reproducibility. The data presented clearly support the authors’ conclusions, and there is a strong logical connection between the results and the interpretations offered.

-The statistical analyses have been performed appropriately and with a high level of rigor. The choice of statistical tests is well justified, assumptions are adequately addressed, and the analyses are correctly applied and clearly reported. The results of the statistical analyses are transparent and contribute meaningfully to the strength and credibility of the findings.

-All data underlying the findings reported in the manuscript appear to be fully available and accessible, either within the manuscript itself or through the indicated supplementary materials or data repositories. This commitment to data transparency aligns well with current best practices in research and enhances the reproducibility and reliability of the study.

-The manuscript is presented in an intelligible and well-organized manner and is written in clear, standard English. The structure follows a logical progression, and the arguments are easy to follow. Terminology is used consistently, and the text is accessible to readers within the field. Overall, the manuscript demonstrates a high standard of scholarly writing.

-With respect to human research and publication ethics, the study appropriately addresses ethical considerations, including relevant approvals and informed consent procedures. There are no apparent concerns regarding research integrity, dual publication, or ethical compliance.

In summary, this is a well-conducted and clearly presented study that makes a valuable contribution to the field.

**********

7. PLOS authors have the option to publish the peer review history of their article (what does this mean?). If published, this will include your full peer review and any attached files.

If you choose “no”, your identity will remain anonymous but your review may still be made public.

**Do you want your identity to be public for this peer review?**   For information about this choice, including consent withdrawal, please see our Privacy Policy.

Reviewer #3: No

Reviewer #4: No

**********

# Editor report: Acceptance letter

**DOI:** 10.1371/journal.pone.0350262.r006

**Authors:** Shamik Polley

PONE-D-25-52267R2

PLOS One

Dear Dr. Javadmanesh,

I'm pleased to inform you that your manuscript has been deemed suitable for publication in PLOS One. Congratulations! Your manuscript is now being handed over to our production team.

At this stage, our production department will prepare your paper for publication. This includes ensuring the following:

* All references, tables, and figures are properly cited

* All relevant supporting information is included in the manuscript submission,

* There are no issues that prevent the paper from being properly typeset

You will receive further instructions from the production team, including instructions on how to review your proof when it is ready. Please keep in mind that we are working through a large volume of accepted articles, so please give us a few days to review your paper and let you know the next and final steps.

Lastly, if your institution or institutions have a press office, please let them know about your upcoming paper now to help maximize its impact. If they'll be preparing press materials, please inform our press team within the next 48 hours. Your manuscript will remain under strict press embargo until 2 pm Eastern Time on the date of publication. For more information, please contact onepress@plos.org.

You will receive an invoice from PLOS for your publication fee after your manuscript has reached the completed accept phase. If you receive an email requesting payment before acceptance or for any other service, this may be a phishing scheme. Learn how to identify phishing emails and protect your accounts at https://explore.plos.org/phishing.

If we can help with anything else, please email us at customercare@plos.org.

Thank you for submitting your work to PLOS ONE and supporting open access.

Kind regards,

PLOS ONE Editorial Office Staff

on behalf of

Dr. Shamik Polley

Academic Editor

PLOS One
